# Searching for Glycosylated Natural Products in Actinomycetes and Identification of Novel Macrolactams and Angucyclines

**DOI:** 10.3389/fmicb.2018.00039

**Published:** 2018-01-30

**Authors:** Mónica G. Malmierca, Lorena González-Montes, Ignacio Pérez-Victoria, Carlos Sialer, Alfredo F. Braña, Raúl García Salcedo, Jesús Martín, Fernando Reyes, Carmen Méndez, Carlos Olano, José A. Salas

**Affiliations:** ^1^Departamento de Biología Funcional e Instituto Universitario de Oncología del Principado de Asturias, Universidad de Oviedo, Oviedo, Spain; ^2^Instituto de Investigación Sanitaria del Principado de Asturias, Oviedo, Spain; ^3^Fundación MEDINA, Parque Tecnológico de Ciencias de la Salud, Granada, Spain

**Keywords:** polyketides, *Streptomyces*, deoxy sugars, glycosylation, genome mining

## Abstract

Many bioactive natural products are glycosylated compounds in which the sugar components usually participate in interaction and molecular recognition of the cellular target. Therefore, the presence of sugar moieties is important, in some cases essential, for bioactivity. Searching for novel glycosylated bioactive compounds is an important aim in the field of the research for natural products from actinomycetes. A great majority of these sugar moieties belong to the 6-deoxyhexoses and share two common biosynthetic steps catalyzed by a NDP-*D*-glucose synthase (GS) and a NDP-*D*-glucose 4,6-dehydratase (DH). Based on this fact, seventy one *Streptomyces* strains isolated from the integument of ants of the Tribe *Attini* were screened for the presence of biosynthetic gene clusters (BGCs) for glycosylated compounds. Total DNAs were analyzed by PCR amplification using oligo primers for GSs and DHs and also for a NDP-*D*-glucose-2,3-dehydratases. Amplicons were used in gene disruption experiments to generate non-producing mutants in the corresponding clusters. Eleven mutants were obtained and comparative dereplication analyses between the wild type strains and the corresponding mutants allowed in some cases the identification of the compound coded by the corresponding cluster (lobophorins, vicenistatin, chromomycins and benzanthrins) and that of two novel macrolactams (sipanmycin A and B). Several strains did not show UPLC differential peaks between the wild type strain and mutant profiles. However, after genome sequencing of these strains, the activation of the expression of two clusters was achieved by using nutritional and genetic approaches leading to the identification of compounds of the cervimycins family and two novel members of the warkmycins family. Our work defines a useful strategy for the identification new glycosylated compounds by a combination of genome mining, gene inactivation experiments and the activation of silent biosynthetic clusters in *Streptomyces* strains.

## Introduction

Natural products are an important source of clinical and pharmaceutical compounds and they also contribute with compounds showing agricultural and veterinary applications. A large proportion of natural products are synthesized by microorganisms which constitute a prolific source of structurally diverse bioactive metabolites and have yielded some of the most important products of the pharmaceutical industry. Within microorganisms, the actinomycete family constitutes a good reservoir of natural products: approximately two-thirds of all natural products are produced by members of this family (Bérdy, 2005; Demain and Sanchez, 2009). Most of the natural products identified have emerged from large screening programs carried out by pharmaceutical companies. However, in the last years, the interest in the search for natural products has in some way declined. One of the reasons is the redundancy in the discovery of natural products with many of the newly isolated compounds corresponding to already known compounds. However, the growing rate of multi-resistant pathogens, the appearance of new ones and the need for more effective chemotherapeutic agents, together with the development of DNA sequencing technologies, are facilitating a return to the search for bioactive natural products from actinomycetes (Newman and Cragg, 2016; Zhang et al., 2017a). From the sequencing of the first complete genome of actinomycetes, up to date more than 1,000 genomes have been sequenced and annotated (Gomez-Escribano et al., 2016). With the bioinformatic analyses of these genomes became evident that actinomycetes carry the genetic potential to produce many more natural products than those detected under laboratory conditions. It is frequent to observe that the sequenced genomes of actinomycetes contain between 20 and 30 biosynthetic gene clusters (BGCs) for secondary metabolites production. Many of these BGCs are not expressed (or are very low expressed) under laboratory cultivation conditions and are designated as “silent” clusters. Several strategies have been applied to awake these clusters including nutritional (Bode et al., 2002) and genetic approaches (Challis, 2008; Laureti et al., 2011; Olano et al., 2014; Thanapipatsiri et al., 2016; Zhang et al., 2017b) and ribosomal engineering (Ochi et al., 2004; Ochi and Hosaka, 2013).

Many BGCs code for glycosylated metabolites (Weymouth-Wilson, 1997). Therapeutically important antibiotics (erythromycin), antifungals (amphotericin B), antiparasitics (avermectins) and anticancer drugs (doxorubicin) contain sugars attached to the aglycone core. They contribute to create structural biodiversity in natural products and they participate in the interaction between the drug and the cellular target. Consequently, their presence is important, in many cases essential, for the biological activity of many bioactive natural products (Méndez and Salas, 2001; Salas and Méndez, 2005). Most of these sugars are 6-deoxyhexoses (6DOH), of which more than 70 different forms have been identified (Thorson et al., 2001; Méndez et al., 2008; Thibodeaux et al., 2008). Its biosynthesis shares the first two steps where *D*-glucose-1-phosphate is converted into NDP-*D*-glucose by a NDP-*D*-glucose synthase (GS) and then dehydrated by a NDP-*D*-glucose-4,6-dehydratase (DH). Afterwards, the common intermediate NDP-4-keto-6-deoxy-*D*-glucose is subjected to several modification reactions, including deoxygenations (frequently by a 2,3-dehydratase; 2,3DH), methylations, transaminations, etc., to yield the enormous variety of the 6DOH family (Salas and Méndez, 2005).

In recent years, the search for novel bioactive compounds has followed a new strategy trying to overcome the problem of re-discovery of already known compounds: the isolation of the producing organisms in poorly characterized environments such as marine sediments or in association with insects (Poulsen et al., 2011). Leaf-cutting ants of the Tribe *Attini* have attracted particular attention because they cultivate a fungus in specialized chambers of their nests. The ants cut leaves into small pieces and feed them to fungus, which will serve as their source of food. To protect themselves from fungal infections symbiotic microorganisms are located on the integument of these ants. Although it was initially assumed that they were *Pseudonocardia* spp. (Currie et al., 1999), more recent evidence indicates that more than a single symbiont exist in these ants, and microorganisms as *Streptomyces* and *Burkholderia* are now thought to be involved in this multitrophic interaction (Kost et al., 2007; Mueller et al., 2008).

Different targeted screenings have been applied in the last years for the discovery of novel bioactive natural products. These include bioassay-guided approaches (Amagata et al., 2012; Wong et al., 2012; Hassig et al., 2014; Shanbhag et al., 2015), search of self-protection mechanisms of antibiotic producers (Thaker et al., 2013), dereplication strategies of natural products using high-resolution mass spectrometry (MS) (Chervin et al., 2017), tandem MS for the search of particular structural features such as glycosylated compounds (Kersten et al., 2013b), MS-based proteomics of expressed enzymes such as polyketide synthases (PKSs) and non-ribosomal peptide synthetases (NRPSs) involved in natural product biosynthesis (Chen et al., 2016), and different genetic approaches. The latter includes transcriptome analysis by RNA sequencing (Jones et al., 2017), sequencing and mining of microbial genomes or metagenomes in search of specific gene homologs (Chang and Brady, 2013) or new BGCs (Kang, 2017), and the use of polymerase chain reaction (PCR)-based screenings of genes encoding enzymes implicated in the biosynthesis of particular structural features of natural products such as NRPS scaffolds (Bakal et al., 2015), PKS cores (Cao et al., 2010; Kang and Brady, 2013) or deoxyhexose moieties (Wu et al., 2013).

In this paper we searched for the presence of glycosylated secondary metabolites in a collection of *Streptomyces* strains isolated from leaf-cutting ants. By a combination of PCR-based screening, MS dereplication and generation of mutants we identified several already known compounds and two novel macrolactams. In addition, by genome sequencing and awakening approaches of BGCs we activate the expression of two clusters for type II polyketides that are not expressed under standard laboratory conditions leading to the identification of two novel angucylines.

## Materials and Methods

### Bacterial Strains and Culture Conditions

A collection of seventy one *Streptomyces* strains was used in this work. For sporulation, strains were grown for 7 days at 30°C on MA plates (Sánchez and Braña, 1996). For metabolite production studies, spores were added to flasks containing 40 ml TSB medium and grown at 30°C and 250 rpm. After 24 h, this culture was used to inoculate 50 mL of five different media to a final OD_600*nm*_ = 0.2: R5A medium (Fernández et al., 1998); SM medium (Zhang et al., 1992); SM10 medium (per liter): 20.9 g MOPS, 11.5 g L-Proline, 23 g glycerol, 0.5 g NaCl, 2.1 g K_2_HPO_4_, 0.28 g Na_2_SO_4_, 1 mL MgSO_4_ 0.2 M, 1 mL CaCl_2_ 0.2 M and 5 mL trace elements [composition per liter: 0.04 g ZnCl_2_, 0.2 g FeCl_3_.6H_2_O, 0.01 g CuCl_2_.2H_2_O, 0.01 g MnCl_2_.4H_2_O, 0.01 g Na_2_B_4_O_7_.10H_2_O, 0.01 g (NH_4_)_6_Mo_7_O_24_.4H_2_O], pH 6.5; SM17 medium (per liter): 2 g glucose, 40 g glycerol, 2 g soluble starch, 5 g soybean flour, 5 g peptone, 5 g yeast extract, 5 g NaCl and 2 g CaCO_3_ in tap water; and SM19 medium (per liter): 40 g tomato juice, 15 g oat flour and 2 g glucose. For intergeneric conjugation, MS medium (Hobbs et al., 1989) was used. *Escherichia coli* DH5α (Hanahan, 1983) was used as host for cloning and *E. coli* ET12567/pUB307 for intergeneric conjugation (ET12567/pUB307) (Kieser et al., 2000) and both were grown in 2×TY medium supplemented, when required, with the appropriate antibiotic.

Culture media were supplemented with antibiotics when plasmid-bearing strains were used: apramycin (100 μg/mL for *E. coli*, 25 μg/mL for *Streptomyces*), kanamycin (25 μg/mL), tetracycline (10 μg/mL), chloramphenicol (25 μg/mL), and/or nalidixic acid (50 μg/mL).

### DNA Manipulation and Vectors

DNA manipulations were performed according to standard procedures for *E. coli* (Sambrook et al., 1989) and *Streptomyces* (Hanahan, 1983). Plasmids used in this work were pOJ260 (Bierman et al., 1992) for gene interruption and pEM4ATc, pSETec (Cano-Prieto et al., 2015) and pOJ260P (Olano et al., 2004) for gene expression under the control of *ermE^∗^* promoter.

pEM4ATc was generated by cloning a BamHI-HindIII fragment containing the apramycin resistance gene *aac(3)IV* from pEFBA (Lozano et al., 2000) (ends were filled by Klenow activity following manufacturer indications) into pEM4T (Menendez et al., 2006) digested with EcoRV.

### Plasmid Construction for Gene Disruption

Probes 2,3DH and GS-DH were obtained by PCR amplification of total DNA with degenerate oligonucleotide pairs PCR-D1a/PCR-D1b (Trefzer et al., 2002) and AG5F/4,6DH2 (Hyun et al., 2000; Ogasawara et al., 2004), respectively (Supplementary Table 12). PCR conditions were: initial denaturation at 94°C, 5 min; 35 cycles of 94°C, 1 min, 60°C, 1 min and 72°C, 1 min and a final extension cycle at 72°C, 10 min. DreamTaq DNA polymerase (Thermo Scientific) and 2.5% dimethyl sulfoxide (DMSO) were used. Amplicons were verified by sequencing, digested with EcoRI/HindIII and subcloned into pOJ260. The resulting plasmids were introduced into *Streptomyces* strains by intergeneric conjugation.

### DNA Sequencing and Analysis

The *Streptomyces* sp. CS057, CS113, CS159 and CS227 chromosomes were sequenced at Department of Biochemistry, University of Cambridge (Cambridge, United Kingdom) using Illumina MiSeq Sequencing technology. *De novo* assemblies were achieved using default parameters in Newbler assembler software version 2.9. Annotation was performed using the PGAAP pipeline^1^ (Angiuoli et al., 2008). Database searching and sequence analysis were carried out with the bioinformatic tool antiSMASH (Blin et al., 2017).

These Whole Genome Shotgun projects have been deposited at DDBJ/ENA/GenBank under the accessions NEVF00000000 (*Streptomyces* sp. CS057), NEVC00000000 (*Streptomyces* sp. CS113), NEVD00000000 (*Streptomyces* sp. CS159) and NEVE01000000 (*Streptomyces* sp. CS227). The versions described in this paper are version NEVF01000000, NEVC01000000, NEVD01000000, and NEVE01000000, respectively.

Gene clusters for warkmycin and cervimycin have been deposited at Minimum Information about a Biosynthetic Gene Cluster (MIBiG) repository (Medema et al., 2015) under the accessions BGC0001438 and BGC0001439, respectively.

### Plasmid Construction for Cluster Activation

For cluster activation in CS113, *cmv22* was expressed under the control of *ermE*^∗^p. A 0.96 kb fragment was amplified with oligonucleotides 113-eSARP78.5b and 113-eSARP78.3b, gel purified, digested with BamHI/EcoRI and cloned into pSETec, pEM4ATc, and pOJ260P, leading to final plasmids pSET113eSARP, pEM113eSARP, and pOJ113eSARP, respectively.

*orf15* of CS159 was amplified with oligonucleotides pErmE6RT15.5b and pErmE6RT15.3b. The resulting 0.79 kb fragment was gel purified, digested with BamHI/EcoRI and cloned into pSETec, pEM4ATc, and pOJ260P, leading to final plasmids pSET159eRT15, pEM159eRT15, and pOJ159eRT15, respectively.

Using genomic DNA of CS227, *orf2* (0.92 kb) and *orf37* (2.8 kb) were amplified with oligonucleotide pairs 227-eSARP2.5/227-eSARP2.3 and 227-eLuxR37.5/227-eLuxR37.3, respectively. Both amplicons were gel purified, digested with BamHI/EcoRI and cloned into pSETec, pEM4ATc, and pOJ260P, leading to final plasmids pSET227eSARP, pEM227eSARP, pOJ227eSARP, pSET227eLuxR, pEM227eLuxR, and pOJ227eLuxR.

*wmc32* of CS057 was amplified with oligonucleotide 57-eReg.5b and 57-eReg.3b. A 0.84 kb fragment was gel purified, digested with XbaI and cloned into pSETec, pEM4ATc, and pOJ260P, leading to final plasmids pSET57eReg, pEM57eReg, and pOJ57eReg, respectively. The correct orientation of *wmc32* was confirmed by restriction enzyme digestion and sequencing.

All amplifications were carried out with the high fidelity polymerase Herculase II Fusion (Agilent Technologies) and PCR conditions were: initial denaturation at 98°C, 2 min.; 30 cycles of 98°C, 30 s, 58°C, 30 s and 72°C, 30 s (3 min for *orf37*) and a final extension at 72°C, 3 min. The final plasmids were introduced into their respective strains by intergeneric conjugation. All oligonucleotides used are listed in Supplementary Table 12.

### Analysis of Metabolites by UPLC and Dereplication

Whole cultures (1 mL) of selected strains were extracted at three different times (3, 5, and 7 days of culture) and with three different organic solvents [ethyl acetate, ethyl acetate containing formic acid (1%) or butanol] and analyzed by reversed phase chromatography in an Acquity UPLC instrument fitted with a BEHC18 column (1.7 μm, 2.1 mm × 100 mm, Waters), with acetonitrile and MQ water + 0.1% TFA as solvents. Samples were eluted with acetonitrile (10%) for 1 min, followed by a linear gradient of acetonitrile (10–100%) over 7 min (flow rate of 0.5 mL/min; column temperature 35°C). For HPLC-MS analysis, an Alliance chromatographic system coupled to a ZQ4000 mass spectrometer and a SunFire C18 column (3.5 μm, 2.1 mm × 150 mm, Waters) was used with the above solvents. Elution was performed with an initial isocratic hold with acetonitrile (10%) for 4 min followed by a linear gradient of acetonitrile (10–88%) over 30 min (0.25 mL/min). MS analyses were done by ESI in the positive mode (capillary voltage 3 kV, cone voltage 20 V). Detection and spectral characterization of peaks were performed by photodiode array detection with Empower software (Waters).

### Isolation of Compounds from CS149 and CS057

Products **11** and **12** were isolated from two-liter culture of CS149 in R5A medium (40 mL × 50 mL, in 250 mL flasks). After the mycelium was removed by centrifugation, supernatants were filtered and then a pass-through cleanup strategy was used on a C18 cartridge (10 g, Waters) to eliminate non-desirable compounds. The sample was extracted with twice volume of ethyl acetate, concentrated under vacuo, resuspended in 1.5 mL 50:50 DMSO:MeOH and subsequently subjected to semipreparative HPLC using an Atlantis dC18 column (10 μm, 10 mm × 150 mm, Waters) and MQ water + 0.05% TFA/ACN (55:45) as a mobile phase at 5 mL/min.

Products **22** and **24** were isolated from 5 mL × 400 mL (in 2 L flasks) cultures of CS057 in R5A medium whose supernatants were first filtered, then concentrated on a C18 cartridge (10 g, Waters), and subsequently fractioned on a 0.1% TFA–MeOH gradient. Fractions containing desired compounds were dried *in vacuo*, resuspended in 1mL of 50:50 DMSO:MeOH, and processed on a preparative HPLC using a SunFire C18 column (10 μm, 10 mm × 250 mm, Waters) and an isocratic mixture 60:40 MQ water:ACN at 5 mL/min.

The purity of the isolated peaks was determined by HPLC–MS before structural elucidation. The isolated compounds were then dried *in vacuo*, resuspended in 50:50 tert-butanol:water and lyophilised. Yield and productivity of each compound was as follows: **11**, 1.1 mg (0.55 μg/mL); **12**, 0.5 mg (0.25 μg/mL); **22**, 2.1 mg (1.05 μg/mL); and **24**, 5.8 mg (2.9 μg/mL).

### Structural Elucidation of New Compounds

The new compounds sipanmycin A (**11**), sipanmycin B (**12**), warkmycin CS1 (**22**) and warkmycin CS2 (**24**) were analyzed by LC-DAD-ESI-TOF to obtain their UV-vis (DAD) spectra and determine their molecular formula based on the experimental accurate masses and the corresponding isotopic distribution. The structural elucidation of these compounds was carried out by detailed analysis of 1D (^1^H) and 2D (COSY, TOCSY, NOESY, HSQC and HMBC) NMR spectra further assisted by comparison with the spectral data reported for incednine (Futamura et al., 2008), silvalactam (Schultz et al., 2012), warkmycin and 4-*O*-deacetyl-warkmycin (Helaly et al., 2013). Solvents used in the NMR analyses were deuterated methanol (CD_3_OD) for compounds **11** and **12**, or deuterated chloroform (CDCl_3_) for compounds **22** and **24.** Relative configurations were determined by coupling constants and NOEs analyses. The molecular model of **11** was generated in Chem3D starting from the reported model for incednine (Futamura et al., 2008).

### LC-DAD-ESI-TOF and NMR Analyses

LC-DAD-ESI-TOF analyses were performed using an Agilent 1200RR HPLC equipped with a SB-C8 column (2.1 mm × 30 mm, Zorbax) coupled to a Bruker maXis Spectrometer. Solvent A consisted of 10% acetonitrile and 90% water with 1.3 mM trifluoroacetic acid (TFA) and ammonium formate, and solvent B was 90% acetonitrile and 10% water with 1.3 mM TFA and ammonium formate. The gradient started at 10% B and went to 100% B in 6 min, kept at 100% B for 2 min and returned to 10% B for 2 min to initialize the system. The mass spectrometer was operated in positive ESI mode. The instrumental parameters were: 4 kV capillary voltage, drying gas flow of 11 L/min at 200°C, nebulizer pressure at 2.8 bars. TFA-Na cluster ions were used for mass calibration of the instrument prior to sample injection. Pre-run calibration was by infusion with the same TFA-Na calibrant.

NMR spectra were recorded at 24°C on a Bruker AVANCE III-500 MHz (500 and 125 MHz for ^1^H and ^13^C NMR, respectively) equipped with a 1.7 mm TCI MicroCryoProbe^TM^, using the residual solvent signal as internal reference. Structural features were compared using the Dictionary of Natural Products (DPN) NMR Features database created by Prof. John Blunt, as described in Pérez-Victoria et al. (2016).

## Results

### PCR Analysis of a Collection of Actinomycetes Searching for Deoxy Sugar Biosynthesis Genes

In the context of our study, a collection of seventy one *Streptomyces* strains were screened for the presence of genes potentially involved in the biosynthesis of 6DOH. Total DNA from these strains was extracted and purified, and the presence of 6DOH biosynthesis genes determined by PCR analysis using different primers. On one hand, two primers located one within conserved regions in GSs (forward primer) and a second one (reverse primer) located within conserved regions for NDP-*D*-glycose-4,6-dehydratases (primers “GS-DH”). These two genes are usually (but not exclusively) adjacent in the chromosome and they are common to the biosynthesis of all 6DOH. The second pair of primers corresponded to internal and conserved regions for NDP-*D*-glucose-2,3-dehydratases, enzymes catalyzing the 2,3-dehydration step in the biosynthesis of 2,6-DOH (primers “2,3DH”). PCR analysis of the strains showed that forty nine strains gave positive amplicons with the “GS-DH” primers and fifty-two strains with the “2,3DH” primers, with a total of fifty-five strains being positive in at least one of the assays. The presence of the expected DNA sequence was verified by sequencing each amplicon.

### Generation of Mutants by Gene Disruption

The amplicons of the fifty five strains giving positive amplification with any of the two pairs of oligo primers were subcloned as EcoRI-HindIII fragments in pOJ260, an *E. coli* vector unable of replication in *Streptomyces.* The resultant constructs were introduced in the corresponding strains from which they were originated by intergeneric conjugation *E. coli-Streptomyces* and transconjugants selected for resistance to apramycin (resistance marker in the vector). Because pOJ260 is a suicide vector in *Streptomyces* spp., these transconjugants must be the consequence of an integration of the construct in the chromosome by homologous recombination through the insert in the vector. After several conjugation experiments, transconjugants were obtained in ten of the fifty-five strains tested. The lack of transconjugants in the remaining strains could be due to low efficiency of transconjugation or recombination, presence of DNA restriction systems, etc., but this was not further studied. Four mutants were obtained using the corresponding “GS-DH inserts” in the host strains CS65a, CS90a, CS149, and CS227, and seven mutants using the “2,3DH inserts” in the host strains CS57, CS92, CS113, CS123, CS147, CS149, and CS159. In one case, strain CS149, mutants were obtained with both “GS-DH” and “2,3DH” inserts.

### UPLC Comparative Analyses of Secondary Metabolites Production

**FIGURE 1:**
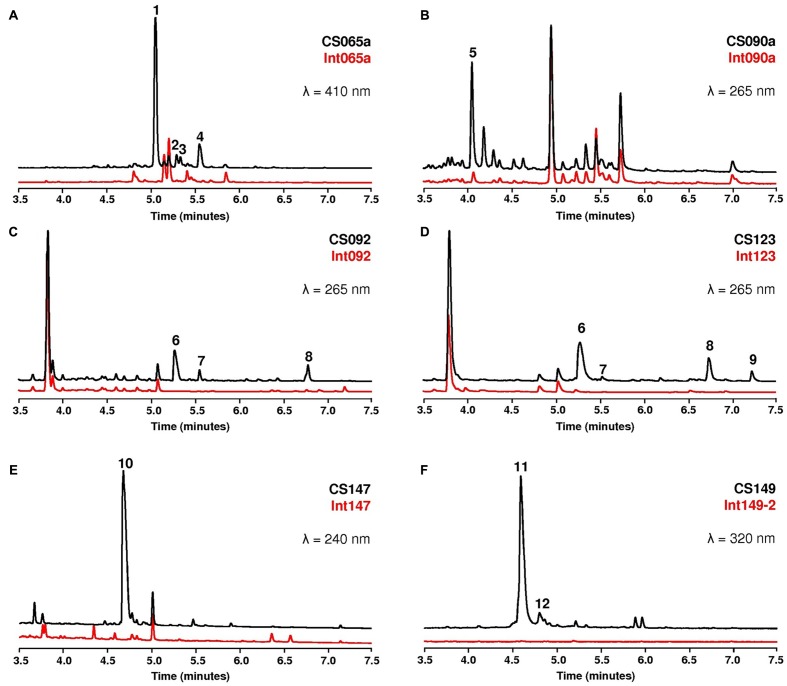
UPLC comparative analyses of the wild type strains and the different mutants. The numbers indicate differential peaks. Chromatograms of the wild type strain are in black and those of the mutants in red. The wavelength at which each chromatogram was extracted is indicated in each panel.

In order to determine if inactivation of the selected 6DOH biosynthesis genes gave rise to non-producing mutants and thus to the identification of the produced compounds, the ten strains and their corresponding mutants were grown on R5A liquid medium. Cultures were extracted with three different solvent systems (ethyl acetate, ethyl acetate plus 1% formic acid or butanol) at 3, 5, and 7 days and the metabolic profiles analyzed by UPLC. The different strains fall down in two different groups. The first group included strains showing some differential peaks, present in the wild type and absent in the mutant; this was the case for six strains: CS065a, CS090a, CS92, CS123, CS147, and CS149 (**Figures 1A–F**). The second group included strains not showing differential peaks between the wild type and the mutant strains in samples extracted with any of the solvents described above or at any time tested; this was the case for four strains: CS057, CS113, CS159, and CS227.

Extracts of the six strains of the first group and their corresponding mutants were dereplicated by LC-UV-LRMS and LC-HRMS against our in-house database (Pérez-Victoria et al., 2016) and the Chapman & Hall Dictionary of Natural Products (Buckingham, 2013) trying to determine if the differential compounds were already known. In five out of the six strains, compounds were identified in the differential peaks as: chromomycins A3, Ap and A2 (peaks **1**, **2** and **4** in strain CS065a), benzanthrins A and B (both under the same peak **5** in strain CS090a), lobophorin A and B (peaks **6** and **8** in strains CS092 and CS123) and vicenistatin (peak **10** in strain CS147) (**Figures 1A–E**). In some cases, small minor peaks with characteristic spectrum of the family were also detected and were not further characterized (peak **3** from CS065a, **7** from CS092, and **7** and **9** from CS123). All these identified compounds contain 6DOH attached to their respective aglyca and therefore it can be assumed that the generated mutants were non-producer of the corresponding compounds.

### Identification of Two Novel Macrolactams Produced by Strain CS149

**FIGURE 2:**
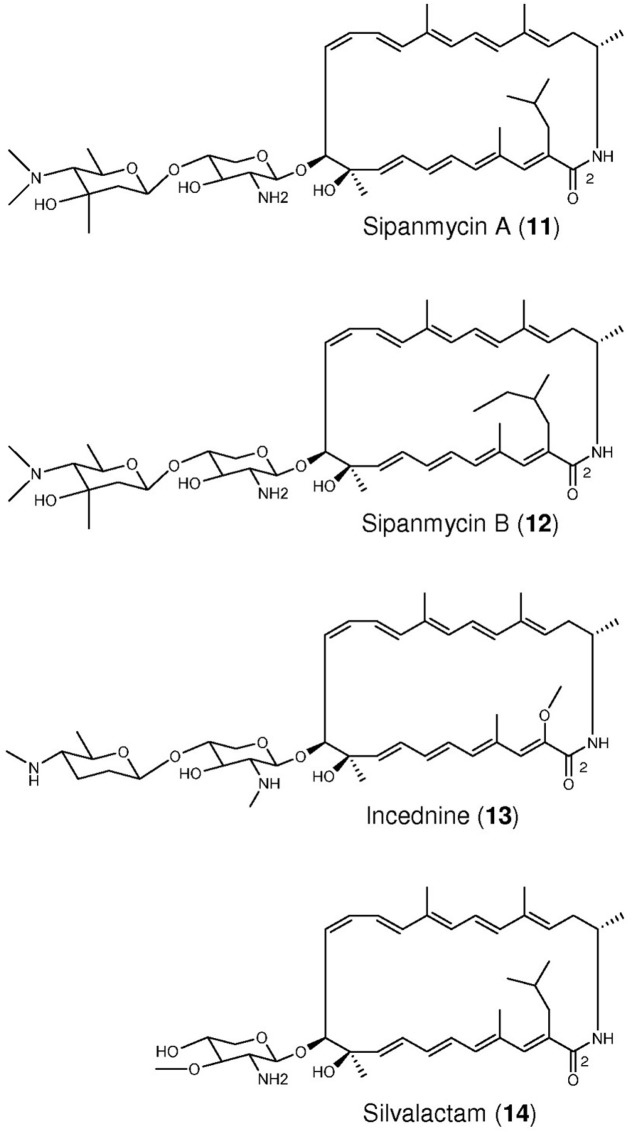
Chemical structures of members of the macrolactam family. The two novel compounds, sipanmycins A and B, were isolated from peaks **11** and **12** (**Figure 1F**).

The sixth strain (CS149) showing differential peaks that were not identified by dereplication was characterized in more detail. Two different mutants were obtained from this strain in which the glucose synthase and 4,6-dehydratase genes (mutant int149-1) or the 2,3-dehydratase gene were inactivated (mutant int149-2). Comparative analysis of the UPLC profiles of wild type strain CS149 and both mutants (**Figure 1F**) showed two differential peaks, **11** and **12**, absent in the mutant strain. From these peaks, two compounds were isolated, purified and their structures elucidated. These compounds, designated sipanmycin A (**11**) and B (**12**), belong to a family of 24-membered macrolactams that include incednine (**13**) (Futamura et al., 2008) and silvalactam (**14**) (Schultz et al., 2012) (**Figure 2**).

The molecular formula of **11** was established as C_46_H_71_N_3_O_8_ based on the observed ion [M+Na]^+^ at 816.5143 (calcd. for C_46_H_71_N_3_O_8_Na+ = 816.5133, Δ*m* = 1.2 ppm) (Supplementary Figure 1). Its UV (DAD) spectrum (Supplementary Figure 2) shows a main maximum at 320 nm with a secondary maximum (shoulder) at 360 nm, suggesting the presence of a polyene moiety. Complete NMR spectral data including ^1^H, COSY, TOCSY, NOESY, HSQC, and HMBC spectra were acquired to determine the structure (Supplementary Figures 3–9). Although the ^1^H spectrum showed broad signals, in the HSQC spectrum fourteen sp^2^ methines could be identified which, according to the COSY and TOCSY spectra, would belong to two independent spin systems. This structural feature agrees with the conjugated polyene motif compatible with the UV (DAD) spectrum. On the other hand, two anomeric methine groups from two different spin systems were likewise deduced from the ^1^H, COSY, TOCSY and HSQC spectra, suggesting the presence of two monosaccharide (aldose type) units. After searching the DPN NMR Features database (Pérez-Victoria et al., 2016), these structural features are found in incednine, a glycosylated 24-membered macrolactam natural product isolated from the culture broth of *Streptomyces* sp. ML694-90F3 (Schultz et al., 2012). Direct comparison of the NMR spectra of **11** with the corresponding data reported for incednine confirmed the structural similarity of both compounds. Thorough analysis of the 2D NMR spectra assisted by comparisons with the NMR data of incednine allowed stablishing the connectivity of 1 (Supplementary Figure 10). The 24-membered macrolactam ring of **11** is identical to that of incednine differing just in the side chain substituent at C-2, which for **11** is an isobutyl chain instead of a methoxy group. Interestingly, the same macrolactam ring aglycon is found in silvalactam (Schultz et al., 2012), another closely related antibiotic which bears just one monosaccharide. The pentopyranose (2-deoxy-2-amino-β-D-xylopyranose) directly attached to the aglycon of **11** is likewise almost identical to that found in incednine just lacking the *N*-methylation. However, the second monosaccharide unit (at the non-reducing end of the disaccharide moiety) shows important differences. Compared to the corresponding one in incednine it displays *N*-dimethylation rather than monomethylation and it is not a deoxy sugar at C-3″ carrying at this position both a hydroxyl and a methyl group. The relative configuration of the monosaccharides in **11** was determined based on coupling constants and NOEs analysis (Supplementary Figure 11). The absolute stereochemistry of the chiral centers in **11** was assumed to be identical to that reported for incednine (Futamura et al., 2008; Ohtani et al., 2010) based on the key NOEs observed for **11** which turned out to be identical to those reported for incednine. Molecular modeling of **11** based on the reported models for incednine and incednam (its aglycon) (Pérez-Victoria et al., 2016), perfectly match these key NOEs and confirms that the aglycon of **11** and incednam share the same absolute stereochemistry (Supplementary Figure 12). In a similar fashion, modeling also shows a perfect match with the key NOEs involving the monosaccharides confirming the D-configuration of both sugar units (Supplementary Figures 13, 14). The monosaccharide glycosylating the macrolactam must share the same biosynthetic pathway in incednine and **11** since they just differ in an *N*-methylation. On the other hand, the non-reducing monosaccharide in incednine is identical to D-forosamine carrying just *N*-monomethylation instead of *N*-dimethylation. It is reasonable to assume that the non-reducing monosaccharide in **11** is being biosynthesized from D-glucose via a route related to that of forosamine (Zhao et al., 2005; Hong et al., 2008).

The molecular formula of **12** was established as C_47_H_73_N_3_O_8_ based on the observed ion [M+H]^+^ at 808.5475 (calcd. for C_47_H_74_N_3_O^8+^ = 808.5470, Δ*m* = 0.6 ppm) (Supplementary Figure 15). This molecular formula contains one extra C atom and two extra H atoms compared to **11**. The UV (DAD) spectrum (Supplementary Figure 16) was identical to that of **11**. Likewise the complete NMR spectral set (Supplementary Figures 17–22) showed just minor differences compared to **11**. The structural relationship of **11** and **12** was obvious and the difference was easily located, after analysis of the 2D NMR spectra, in the side chain at C-2 being in this case a 2-methyl-1-butyl group (Supplementary Figures 23, 24). The coincidence in chemical shifts of **11** and **12** (Supplementary Tables 1, 2) indicates they share the same absolute configuration. The absolute configuration of the extra chiral center in 2 (C-30) could not be determined though.

### Awakening of the Expression of Silent Clusters

The four strains that did not show differential peaks by comparison of the UPLC metabolic profiles (strains CS057, CS113, CS159, and CS227) were further characterized in an attempt to identify the BGC (if any) where the mutant was generated and subsequently to identify the corresponding compound. With this aim, we used two different strategies trying to activate the expression of the corresponding silent clusters: a nutritional and a genetic approach.

**FIGURE 3:**
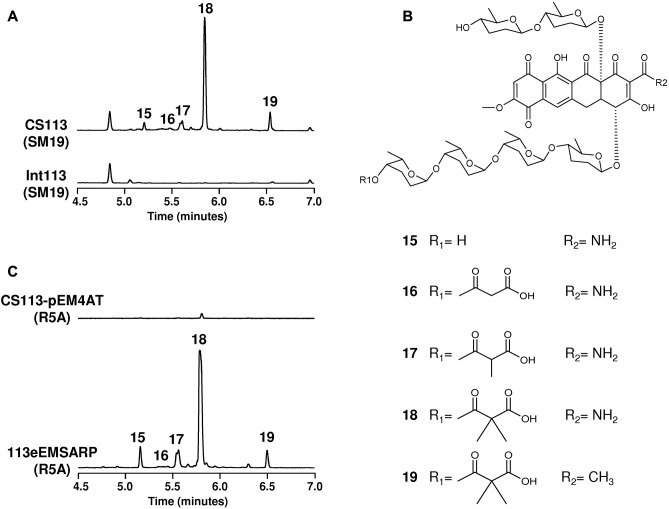
Activation of the expression of cluster 23 in strain CS113. **(A)** UPLC comparative analysis of strain CS113 and mutant int113 grown on SM19 medium. **(B)** Chemical structures of cervimycins (compounds from peaks 15 to 19). **(C)** UPLC comparative analysis of strain CS113 harboring vector pEM4AT or pEMSARP both grown on R5A medium.

In the nutritional approach four different media (SM, SM10, SM17, and SM19) differing in pH and in the carbon and nitrogen sources were used. The strains were grown in these media for several days and, after extraction with three different solvent systems, the UPLC profiles were compared between the wild type and the mutants. This kind of strategy has proved to be successful in a wide range of microorganisms (Bode et al., 2002). In the case of three strains (CS057, CS159, and CS227) there were no significant differences suggesting that none of the possible BGCs for secondary metabolites was activated by changing the nutritional conditions. Consequently, it was not possible to identify the compound coded by the mutated cluster. However, UPLC profiles comparison between strain CS113 and int113 mutant showed several differential peaks when they were grown on SM19 medium (**Figure 3A**). UPLC analysis and dereplication of the compounds present in five peaks against our in-house database (Pérez-Victoria et al., 2016), and the Chapman & Hall Dictionary of Natural Products Compounds (Buckingham, 2013) revealed that, based on the absorption spectrum and mass analysis, they contain compounds compatible with members of the cervimycins family (**Figure 3B**), a tetracycline-type compound with six 6DOHs attached. They were identified as antibiotic HKI 10311129 (**15,** *m/z* = 1113.4765 [M+H]^+^), antibiotic A2121-1 (**16,** *m/z* = 1199.4794 [M+H]^+^), cervimycin D (**17,** *m/z* = 1213.4939 [M+H]^+^), cervimycin C (**18**, *m/z* = 1227.5115 [M+H]^+^) and cervimycin A (**19**, *m/z* = 1226.5152 [M+H]^+^) (Herold et al., 2004, 2005).

**FIGURE 4:**
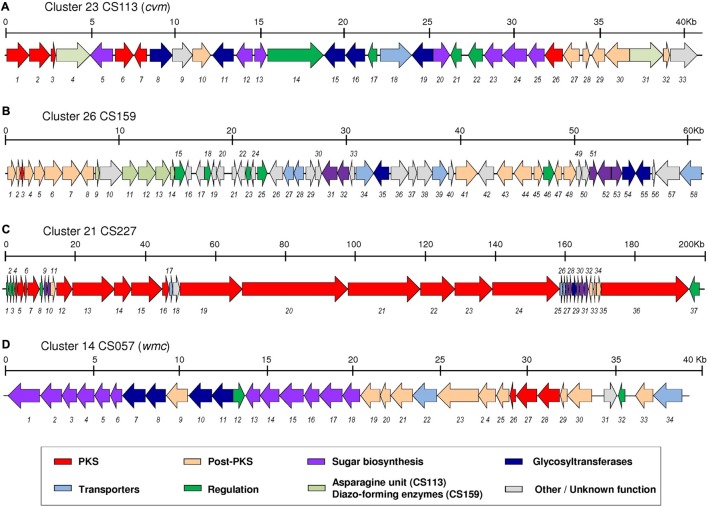
Genetic organization of several clusters identified in this study. **(A)** Cluster 23 from CS113 (*cvm*). **(B)** Cluster 25 from CS159. **(C)** Cluster 21 from CS227. **(D)** Cluster 14 from CS057 (*wmc*). Generic functions for the gene products are indicated using colored code.

In the second approach we applied two different genetic strategies that require the previous knowledge of the DNA sequence and genetic organization of the targeted cluster. For this approach, the genomes of the four strains were sequenced and searched for the presence of putative BGCs using the bioinformatic tool “antibiotics and Secondary Metabolite Analysis Shell” (antiSMASH v.4) (Blin et al., 2017). All strains showed the presence of a large number of BCGs (between 20 and 33) with only one glycosylated BGC in strains CS113 (cluster 23, Supplementary Table 3), CS159 (cluster 26, Supplementary Table 4) and CS227 (cluster 21, Supplementary Table 5), and two glycosylated BCGs in strain CS057 (clusters 14 and 19, Supplementary Table 6). Sequence analysis allowed us to confirm that the mutants in strains CS113, CS159, and CS227 were effectively generated in the corresponding glycosylated BGC and, in the case of CS057, at cluster 14. The genetic organization of these four clusters is shown in **Figure 4**. All four clusters seemed to contain at least one putative pathway-specific positive regulatory gene for the putative control of secondary metabolite biosynthesis. In order to attempt the activation of the expression of these clusters we used two approaches: (i) inserting a strong and constitutive promotor (*ermE^∗^p*) in the chromosome to control the expression of the putative regulatory gene or (ii) ectopic expression of the regulatory gene under the control of *ermE^∗^p* in the wild type strain either using a replicative (pEM4AT) or an integrative (pSET152e) vector.

#### Cluster 23 in Strain CS113

This 40 kb BGC would code for a type II polyketide that could contain at least five 6DOH based on the presence of five glycosyltransferase genes (**Figure 4A** and Supplementary Table 7). AntiSMASH analysis revealed that 41 and 39% of the genes belonging to this cluster show similarity with genes of the already described polyketomycin (Daum et al., 2009) and dutomycin (Sun et al., 2016) clusters, respectively, the main difference being the presence of two genes putatively involved in the biosynthesis of an “asparagine unit” in cluster 23 (*cvm4* and *cvm31* in **Figure 4A**). The cluster contains a putative positive regulatory gene (*cvm22*, Supplementary Table 7) belonging to the SARP family. Upon expression of this gene in the replicative and the integrative plasmids under the control of the *ermE^∗^p* or by inserting this promotor upfront of the *cvm22* gene, production of five cervimycins (**15**–**19**) was observed, being absent in the wild type strain only containing the vector (**Figure 3C**). Therefore, we can conclude that cluster 23 is not expressed in the wild type strain under our standard cultivation conditions (i.e., R5A medium) but its awakening is possible by manipulating the expression of the regulatory gene *cvm22*. To our knowledge, the *cvm* cluster involved in the biosynthesis of cervimycins in *Streptomyces tendae* HKI-179 has not been previously described, being only the nucleotide sequence of *cerJ* (ketosynthase) available at GenBank (accession number JN001183) (Bretschneider et al., 2011).

#### Cluster 26 in Strain CS159

This cluster also could code for a compound with at least three 6DOH attached to the aglycon since there are three glycosyltransferase genes (**Figure 4B** and Supplementary Table 8). It could be a type II polyketide based on the presence of genes coding for an acyl carrier protein (*orf3*), a ketoacyl reductase (*orf4*) and two cyclases (*orf2* and *orf5*), but genes encoding for beta-ketoacyl synthases (KS) are missing. Hypothetically, the lack of these genes could be overcome by the presence of two orphan KSs in the vicinity of cluster 26, in particular at cluster 24 (Supplementary Table 4). By searching in databases we found the closest similar cluster (44% identity) being that for the biosynthesis of lomaiviticin from marine actinomycetes *Salinispora pacifica* DPJ-0019 (Woo et al., 2012) and *Salinispora tropica* CNB-440 (Kersten et al., 2013a). Among the 61.5 kb of cluster 26 there are six structural genes (and other three with unassigned function) without any ortholog at the lomaiviticin gene cluster that let us hypothesize the compound coded by this cluster may be related but different from lomaiviticin. Interestingly, most of the genes contained in cluster 26 have an ortholog in the same region of the genome of *Streptomyces* sp. NRRL WC-3753 thus indicating that this cluster may be present in both strains (Supplementary Table 8).

Based on the absorption spectrum of lovaimiticin, we searched by UPLC for the presence of compounds with a similar spectrum but we were unsuccessful. The cluster contains a potential positive regulator of the OmpR family (*orf15*), a type of regulator that had been proven as an activator of the production of several secondary metabolites such as nikkomycin (Liu et al., 2005) and jadomycin B (Wang et al., 2009). Attempts to activate the expression of this gene and the subsequent production of the targeted compound were also unsuccessful. As a conclusion, the compound coded by this cluster remains unknown.

#### Cluster 21 in Strain CS227

This cluster is one of the longest BGC described until now in *Streptomyces* (195 kb) and it might be responsible for the production of a large type I polyketide with at least one sugar attached to the aglycon as suggested by the presence of one glycosyltransferase gene (**Figure 4C** and Supplementary Table 9). Searching in databases showed that 68% of the genes in this cluster presented similarity with genes from the 51-membered macrolide stambomycin BCG (Laureti et al., 2011). In fact, all the PKSs encoded by cluster 21 of CS227 (except Orf20 and Orf21) and those of the stambomycin cluster of *Streptomyces ambofaciens* ATCC23877 are very similar both in amino acid sequence (percentage of identity ranging between 55 and 80; Supplementary Table 9) and domain architecture (they share the same predicted modules and domains) The main differences concerning PKSs of both clusters relay in PKS Orf20 that shares the first two modules with SAMR466 but has four additional modules and in PKS Orf21, composed by four modules, that has not an ortholog in the stambomycin biosynthesis cluster. Furthermore, acyltransferase domain analysis using antiSMASH and PRISM software (Skinnider et al., 2016) predicts that 23 × malonyl-CoA, 9 × methylmalonyl-CoA and one ethylmalonyl-CoA units might participate in the biosynthesis of the final compound in CS227, whilst in stambomycin biosynthesis the PKSs introduce 16 × malonyl-CoA, 8 × methylmalonyl-CoA and one unusual alkylmalonyl-CoA unit (Laureti et al., 2011; Ray et al., 2016). In addition, the proposed functions for the enzymes involved in the biosynthesis of the sugar attached to the polyketide deduced from cluster 21 of CS227 indicates that it might be a 3-amino-2,6-DOH, in contrast to the 3-amino-6-DOH β-mycaminose of stambomycins.

Stambomycins are not produced by *S. ambofaciens* under normal laboratory growth conditions. However, the constitutive expression of a regulatory gene of the LuxR family located within the cluster triggered the expression of the biosynthetic genes leading to the identification of four stambomycins (Laureti et al., 2011). In our cluster, we found several potential positive regulatory genes belonging to the SARP (*orf2* and *orf8*) and LuxR family (*orf37*) (**Figure 4C**). Giving the similarity between both clusters we attempted to activate cluster 21 by independent expression of both types of transcriptional activator genes (*orf2* and *orf37*) in a replicative or an integrative vector and also by inserting the *ermE^∗^p* in the chromosome upfront each of these genes. In all the cases, no differential peaks were found. Therefore, we could not identify the compound encoded by the analyzed cluster.

### Cluster 14 in Strain CS057

**FIGURE 5:**
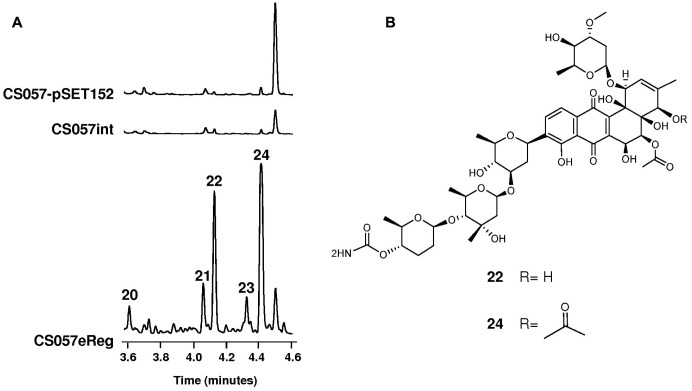
Activation of the expression of cluster 14 in strain CS057. **(A)** UPLC comparative analysis of strain CS057 harboring vector pSET152, mutant int057 and strain CS057 harboring pSET57reg (expressing *wmc32*) grown on R5A medium. **(B)** Chemical structures of warkmycins CS1 (**22**) and CS2 (**24**).

This 38 kb BGC could code for a type II polyketide (**Figure 4D** and Supplementary Table 10). AntiSMASH analysis showed 68% of genes in this cluster presenting similarity with genes involved in the biosynthesis of the angucycline landomycin. There are four glycosyltransferase genes at this cluster that suggested the presence of a minimum of four 6DOH in the final compound. Cluster 14 also contains a possible transcriptional activator gene of the OmpR-family (*wmc32*). By using both genetic strategies mentioned above, we found five differential peaks (**20**–**24**) between the experimental strains and the corresponding controls, all of them sharing the same absorption spectrum (**Figure 5A**). Dereplication of the compounds in these peaks against our in-house database (Pérez-Victoria et al., 2016) and the Chapman & Hall Dictionary of Natural Products (Buckingham, 2013) showed that they could be related to the glycosylated angucycline warkmycin (Helaly et al., 2013). To confirm the novelty of these compounds, two of them (**22,** *m/z* = 1025.4341 [M+NH_4_]^+^; and **24**, *m/z* = 1049.4104 [M+H]^+^) were purified and their structures elucidated and named warkmycin CS1 and CS2, respectively (**Figure 5B**).

Compound **22** showed a UV (DAD) spectrum (Supplementary Figure 25) with a maximum at 220 nm identical to that reported for warkmycin. Its molecular formula was established as C_48_H_65_NO_22_ based on the observed ion [M+NH4]^+^ at 1025.4341 (calcd. for C_48_H_69_N_2_O_22_^+^ = 1025.4336, Δ*m* = 0.5 ppm) (Supplementary Figures 26, 27). The molecular formula matches that of 4-*O*-deacetyl-warkmycin. However, this known warkmycin could not explain the fragments observed in-source in the HRMS experiment. Thus, a complete set of NMR spectra including ^1^H, COSY, TOCSY, NOESY, HSQC and HMBC spectra (Supplementary Figures 29–34) was acquired to ensure the novelty of **22** and determine its structure. The analysis of the NMR data confirmed **22** as a novel warkmycin displaying for most signals almost identical chemical shift compared with 4-*O*-deacetyl-warkmycin. The main differences were observed for the resonances of sugar rings A and D. Such difference turned out to be a consequence of the different position of the carbamoyl functional group which is located at position 4 of the sugar ring D in **22** while in 4-*O*-deacetyl-warkmycin is placed at position 4 of the sugar ring A. The key HMBC observed between the proton at position C-4D and the carbonyl carbon of the carbamoyl functionality unequivocally distinguishes **22** from its regioisomer (4-*O*-deacetyl-warkmycin) (Supplementary Figure 35). The mentioned in-source fragments observed in its HRMS spectrum likewise unequivocally located the carbamoyl functionality at sugar ring D (Supplementary Figure 28).

The UV spectrum of compound **24** (Supplementary Figure 36) was identical to **22**. Its molecular formula was established as C_50_H_67_NO_23_ based on the observed ion [M+NH4]^+^ at 1067.4450 (calcd. for C_50_H_71_N_2_O_23_^+^ = 1067.4442, Δ*m* = 0.9 ppm) (Supplementary Figure 37). The difference in its molecular formula compared with **22** immediately indicated that **24** bears an extra acetyl group. Once again **24** shares the same molecular formula as warkmycin but the analysis of the NMR spectral data (Supplementary Figures 38–45) and the in-source fragmentation observed in its HRMS spectrum unequivocally located the position of the carbamoyl in the same place found in **22**. Thus **24** is a regioisomer of warkmycin and is identical to **22** carrying an extra acyl group at C-4. The effect of the extra acylation is reflected in the corresponding shifting in resonance frequency for the nearby positions to C-4 (Supplementary Table 11).

The absolute stereochemistry of **22** and **24** is identical to that reported for the known warkmycins based on chemical shift comparisons and the observation of the same coupling constants pattern and key NOEs as those reported for warkmycin (Supplementary Figures 46, 47).

## Discussion

The search for novel bioactive natural products is a challenge for researchers in the field based on the need to combat emergent infectious diseases and resistant pathogens in the field of antibiotics or to find better chemotherapeutic drugs in the field of antitumor drugs (Newman and Cragg, 2016; Zhang et al., 2017a). Through classical screening programs a large number of bioactive compounds have been isolated and many of them arrived to clinical use (Katz and Baltz, 2016). However, these screenings have a low hit rate but a high rediscovery rate being necessary to isolate a large number of strains from different origins and to test many extracts in order to identify a novel compound (Zhang et al., 2017a). It is therefore an expensive, low productive and time-consuming work. Consequently, novel approaches must be developed to improve the chances of getting novel bioactive natural products. The continuously growing available information on the sequencing of genomes of microorganisms, and in particular those for actinomycetes, is revealing the large potential of these microorganisms to synthesize a enormous variety of compounds, many of which are not produced under standard laboratory cultivation conditions. Targeted screening in combination with genome mining can increase the chance of discovering novel compounds, as it is shown in this work were we have focused our efforts to identify novel glycosylated natural products. However, in many cases not even a combination of screening approaches can deal with the fact that some BGCs are silenced under the laboratory conditions used. Access to this potential reservoir of bioactive molecules can be only achieved at present using recombinant DNA technology in combination with genome sequencing (Rutledge and Challis, 2015; Zarins-Tutt et al., 2016; Ren et al., 2017). In this work, we have used such an approach to target BGCs coding for compounds containing 6DOH in their structures. The reason to select these type of compounds resides in the fact that many therapeutically important antibiotics, antifungals, antiparasitics and anticancer drugs contain 6DOH attached to the aglycone core (Salas and Méndez, 2007).

In order to search for glycosylated BGCs in the genome of seventy-one streptomycete strains we choose several 6DOH genes as targets. On one hand, two genes very well conserved whose products catalyze the two earliest and common steps in the biosynthesis of 6DOH: a GS and a DH, thus conducting to the biosynthesis of 6DOH. In most of the BGCs so far characterized they are linked together in the chromosome and usually they are forming part of the BGC with a few exceptions, the most representative ones include genes involved in the biosynthesis of *L*-rhamnose which are not located inside the corresponding BGC (Madduri et al., 2001; Gullon et al., 2006; Ramos et al., 2008). The use of these two genes as probes is a quite efficient strategy since, based on the information emerging from the sequence of the genomes of many streptomycetes, they do not contain a large number of glycosylated BGCs per organism: on average between none and one glycosylated cluster can be found in each strain. The second targeted gene, coding for a NDP-*D*-glucose-2,3-dehydratase, participates in the biosynthesis of 2,6-DOH and therefore is a more selective probe. In our study, 77% of the strains tested gave positive results against at least one of the probes, 69 and 73% giving positive against the “GS-DH” and the “2,3DH” probes, respectively.

Insertional inactivation of a gene cluster by homologous recombination is a usual genetic tool to prove the involvement of a BGC in the production of a given compound (Olano et al., 2011). In our study, ten mutants were obtained affecting different clusters. Comparative analyses between the UPLC profiles of the wild type and the corresponding mutant was sufficient to detect changes in the profile of six strains and the identification by dereplication of the potential compounds coded by the targeted BGC in each mutant. This was a clear proof of concept: the strategy allows achieving the proposed objective, the identification of a BGC for a glycosylated compound. Some of these compounds were not further characterized since they corresponded to already known compounds. However, in the case of strain CS149 two novel macrolactams, sipanmycin A **(11)** and B **(12),** were isolated and purified. They are related to incednine and silvalactam, but mainly differing in the sugar pattern of the compounds. Incednine has been shown as a potent modulator of the anti-apoptotic function of Bcl-xL, thus having potential implications in cancer chemotherapy (Futamura et al., 2008).

UPLC profiles comparison was not sufficient to identify the produced compound by some of the clusters since no differential peaks were observed even after several incubation days and using different extraction systems. This might be due to the fact that some BGCs are not expressed under normal laboratory conditions and it is necessary to force the expression of the corresponding cluster. Using nutritional and genetic approaches (Bode et al., 2002; Rutledge and Challis, 2015), we have been successful in activating the expression of two “silent” BGCs in two different strains out of four strains tested. In strain CS113, detection of the production of members of the cervimycin family of antibiotics was achieved by changing the composition of the growth medium or by manipulating the expression of a positive regulator of the SARP family. In strain CS057 production of five members of the warkmycin family of antibiotic and cytotoxic compounds, two of them novel members, arose as a consequence of the manipulation of the expression of a transcriptional activator gene of the OmpR-family.

Biosynthesis of different compounds by actinomycetes may confer the producer strains with competitive advantages in the environment where they live. In the case of the strains in this study, isolated from the integuments of ants, it could be also an evolutionary advantage for the ants to be protected from pathogen microorganisms that could be a threat for their survival. Ants have been shown to be infected by different fungal pathogens (Yek et al., 2013) and different reports, in addition to the work we described here, pointed out to the identification of bioactive natural products from actinomycetes living in symbiosis with the ants (Seipke et al., 2012; Van Arnam et al., 2015, 2016).

## Conclusion

Sequencing of the genomes of actinomycetes has revealed a large potential for the biosynthesis of bioactive natural products in these organisms. Target-directed search for clusters coding for glycosylated bioactive compounds using a combination of different approaches has been proven as a good strategy in the search for novel compounds. Once the clusters are identified different approaches can be applied in order to activate the expression of “silent” or “low expressed” BGCs thus conducting to the identification and isolation of novel bioactive compounds.

## Author Contributions

JS, CM, and CO conceived and designed the project. MM, LG-M, RG, and CS conducted the experiments. JS and CO analyzed the data and drafted the manuscript. MM, AB, IP-V, JM, and FR purified the compounds and determine chemical structures. MM contributed to preparing the final version of the paper. All authors read and approved the final manuscript.

## Conflict of Interest Statement

The authors declare that the research was conducted in the absence of any commercial or financial relationships that could be construed as a potential conflict of interest.

## Footnotes

1 https://submit.ncbi.nlm.nih.gov/subs/wgs
