# Supplementary material for: Searching for Glycosylated Natural Products in Actinomycetes and Identification of Novel Macrolactams and Angucyclines
Source: Front Microbiol. 2018 Jan 30;9:39. doi: 10.3389/fmicb.2018.00039 (PMC5797532; doi:10.3389/fmicb.2018.00039)
Supplement: Supplementary file 1 [file Data_Sheet_1.pdf]

Supplementary Material

Searching for glycosylated natural products in actinomycetes isolated from leaf-cutting ants: activation of two silent clusters and identification of novel macrolactams and angucyclines

Mónica G. Malmierca[1,2], Lorena González-Montes[1], Ignacio Pérez-Victoria[3], Carlos Sialer[1], Alfredo F. Braña[1,2], Raúl García Salcedo[1], Jesús Martín[3], Fernando Reyes[3], Carmen Méndez[1,2], Carlos Olano[1,2] and José A. Salas[1,2*]

*** Correspondence:** José A. Salas; jasalas@uniovi.es

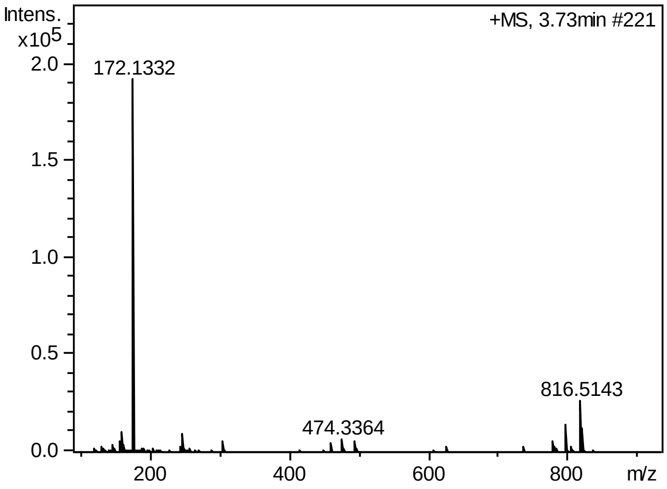

Supplementary Figure 1. HRMS spectrum of sipanmycin A (**11**).

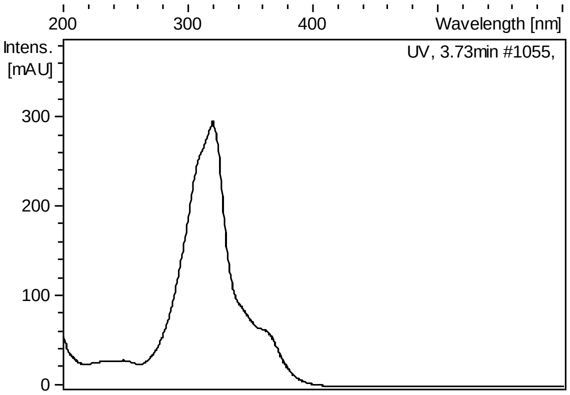

Supplementary Figure 2. UV-vis (DAD) spectrum of sipanmycin A (**11**).

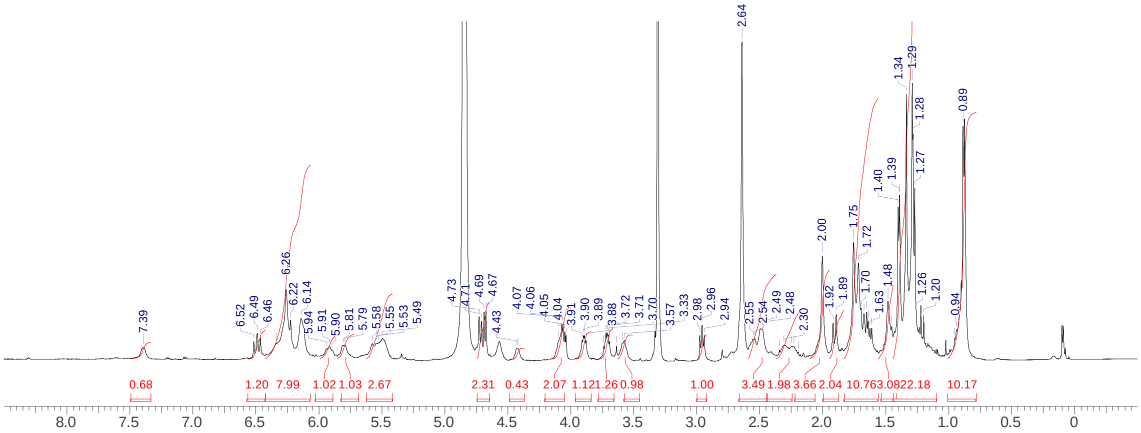

Supplementary Figure 3. ^{1}H NMR spectrum (CD_3OD, 500 MHz) of sipanmycin A (**11**).

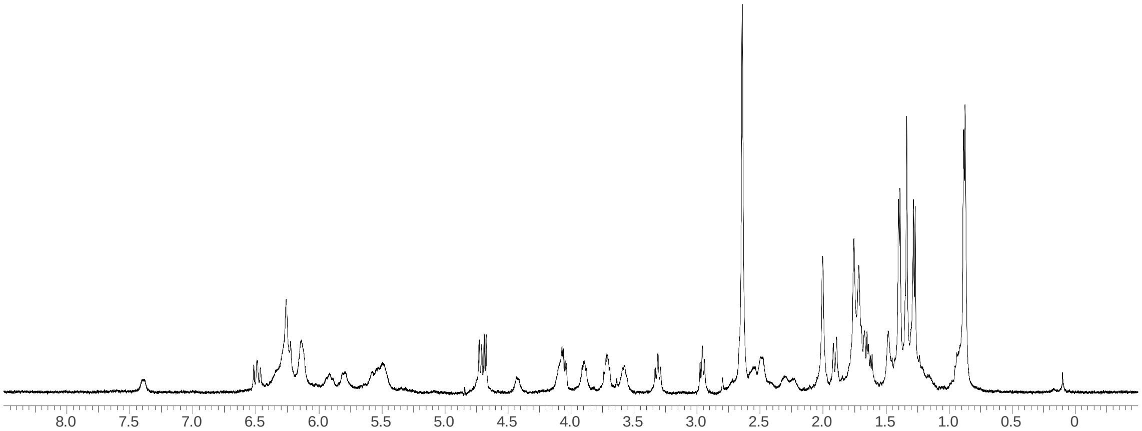

Supplementary Figure 4. Diffusion-filtered ^{1}H NMR spectrum of sipanmycin A (**11**).

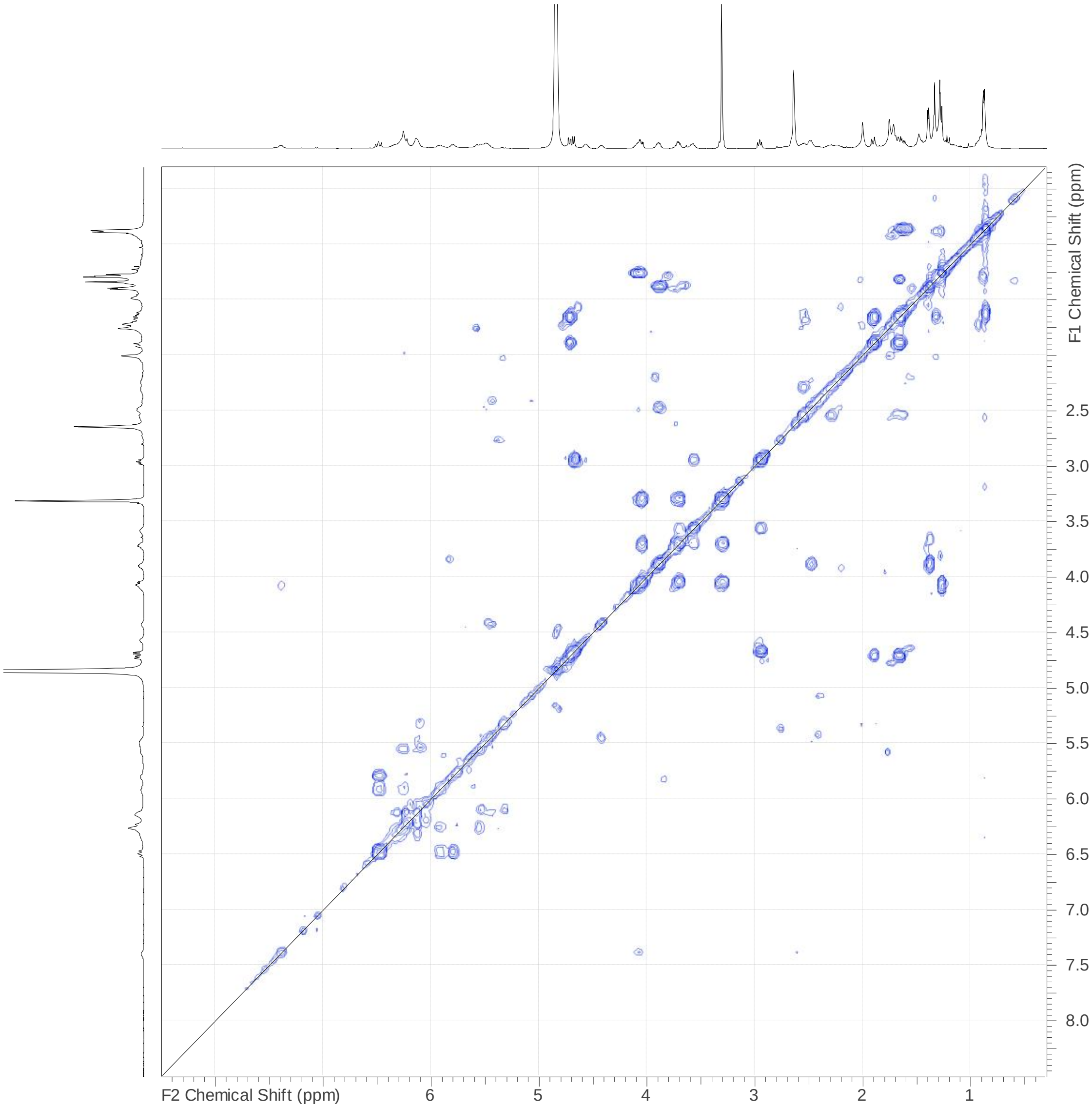

Supplementary Figure 5. COSY spectrum of sipanmycin A (**11**).

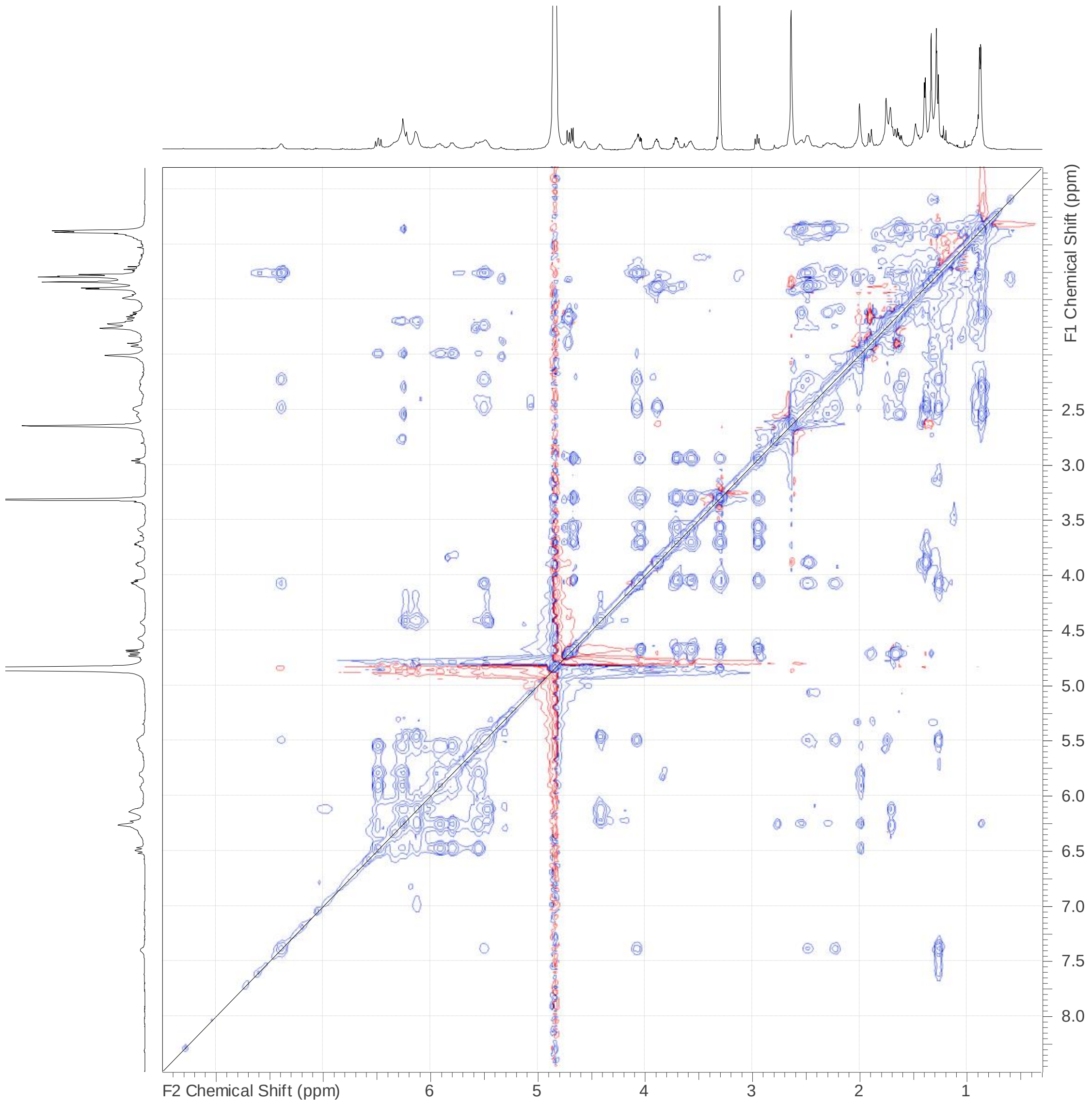

Supplementary Figure 6. TOCSY spectrum of sipanmycin A (**11**).

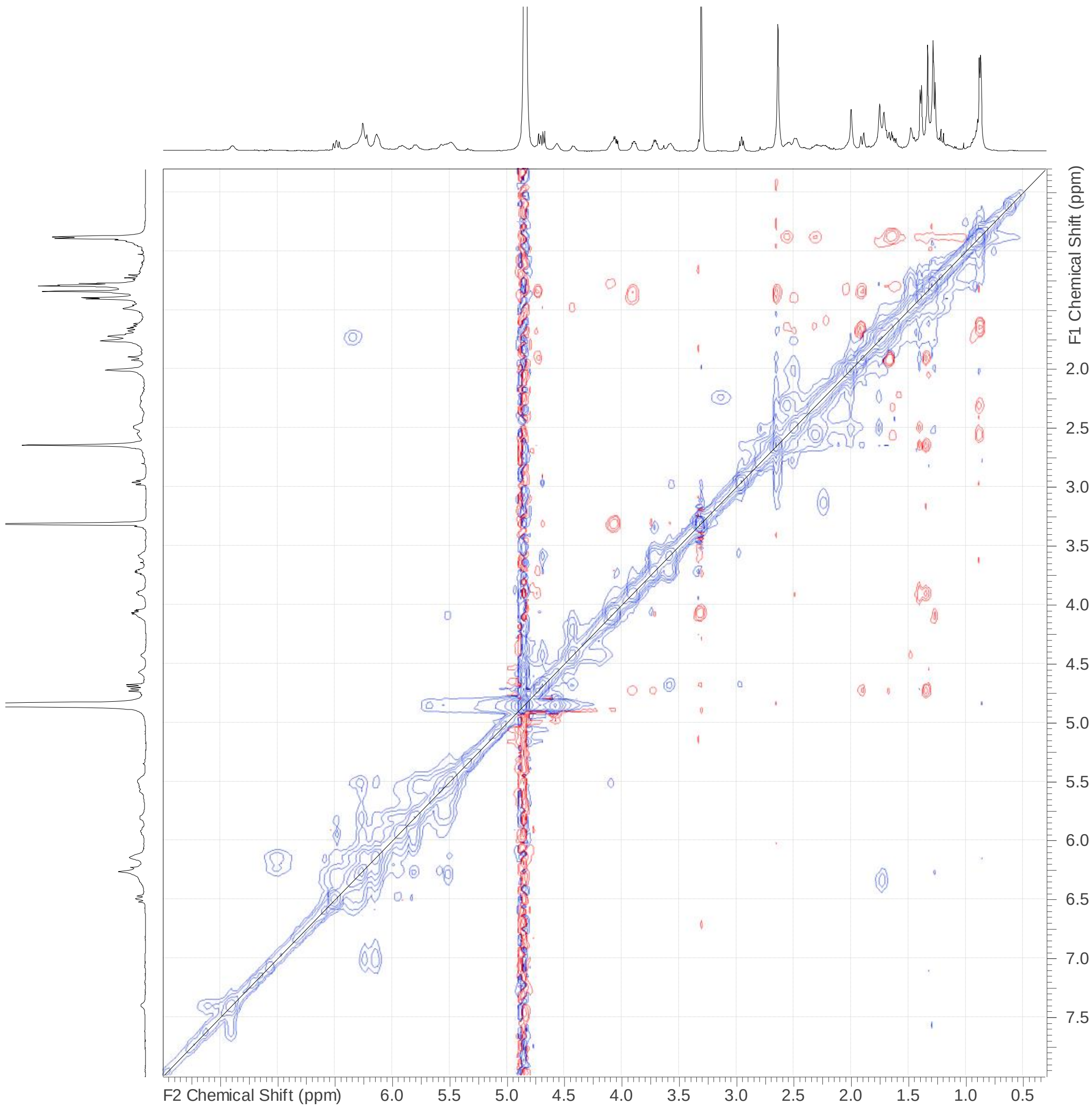

Supplementary Figure 7. NOESY spectrum of sipanmycin A (**11**).

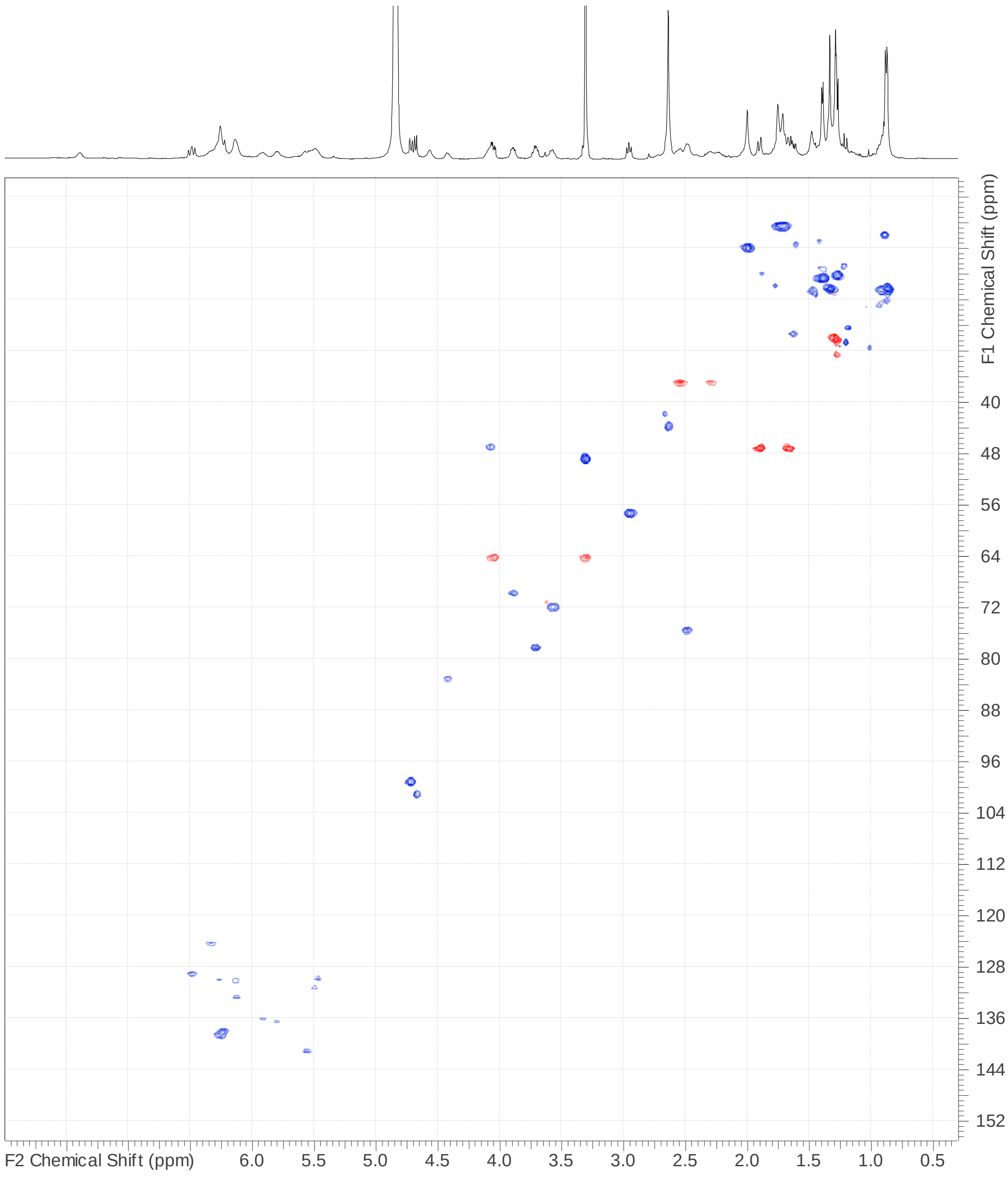

Supplementary Figure 8. Edited HSQC spectrum of sipanmycin A (**11**).

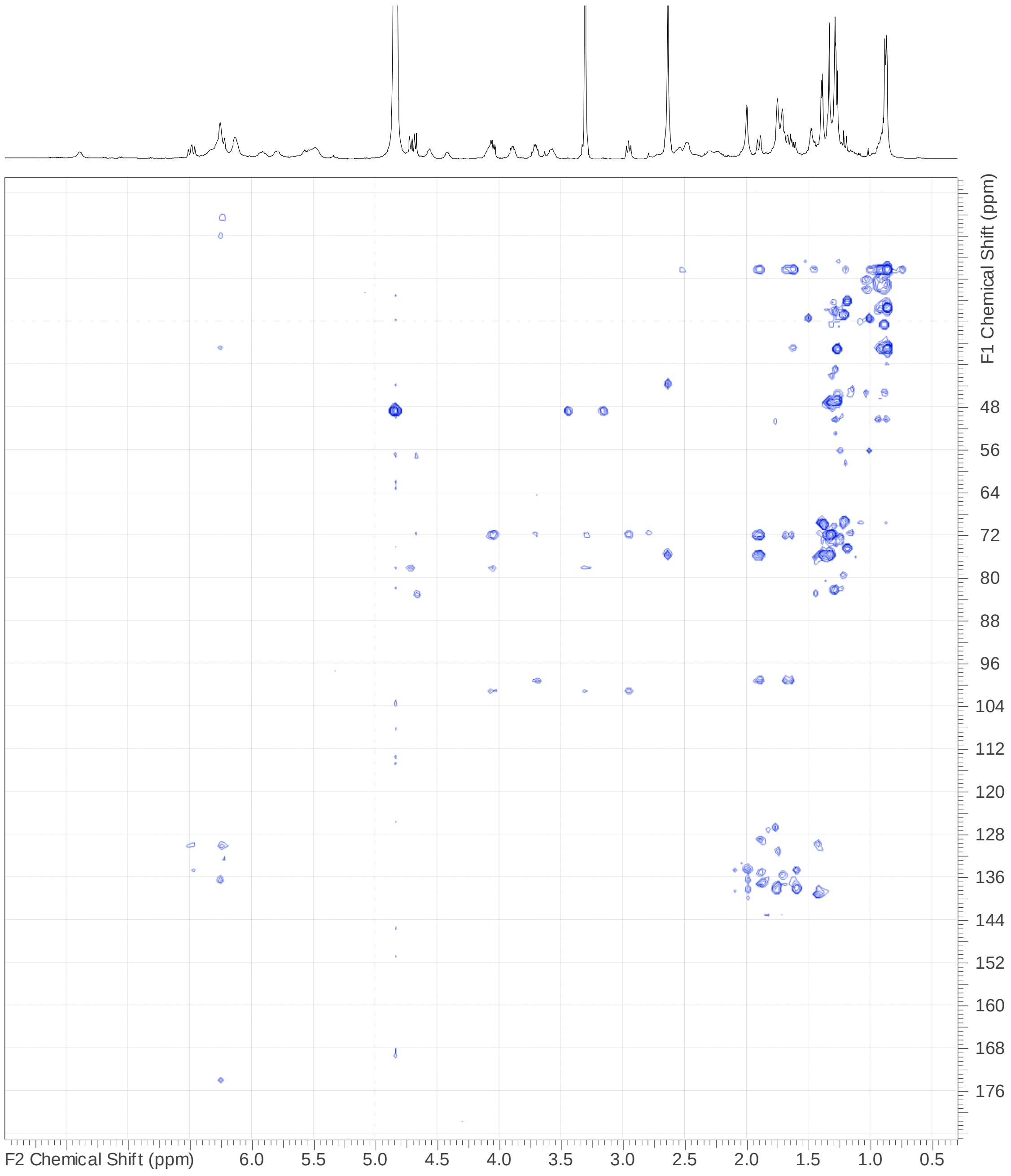

Supplementary Figure 9. HMBC spectrum of sipanmycin A (**11**).

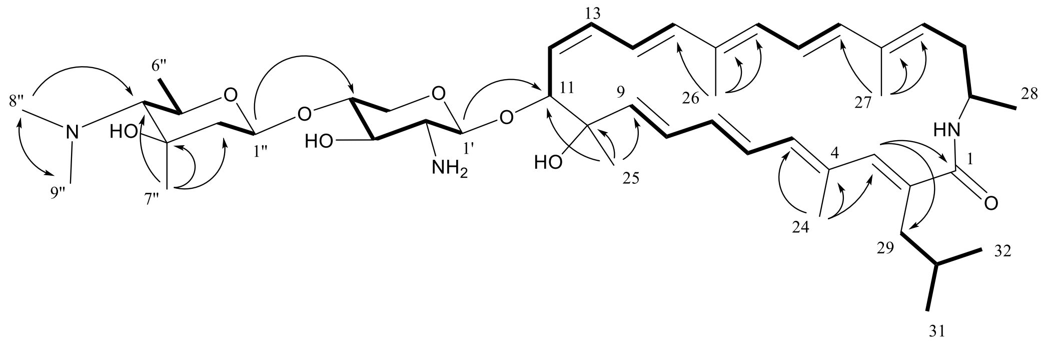

Supplementary Figure 10. Gross structure of sipanmycin A (**11**) determined by 2D-NMR. COSY correlations (further corroborated by the spin systems observed in the TOCSY spectrum) are indicated as bold bonds. Key HMBC correlations connecting independent spin systems are indicated by arrows.

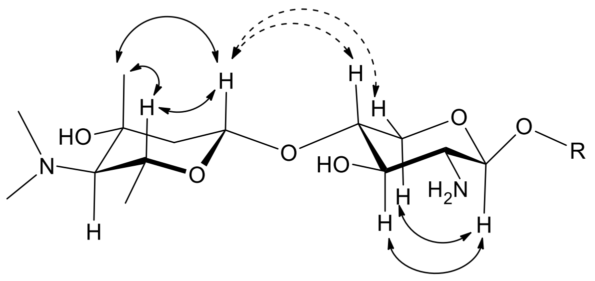

Supplementary Figure 11. Key intra-ring NOESY correlations (solid arrows) which, together with the observed coupling constants, allow stablishing the relative configuration of each monosaccharide. Key inter-ring NOESY correlations (dashed arrows) connecting both monosaccharides.

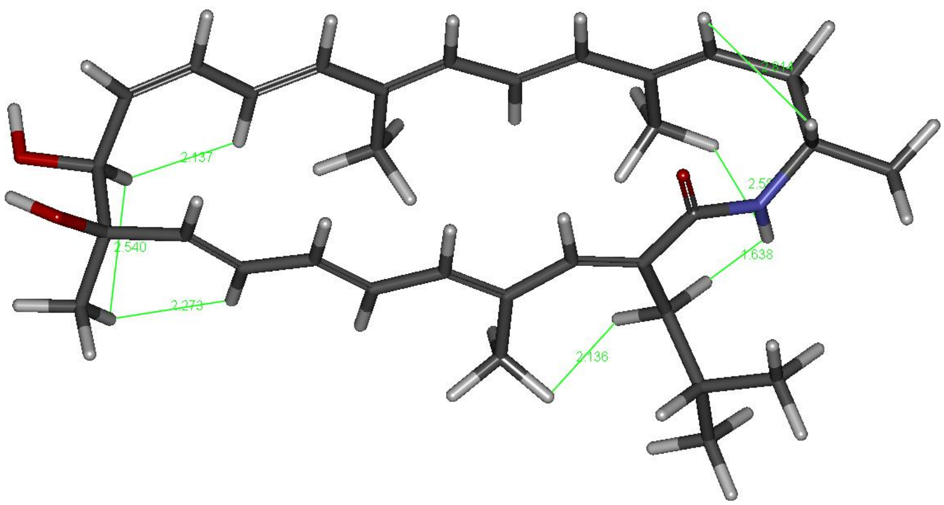

Supplementary Figure 12. Molecular model of sipanmycin A aglycone based on the reported and validated model of incednam (incednine aglycone) showing the same key NOEs (highlighted in green) as reported for incednam and confirming the expected identical absolute configuration of both macrolactams.

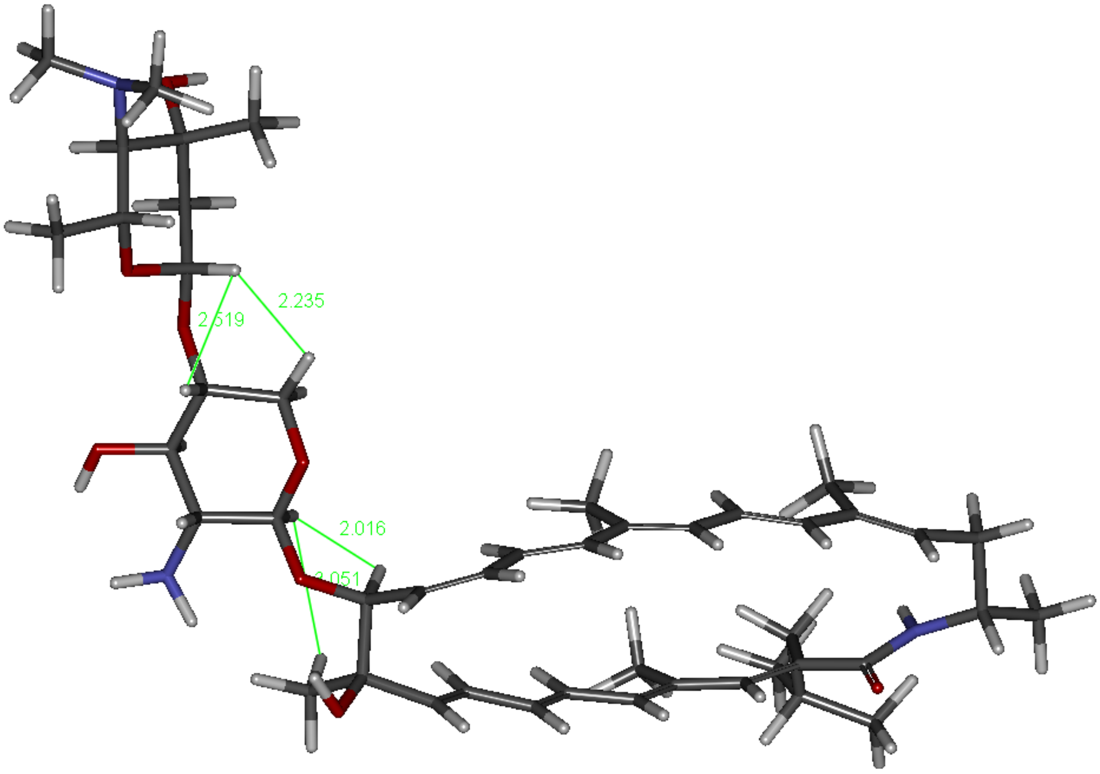

Supplementary Figure 13. Molecular model of sipanmycin A **(11)** based on the reported and validated model of incednine showing the same key NOEs (highlighted in green) as reported for incednine and confirming the expected D- configuration of both monosaccharides.

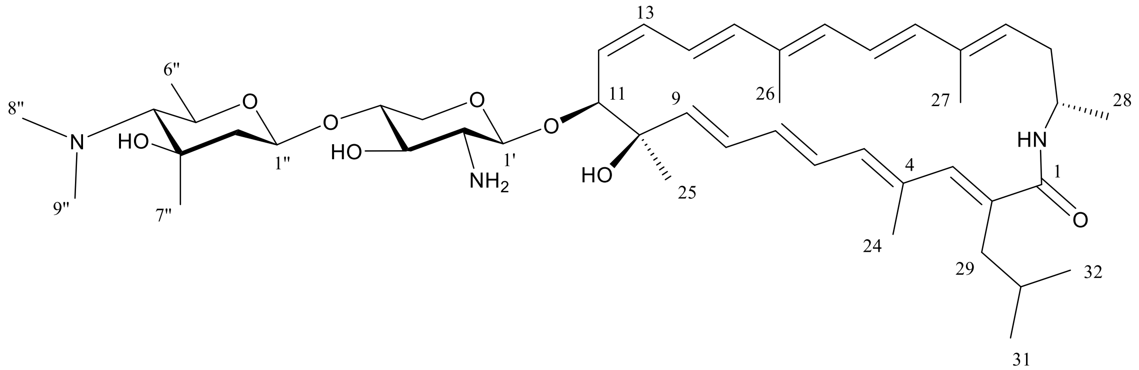

Supplementary Figure 14. Structure of sipanmycin A (**11**).

Supplementary Table 1. ^{1}H and ^{13}C NMR data for sipanmycin A (**11**) in CD_3OD at 24 C°.

Position	δ_C, type	δ_H (*J* in Hz)	Position	δ_C, type	δ_H (*J* in Hz)
1	174.2, C	-	1'	101.3, CH	4.68, d (7.8)
2	n.d., C	-	2'	57.5, CH	2.96, dd (9.2, 8.0)
3	138.6, CH	6.26, m	3'	72.0, CH	3.58, m
4	134.8, C	-	4'	78.3,CH	3.72, ddd (9.0, 8.5, 5.4)
5	136.6, CH	5.80, m	5'	64.4, CH_2	4.06, dd (12.4, 5.0)<br>3.32, dd (12.4, 10.0)
6	129.2, CH	6.49, dd (15.4, 11.5)	1''	99.3, CH	4.72,br d (9.7)
7	136.2, CH	5.91, m	2''	47.4, CH_2	1.91, br d (12.4)<br>1.67, m
8	130.1, CH	6.27, m	3''	72.3, C	-
9	141.2, CH	5.56, br d (15.8)	4''	75.7, CH	2.49, m
10	76.5, C	-	5''	69.9, CH	3.90, dq (9.7, 6.0)
11	83.3, CH	4.42, m	6''	20.9, CH_3	1.39, d (5.9)
12	129.9, CH	5.47, m	7''	22.5, CH_3	1.34, s
13	130.3, CH	6.14, m	8’’	43.8, CH_3	2.64, s
14	125.9, CH	6.13, m	9’’	43.8, CH_3	2.64, s
15	138.1, CH	6.24, m			
16	135.8, C	-			
17	132.8, CH	6.14, m			
18	124.5, CH	6.34, m			
19	138.6, CH	6.27, m			
20	137.5, C	-			
21	131.3, C	5.51, m			
22	37.4, CH_2	2.50, m<br>2.26, m			
23	47.2, CH	4.07, m			
24	16.2, CH_3	2.00, br s			
25	22.9, CH_3	1.49, br s			
26	12.8, CH_3	1.72, br s			
27	12.8, CH_3	1.76, br s			
28	20.6, CH_3	1.28, d (6.9)			
29	37.2, CH_2	2.56, m<br>2.31, m			
30	29.5, CH	1.65, m			
31	22.5, CH_3	0.88, d (6.4)			
32	22.5, CH_3	0.88, d (6.4)			
1-NH	-	7.39 br s*			

^{13}C chemical shifts obtained from HSQC and HMBC spectra.

* The amide proton exchanges very slowly and is clearly observed in the spectra.

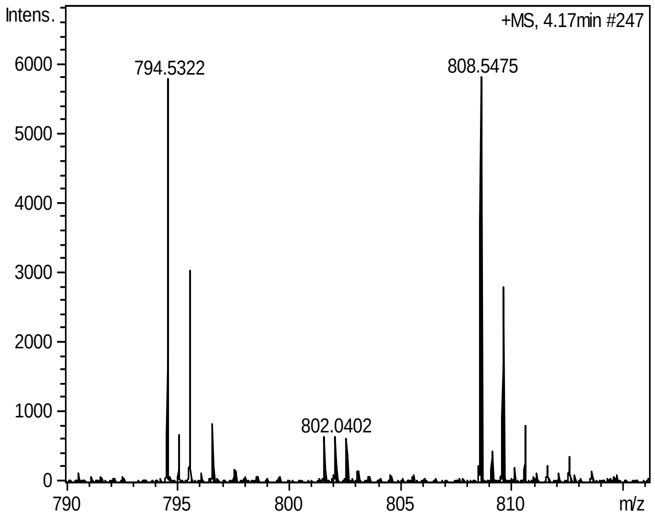

Supplementary Figure 15. HRMS spectrum of sipanmycin B (**12**)

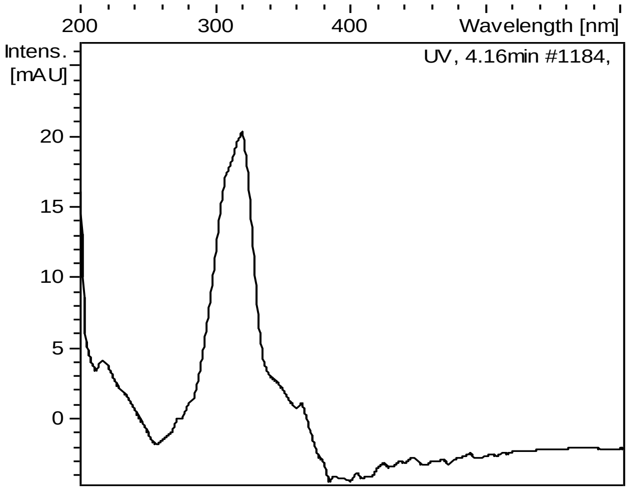

Supplementary Figure 16. UV-vis (DAD) spectrum of sipanmycin B (**12**).

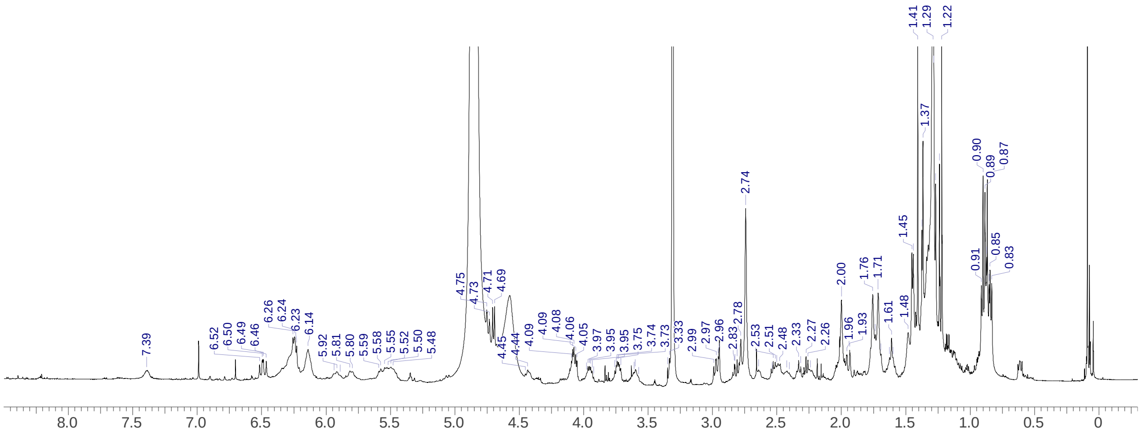

Supplementary Figure 17. ¹H NMR spectrum (CD_3OD, 500 MHz) of sipanmycin B (**12**).

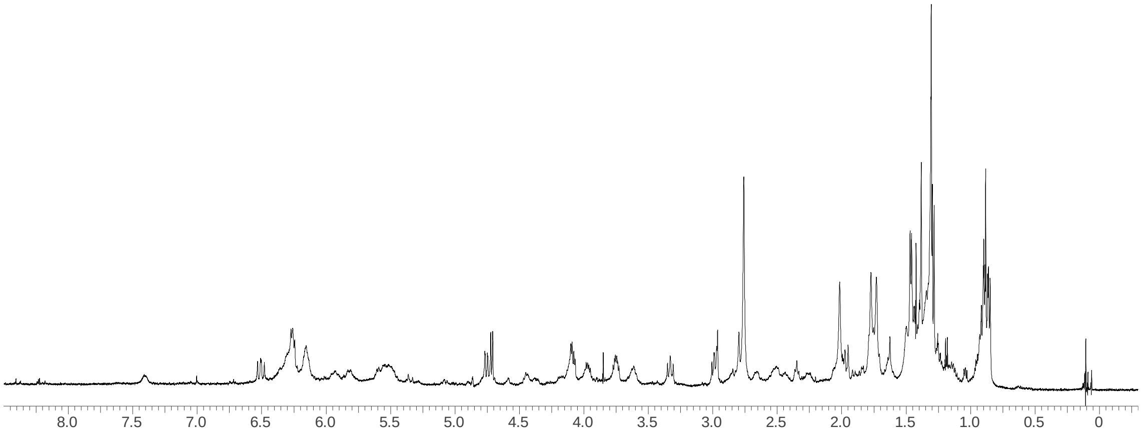

Supplementary Figure 18. Diffusion-filtered ¹H NMR spectrum of sipanmycin B (**12**).

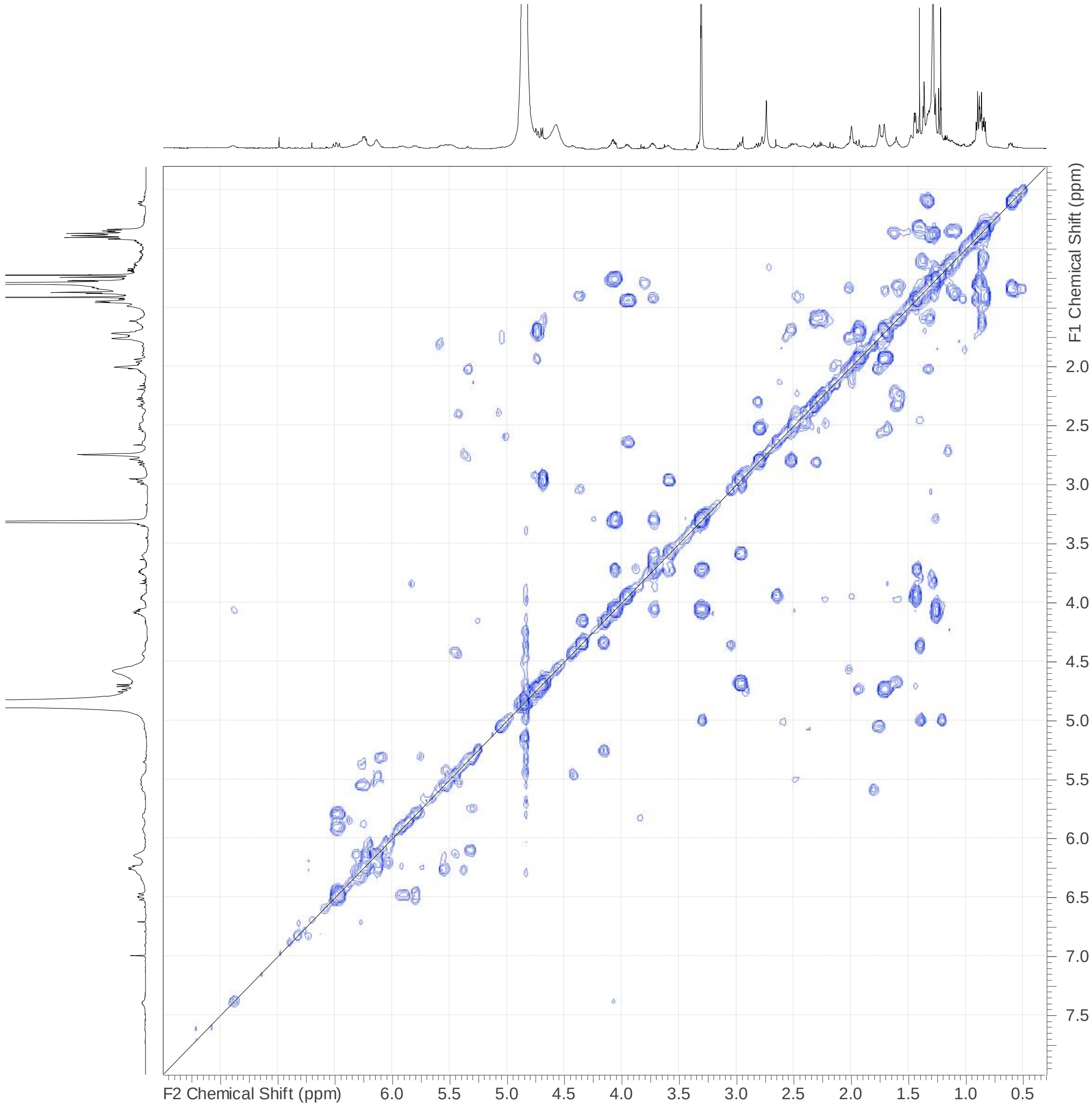

Supplementary Figure 19. COSY spectrum of sipanmycin B (**12**).

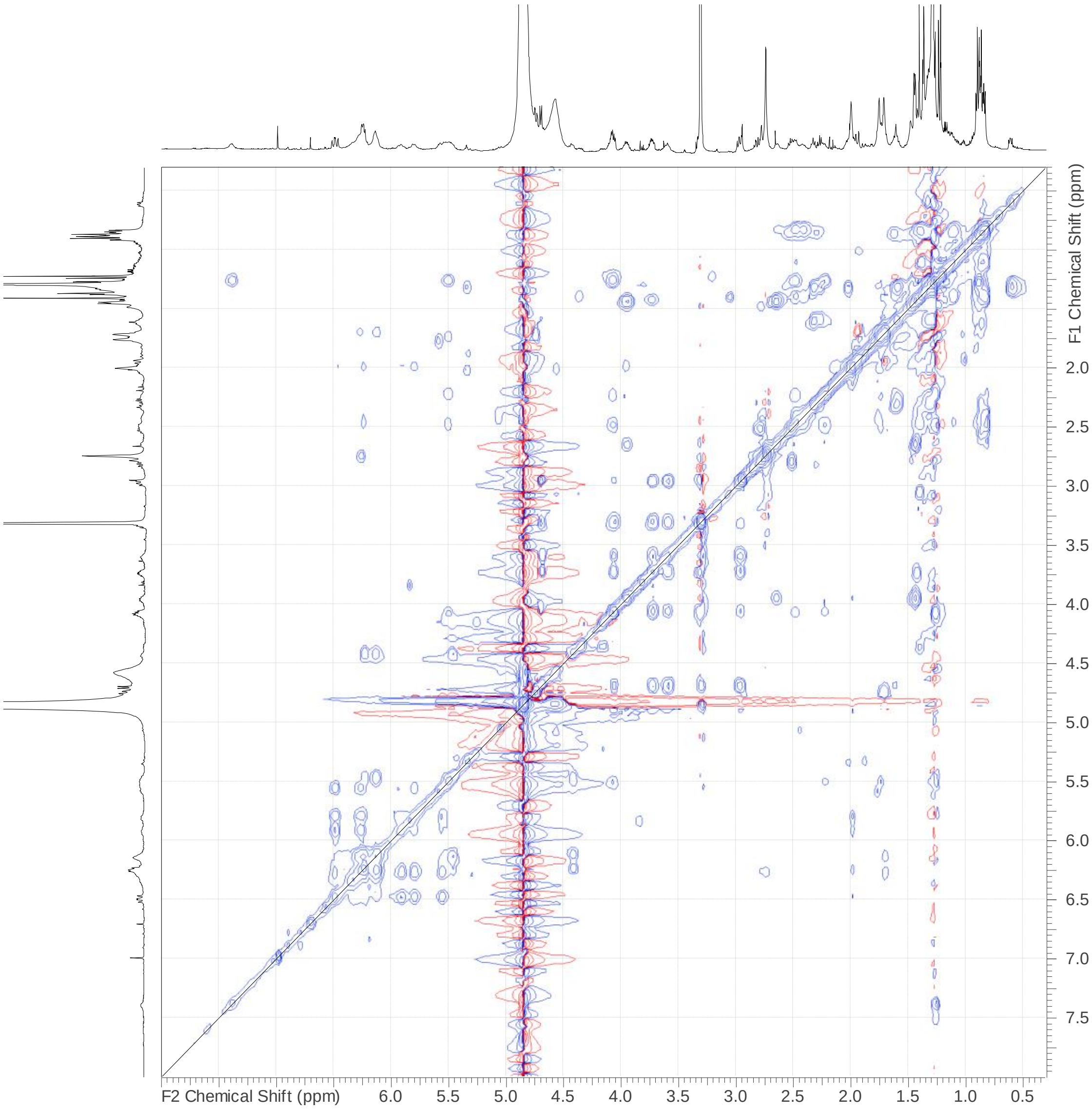

Supplementary Figure 20. TOCSY spectrum of sipanmycin B (**12**).

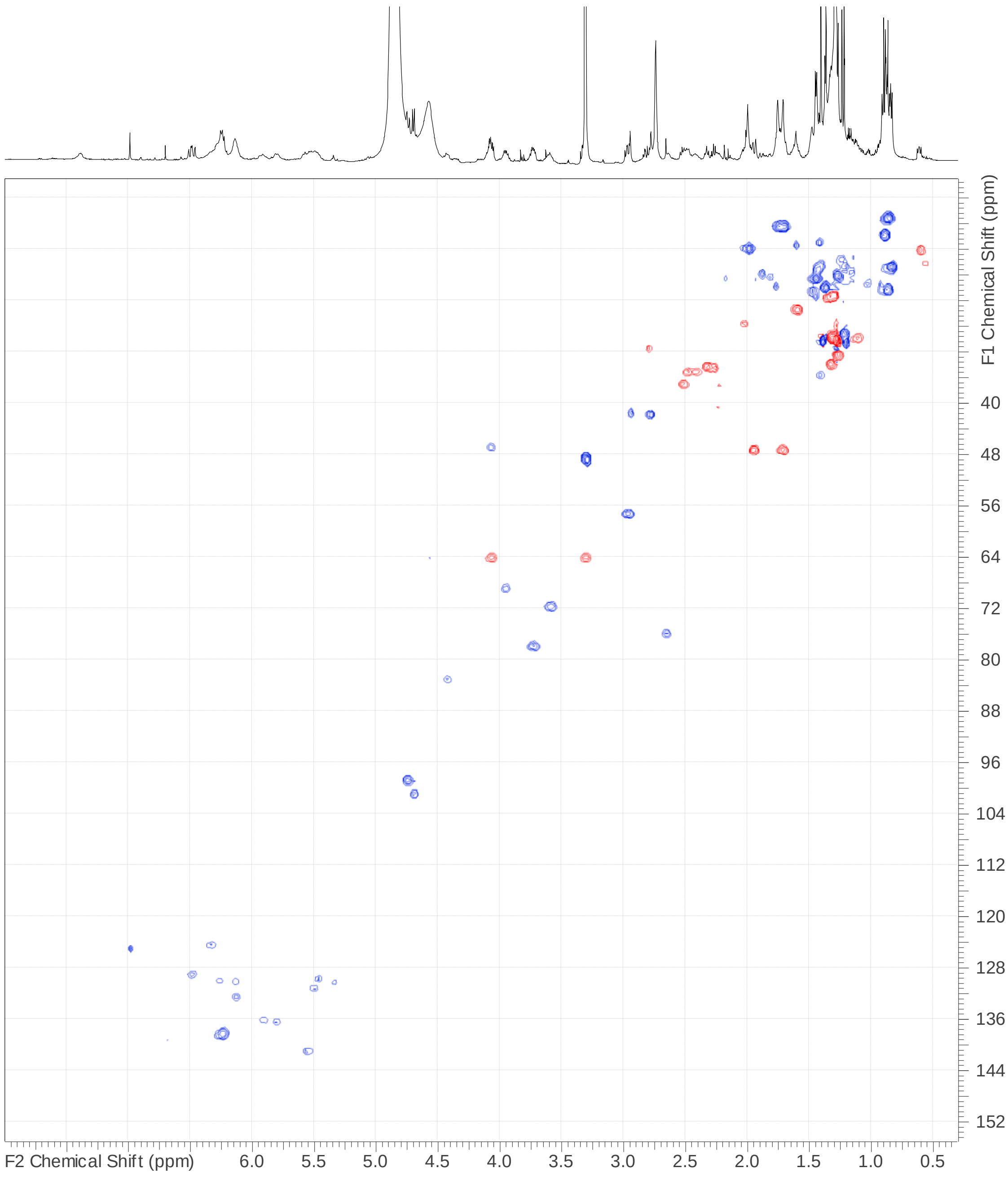

Supplementary Figure 21. Edited HSQC spectrum of sipanmycin B (**12**).

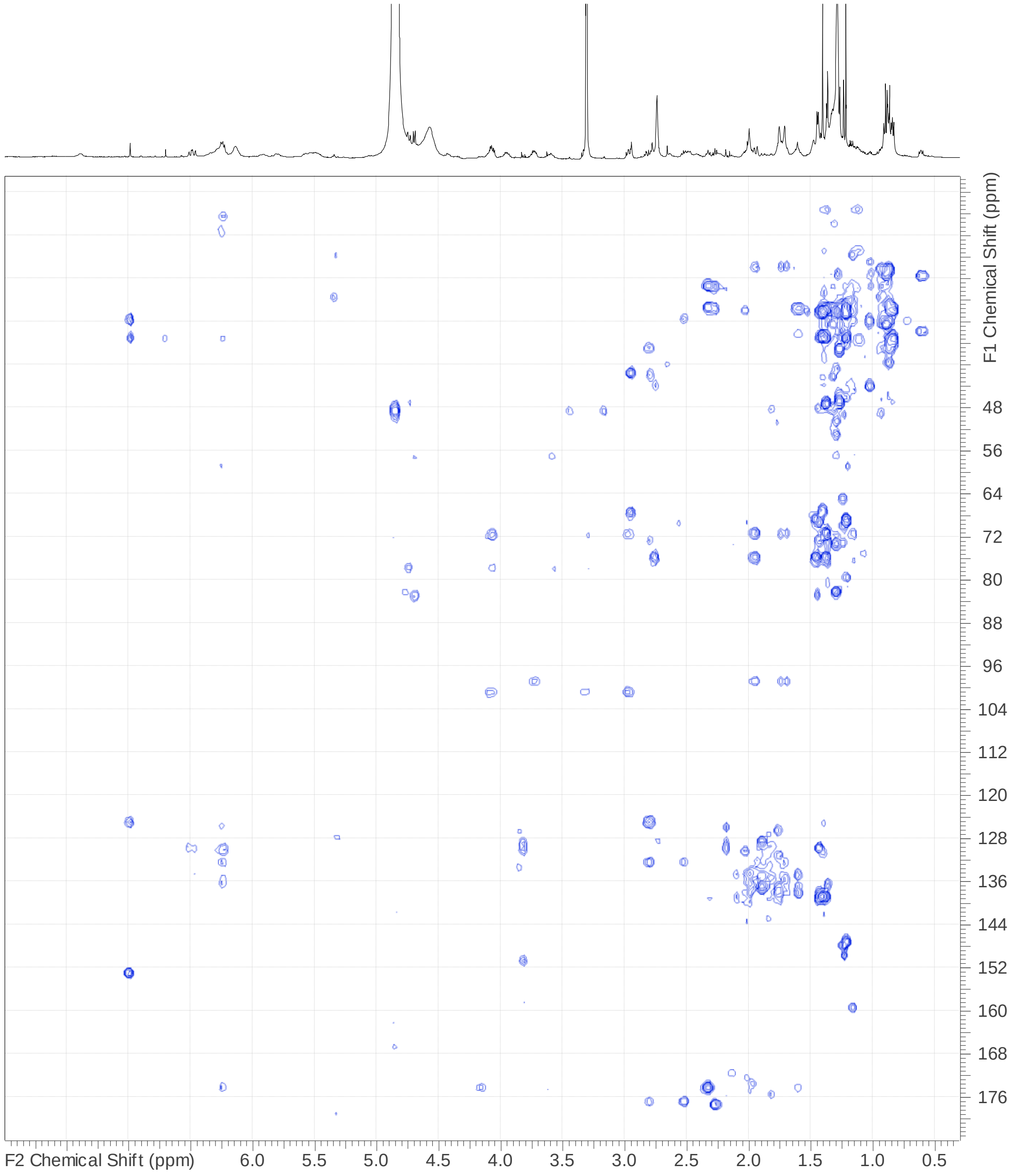

Supplementary Figure 22. HMBC spectrum of sipanmycin B (**12**).

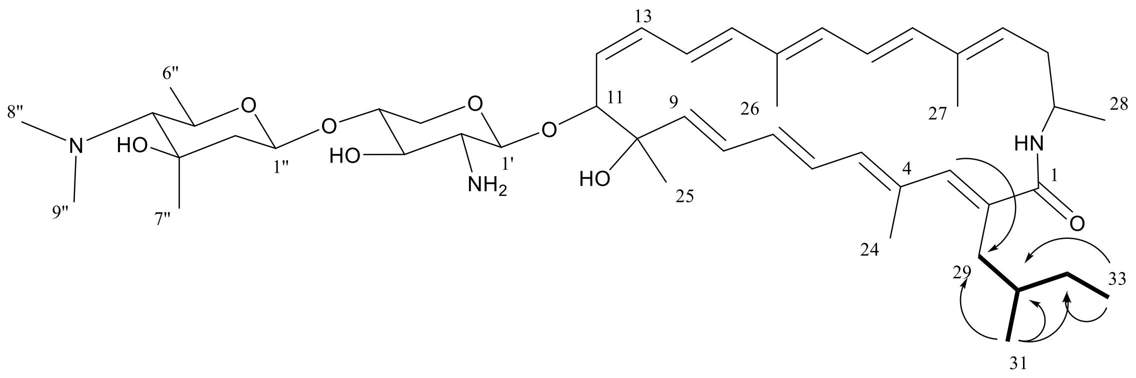

Supplementary Figure 23. Gross structure of sipanmycin B (**12**) determined by 2D-NMR. Only the COSY correlations (further corroborated by the spin systems observed in the TOCSY spectrum) of the side chain at C-2 are shown as bold bonds. Key HMBC correlations of this side chain are indicated by arrows. The COSY and key HMBC correlations of the rest of the structure are identical to those of sipanmycin A (**11**) and are not shown.

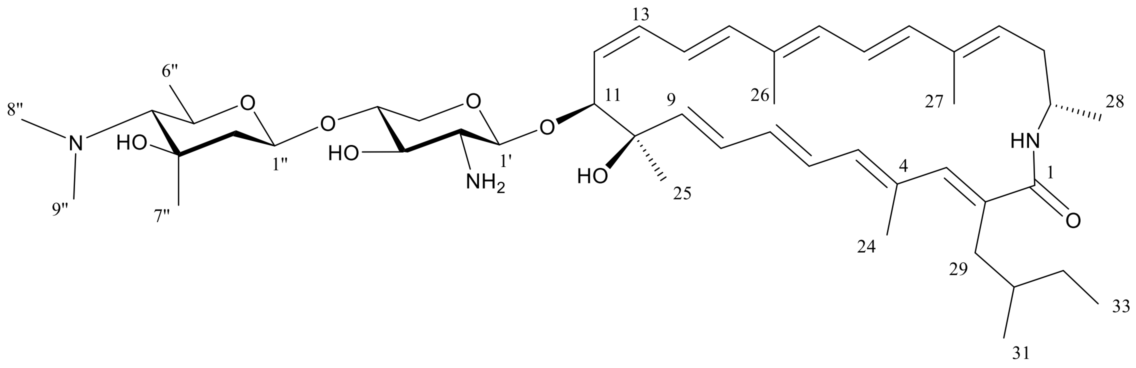

Supplementary Figure 24. Structure of sipanmycin B (**12**). The absolute configuration at C-30 could not be established.

Supplementary Table 2. ^{1}H and ^{13}C NMR data for sipanmycin B (**12**) in CD_3OD at 24 C°.

Position	δ_C, type	δ_H (*J* in Hz)	Position	δ_C, type	δ_H (*J* in Hz)
1	174.1, C	-	1'	101.1, CH	4.70, d (7.7)
2	n.d., C	-	2'	57.4, CH	2.97, dd (9.2, 7.9)
3	138.6, CH	6.26, m	3'	71.8, CH	3.60, m
4	134.7, CH	-	4'	78.0, CH	3.74, ddd (9.2, 8.5, 5.3)
5	136.6, CH	5.80, m	5'	64.3, CH_2	4.07, dd (11.8, 5.1)<br>3.31, m
6	129.2, CH	6.49, dd (15.4, 11.5)	1"	99.0, CH	4.74, dd (9.9, 1.8)
7	136.2, CH	5.91, m	2"	47.5 CH_2	1.95, br d (12.3)<br>1.72, m
8	130.1, CH	6.27, m	3"	71.6, C	-
9	141.2, CH	5.56, br d (15.8)	4"	76.1, CH	2.65, m
10	n. d., C	-	5"	69.1, CH	3.95, dq (10.0, 5.4)
11	83.3, CH	4.42, m	6"	20.8, CH_3	1.45, d (5.8)
12	129.8, CH	5.47, m	7"	22.2, CH_3	1.37, s
13	130.2, CH	6.14, m	8’’	44.1, CH_3	2.74, s
14	125.9, CH	6.13, m	9’’	44.1, CH_3	2.74, s
15	138.1, CH	6.24, m			
16	135.8, C	-			
17	132.8, CH	6.14, m			
18	124.5, CH	6.34, m			
19	138.6, CH	6.27, m			
20	137.5, C	-			
21	131.3, CH	5.51, m			
22	37.4, CH_2	2.52, m<br>2.24, m			
23	47.2, CH	4.07, m			
24	16.2, CH_3	2.00, br s			
25	22.9, CH_3	1.49, br s			
26	12.8, CH_3	1.71, br s			
27	12.8, CH_3	1.76, br s			
28	20.6, CH_3	1.28, d (6.9)			
29	35.3, CH_2	2.49, m<br>2.42, m			
30	35.8, CH	1.42, m			
31	19.0, CH_3	0.84, d (6.4)			
32	30.0, CH_2	1.12, m			
33	11.4, CH_3	0.87, m			
1-NH	-	7.39, br s*			

^{13}C chemical shifts obtained from HSQC and HMBC spectra.

* The amide proton exchanges very slowly and is clearly observed in the proton and the homonuclear 2D spectra.

Supplementary Table 3. Secondary metabolite biosynthesis gene clusters (BGCs) identified in *Streptomyces* sp. CS113 genome sequence (accession number NEVC00000000.1) using antiSMASH 4.0 analysis platform. Only BGCs with at least 40% of genes showing similarity are mentioned.

Cluster	Location B9W62_	Type	Most similar known BGC
1	00005-00235	Type I PKS-NRPS	Polyoxypeptin (40%) BGC0001036_c1
2	00480-00615	Other	-
3	01615-01785	NRPS	Coelichelin (100%) BGC0000325_c1
4	03005-03035	Bacteriocin	Informatipeptin (42%) BGC0000518_c1
5	03485-03680	Type I PKS-Other KS	Arsenopolyketides (83%) BGC0001283_c1
6	04715-04785	Lantipeptide	-
7	05250-05380	Terpene	Hopene (92%) BGC0000663_c1
8	05685-05780	Lantipeptide	SAL-2242 (100%) BGC0000546_c1
9	07175-07310	Terpene	Isorenieratene (100%) BGC0000664_c1
10	07670-07730	Siderophore	-
11	08425-08515	Indole	7-prenylisatin (80%) BGC0001294_c1
12	08545-08635	Terpene	-
13	08695-08750	Bacteriocin	-
14	09420-09600	Type I PKS	Undecylprodigiosin (100%) BGC0001063_c1
15	09975-10015	Siderophore	-
16	10030-10225	Type II PKS	Hatomarubigin (75%) BGC0000232_c1
17	11675-11910	NRPS	-
18	12370-12585	Type II PKS	Spore pigment (66%) BGC0000271_c1
19	12855-12955	Terpene	Albaflavenone (100%) BGC0000660_c1
20	24690-24740	Siderophore	Desferrioxamine B (100%) BGC0000940_c1
21	25140-25200	Melanin	Melanin (60%) BGC0000909_c1
22	29775-29825	Ectoine	Ectoine (100%) BGC0000853_c1
23	31835-32120	Type II PKS-Other KS-Oligosaccharide	Polyketomycin (41%) BGC0001061_c1
24	33280-33490	Type III PKS	-
25	33500-33665	NRPS	-
26	34545-34740	NRPS	Paenibactin (83%) BGC0000401_c1
27	35580-35685	Terpene	-
28	35925-35985	Butyrolactone	-
29	36865-36960	Terpene	Carotenoid (45%) BGC0000633_c1
30	37215-37330	Indole	-
31	38470-38695	NRPS	Coelibactin (100%) BGC0000324_c1
32	38955-39190	Lantipeptide-Other KS	-

Supplementary Table 4. Secondary metabolite biosynthesis gene clusters (BGCs) identified in *Streptomyces* sp. CS159 genome sequence (accession number NEVD00000000.1) using antiSMASH 4.0 analysis platform. Only BGCs with at least 40% of genes showing similarity are mentioned.

**Cluster**	**Location B9W64_**	**Type**	**Most similar known BGC**
1	00645-00815	NRPS	Coelichelin (100%) BGC0000325_c1
2	01970-02000	Bacteriocin	Informatipeptin (42%) BGC0000518_c1
3	02445-02635	Type I PKS-Other KS	Arsenopolyketides (83%) BGC0001283_c1
4	03970-04100	Terpene	Hopene (100%) BGC0000663_c1
5	04960-05240	NRPS-*trans* AT PKS	-
6	06245-06365	Terpene	Isorenieratene (100%) BGC0000664_c1
7	06590-06905	Siderophore-NRPS	-
8	07665-07760	Terpene	-
9	07820-07870	Bacteriocin	-
10	08490-08825	Type I PKS-NRPS	Undecylprodigiosin (100%) BGC0001063_c1
11	09130-09170	Siderophore	-
12	11190-11400	Type II PKS	Spore pigment (66%) BGC0000271_c1
13	11660-11760	Terpene	Albaflavenone (100%) BGC0000660_c1
14	15570-15745	Other	-
15	16360-16575	NRPS	-
16	22580-22785	Type II PKS	-
17	23735-23785	Siderophore	Desferrioxamine B (100%) BGC0000940_c1
18	24165-24225	Melanin	Melanin (60%) BGC0000909_c1
19	28645-28695	Ectoine	Ectoine (100%) BGC0000853_c1
20	31815-32020	Type III PKS	-
21	34290-34385	Terpene	Carotenoid (54%) BGC0000633_c1
22	34640-34755	Indole	-
23	35470-35580	Terpene	2-methylisoborneol (100%) BGC0000658_c1
24	36620-36780	Type II PKS	-
25	36835-36880	Butyrolactone	-
26	36920-37120	Type II PKS-Oligosaccharide	Lomaiviticin (44%) BGC0000240_c1

Supplementary Table 5. Secondary metabolite biosynthesis gene clusters (BGCs) identified in *Streptomyces* sp. CS227 genome sequence (accession number NEVE01000000.1) using antiSMASH 4.0 analysis platform. Only BGCs with at least 40% of genes showing similarity are mentioned.

**Cluster**	**Location B9W68_**	**Type**	**Most similar known BGC**
1	00545-00795	NRPS	-
2	01075-01395	Terpene-NRPS	-
3	01800-02060	NRPS	-
4	02140-02295	Type I PKS-NRPS	SGR PTMs (100%) BGC0001043_c1
5	02475-02605	Terpene	Hopene (76%) BGC0000663_c1
6	02910-02940	Bacteriocin	-
7	04695-04745	Bacteriocin	-
8	05545-05710	Type I PKS-Terpene	-
9	06150-06200	Siderophore	-
10	07250-07370	Terpene	-
11	08695-08790	Terpene	Albaflavenone (100%) BGC0000660_c1
12	10895-11025	Thiopeptide	-
13	11745-11825	Bacteriocin	-
14	12425-12520	Lantipeptide	SAL-2242 (100%) BGC0000546_c1
15	14240-14435	NRPS	-
16	17550-17755	NRPS	-
17	21075-21125	Siderophore	Desferrioxamine B (100%) BGC0000941_c1
18	24895-24945	Ectoine	Ectoine (100%) BGC0000853_c1
19	28470-28655	Bacteriocin-Terpene	Isorenieratene (85%) BGC0000664_c1
20	28985-29775	Type III PKS-Lantipeptide-Type I PKS-NRPS	Candicidin (100%) BGC0000034_c1
21	29780-30285	Type I PKS	Stambomycin (68%) BGC0000151_c1
22	30415-30445	Butyrolactone	-

Supplementary Table 6. Secondary metabolite biosynthesis gene clusters (BGCs) identified in *Streptomyces* sp. CS057 genome sequence (accession number NEVF00000000.1) using antiSMASH 4.0 analysis platform. Only BGCs with at least 40% of genes showing similarity are mentioned.

**Cluster**	**Location B9W61_**	**Type**	**Most similar known BGC**
1	00310-00505	NRPS-Type I PKS	-
2	00750-00980	*trans* AT PKS-Lantipeptide	Cycloheximide/actiphenol (94%)BGC0000175_c1
3	01095-01380	Type III PKS-NRPS	-
4	01540-01585	Melanin	Melanin (100%) BGC0000911_c1
5	01720-02110	Type I PKS -NRPS	-
6	02205-02385	Type I PKS	-
7	02815-02875	Bacteriocin	-
8	03265-03625	Type I PKS-NRPS	SGR PTM (100%) BGC0001043_c1
9	03825-03985	NRPS	Nucleocidin (47%) BGC0001387_c1
10	04140-04255	Terpene	Hopene (69%) BGC0000663_c1
11	05385-05575	Type II PKS	Nonactin (92%) BGC0000252_c1
12	05800-05905	Terpene	Methylisoborneol (100%) BGC0000658_c1
13	06835-06880	Bacteriocin	-
14	07105-07440	Type II PKS-Oligosaccharide-NRPS	Landomycin (62%) BGC0000239_c1
15	08295-08345	Siderophore	-
16	10070-10160	Terpene	-
17	11875-11965	Lantipeptide	AmfS (100%) BGC0000496_c1
18	12155-12225	Melanin	Melanin (100%) BGC0000912_c1
19	12325-12425	Thiopeptide	-
20	12460-12650	NRPS	-
21	16735-17285	Ectoine-Arylpolyene-Type I PKS-NRPS-Butyrolactone-Ladderane	Skyllamycin (95%) BGC0000429_c1
22	20710-20770	Ectoine	Ectoine (75%) BGC0000853_c1
23	24205-24255	Siderophore	Desferrioxamine B (100%) BGC0000941_c1
24	24610-24690	Lantipeptide	-
25	25950-26220	Lantipeptide-NRPS	-
26	29050-29105	Ectoine	Ectoine (100%) BGC0000853_c1
27	31140-31220	Terpene	-
28	32525-32660	NRPS	-
29	32770-32895	Thiopeptide	-
30	33900-34065	Type III PKS	-
31	34600-34820	NRPS	Griseobactin (94%) BGC0000368_c1
32	35080-35175	Terpene	-
33	35345-35395	Butyrolactone	-

Supplementary Table 7. Cervimycin biosynthesis gene cluster in *Streptomyces* sp. CS113 genome sequence (accession number NEVC00000000.1). Comparison against proteins encoded in the polyketomycin (*Streptomyces diastatochromogenes* Tü6028) and dutomycin (*Streptomyces minoensis*) biosynthesis gene clusters. The level of identity/similarity is shown in parenthesis (%).

Protein	Location B9W62_	Proposed function	Polyketomycin	Dutomycin
Cvm1	31925	Beta-ACP synthase (KS)	PokP1 (69/79)	DutA (68/78)
Cvm2	31930	Beta-ketoacyl synthase	PokP2 (59/73)	DutB (57/73)
Cvm3	31935	Acyl carrier protein	PokP3 (46/61)	DutC (45/62)
Cvm4	31940	Asparagine synthase	-	-
Cvm5	31945	NDP-hexose 3,4-dehydratase	PokS5 (73/81)	DutS5 (72/81)
Cvm6	31950	Beta-ketoacyl synthase III	PokM2 (34/46)	DutH (34/49)
Cvm7	31955	Ketoreductase	PokT2 (54/66)	DutJ (53/64)
Cvm8	31960	Glycosyltransferase	PokGT1 (43/52)	DutGT1 (40/51)
Cvm9	31965	Acyl-CoA dehydrogenase	PokU2 (49/60)	DutR (47/59)
Cvm10	31970	O-methyltransferase	PokMT3 (66/75)	DutMT2 (66/75)
Cvm11	31975	Glycosyltransferase	PokGT1 (45/56)	DutGT1 (45/56)
Cvm12	31980	NDP-4-keto-6-deoxyhexose reductase	PokS6 (50/60)	DutS6 (49/58)
Cvm13	31985	NDP-hexose 3,5-epimerase	PokS7 (49/63)	DutS7 (48/61)
Cvm14	31990	Transcriptional regulator (AfsR family)	PokR1 (45/56)	DutV (45/56)
Cvm15	31995	Glycosyltransferase	PokGT2 (45/59)	DutGT2 (45/60)
Cvm16	32000	Glycosyltransferase	PokGT2 (41/56)	DutGT2 (39/56)
Cvm17	32005	Transcriptional regulator (PadR family)	-	-
Cvm18	32010	Multidrug MFS transporter	-	-
Cvm19	32015	Glycosyltransferase	PokGT2 (45/55)	DutGT2 (44/55)
Cvm20	32020	Glucose-1-phosphate thymidylyltransferase	PokS1 (36/55)	DutS1 (36/54)
Cvm21	32025	Transcriptional regulator (LuxR family)	-	-
Cvm22	32030	Transcriptional regulator (SARP family)	PokR2 (38/54)	DutK (37/55)
Cvm23	32035	NDP-hexose 3-ketoreductase	PokS4 (53/61)	DutS4 (52/62)
Cvm24	32040	dTDP-hexose 2,3-dehydratase	PokS3 (52/66)	DutS3 (53/69)
Cvm25	32045	dTDP-D-glucose 4,6-dehydratase	PokS2 (63/74)	DutS2 (62/74)
Cvm26	32050	Acyl transferase domain PKS	PokM1 (34/46)	DutG (31/48)
Cvm27	32055	Aromatase	PokC3 (48/61)	DutD (46/60)
Cvm28	32060	Hydroxyacyl-CoA dehydrogenase	PokC1 (57/62)	DutE (58/65)
Cvm29	32065	Cyclase	PokC2 (66/81)	DutF (67/81)
Cvm30	32070	Monooxygenase	PokO2 (47/63)	DutO2 (50/65)
Cvm31	32075	Acyl-CoA ligase	PokL (48/58)	DutL (47/57)
Cvm32	32080	Monooxygenase	PokO3 (46/65)	DutO3 (45/66)
Cvm33	32085	Methylmalonyl-CoA carboxyltransferase	PokAC1 (26/39)	DutM (26/40)

Supplementary Table 8. Lomaiviticin-like biosynthesis gene cluster in *Streptomyces* sp. CS159 genome sequence (accession number NEVD00000000.1). Comparison against proteins encoded in an unknown cluster from *Streptomyces* sp. NRRL WC-3753 and lomaiviticin (*Salinispora pacifica*) biosynthesis gene cluster. The level of identity/similarity is shown in parenthesis (%).

Protein	Location B9W64_	Proposed function	*Streptomyces* sp. NRRL WC-3753	Lomaiviticin
Orf1	36920	Anthrone monooxygenase	WP_054102549 (99/100)	Lom28 (55/67)
Orf2	36925	Cyclase	-	Lom21 (70/81)
Orf3	36930	Acyl carrier protein	-	Lom60 (45/56)
Orf4	36935	Ketoacyl reductase	-	Lom22 (70/78)
Orf5	36940	Cyclase/dehydrase	WP_054102550 (99/99)	Lom25 (55/66)
Orf6	36945	Monooxygenase	WP_054102551 (99/99)	Lom26 (50/59)
Orf7	36950	Monooxygenase	WP_054102580 (99/99)	Lom27 (55/66)
Orf8	36955	O-methyltransferase	WP_042823310 (99/100)	Lom23 (51/68)
Orf9	36960	4Fe-4S-ferredoxin	-	Lom30 (73/80)
Orf10	36965	Hypothetical protein	KPC71350 (98/98)	Lom29 (60/68)
Orf11	36970	Amidase	WP_051005805 (99/99)	Lom33 (62/72)
Orf12	36975	L-glutamine synthetase	WP_054102553 (98/98)	Lom32 (62/71)
Orf13	36980	Adenylosuccinate lyase	WP_063789679 (99/99)	Lom34 (71/80)
Orf14	36985	N-acetyltransferase	WP_054102554 (98/100)	Lom35 (64/75)
Orf15	36990	Transcriptional regulator (OmpR family)	-	-
Orf16	36995	Peptidase	-	-
Orf17	37000	Esterase	-	-
Orf18	37005	Transcriptional regulator (HlxR family)	-	-
Orf19	37010	Hypothetical protein	-	-
Orf20	37015	Hypothetical protein	WP_054102556 (98/98)	-
Orf21	37020	Apoprotein precursor	-	-
Orf22	37025	Glyoxalase	WP_054102557 (99/100)	Lom46 (32/45)
Orf23	37030	Transcriptional regulator (AraC family)	WP_054102558 (96/97)	Lom64 (53/62)
Orf24	37035	Glyoxalase/bleomycin resistance protein/dioxygenase	-	Lom45 (62/81)
Orf25	37040	Transcriptional regulator (AraC family)	WP_054102559 (94/95)	Lom8 (51/65)
Orf26	37045	Nuclease	WP_054102560 (100/100)	Lom15 (52/65)
Orf27	37050	ABC transporter	WP_054102583 (99/99)	Lom3 (59/71)
Orf28	37055	ABC transporter	WP_054102561 (100/100)	Lom4 (69/80)
Orf29	37060	Peptidase/Glutamine amidotransferase	-	Lom12 (66/73)
Orf30	37065	Carboxymuconolactone decarboxylase	-	Lom11 (58/73)
Orf31	37070	NDP-hexose-2,3-dehydratase	WP_054102584 (99/99)	Lom9 (74/81)

Orf32	37075	dTDP-glucose-4,6-dehydratase	WP_054102562 (99/100)	Lom57 (60/72)
Orf33	37080	Glyoxalase	-	-
Orf34	37085	Transporter	WP_054102563 (99/100)	Lom47 (60/72)
Orf35	37090	Glycosyltransferase	WP_054102564 (98/99)	Lom57 (28/42)
Orf36	37095	Hypothetical protein	-	-
Orf37	37100	Phytanoyl-CoA dioxygenase	WP_007390197 (99/100)	-
Orf38	37105	Hypothetical protein (sulfatase)	WP_054102565 (98/98)	-
Orf39	37110	MFS transporter	WP_063789682 (99/100)	-
Orf40	37115	Oxidoreductase	WP_054102567 (98/98)	-
Orf41	37120	Aromatic ring hydroxylase	WP_054102568 (99/99)	Lom13 (63/75)
Orf42	37125	Protoporphyrinogen oxidase	WP_054102569 (99/99)	Lom14 (61/73)
Orf43	37130	Monooxygenase	WP_054102570 (62/68)	Lom16 (69/77)
Orf44	37135	Monooxygenase	WP_054102570 (90/89)	Lom17 (65/73)
Orf45	37140	Short chain dehydrogenase/ reductase SDR	WP_051005800 (100/100)	Lom18 (72/83)
Orf46	37145	Transcriptional regulator (NmrA family)	WP_054102585 (99/98)	Lom19 (68/76)
Orf47	37150	Oxidoreductase	WP_054102586 (99/99)	Lom20 (69/79)
Orf48	37155	O-methyltransferase	WP_054102571 (99/99)	Lom23 (63/76)
Orf49	37160	Alkylhydroperoxidase	-	Lom11 (56/70)
Orf50	37165	Hypothetical protein	WP_054102572 (99/100)	Lom10 (62/77)
Orf51	37170	dTDP-4-dehydrorhamnose 3,5 epimerase	WP_054102573 (100/100)	Lom52 (48/60)
Orf52	37175	NDP-hexose-3-ketoreductase	WP_054102574 (99/100)	Lom53 (45/59)
Orf53	37180	Glucose-1-phosphate tymidylyltransferase	WP_054102575 (99/100)	Lom65 (65/80)
Orf54	37185	Glycosyltransferase	WP_063789680 (99/99)	Lom55 (53/66)
Orf55	37190	Glycosyltransferase	WP_063789680 (46/63)	Lom48 (48/61)
Orf56	37195	Hypothetical protein	-	-
Orf57	37200	Phenoxazinone synthase	WP_063789683 (99/99)	-
Orf58	37205	ABC transporter	WP_054102577 (99/100)	Lom43 (69/81)

Supplementary Table 9. Stambomycin-like biosynthesis gene cluster in *Streptomyces* sp. CS227 genome sequence (accession number NEVE01000000.1). Comparison against proteins encoded in the stambomycin (*Streptomyces ambofaciens* ATCC 23877) biosynthesis gene cluster. The level of identity/similarity is shown in parenthesis (%). The asterisks represent regions containing several genes that represent individual type I PKS domains belonging to a single gene do not properly annotated by the PGAAP pipeline.

Protein	Location B9W68_	Proposed function	Stambomycin
Orf1	29780	Transcriptional regulator (TetR family)	-
Orf2	29785	Transcriptional regulator (SARP family)	-
Orf3	29790	Transcriptional regulator (TetR family)	-
Orf4	29795	Hypothetical protein (ACP)	-
Orf5	29800	Beta-ketoacyl synthase	SAMR0482 (22/42)
Orf6	29805	4-phosphopantetheinyl transferase	-
Orf7	29810	Hypothetical protein (AMP binding domain)	-
Orf8	29815	Transcriptional regulator (SARP family)	-
Orf9	29820	dTDP-glucose 4,6-dehydratase	SAMR0486 (65/73)
Orf10	29825	Glucose-1-phosphate thymidylyltransferase	SAMR0487 (40/56)
Orf11	29830	Cytochrome P450	SAMR0478 (87/90)
Orf12	29835	Type I polyketide synthase	SAMR0477 (80/85)
Orf13	29840-29895*	Type I polyketide synthase	SAMR0477 (80/85)
Orf14	29900	Type I polyketide synthase	SAMRCDS2 (73/80)
Orf15	29905	Type I polyketide synthase	SAMR0476 (74/81)
Orf16	29910	Type I polyketide synthase	SAMR0476 (72/80)
Orf17	29915	ABC transporter ATP-binding protein	-
Orf18	29920	Hypothetical protein	-
Orf19	29925-30005*	Type I polyketide synthase	SAMR0467 (79/84)
Orf20	30010	Type I polyketide synthase	SAMR0466 (25/28)
Orf21	30015	Type I polyketide synthase	-
Orf22	30020	Type I polyketide synthase	SAMR0475 (55/65)
Orf23	30035	Type I polyketide synthase	SAMRCDS1 (72/79)
Orf24	30030-30125*	Type I polyketide synthase	SAMR0474 (77/83)
Orf25	30130	ABC transporter ATP-binding protein	-
Orf26	30135	ABC transporter	-
Orf27	30140	dTDP-4-dehydrorhamnose 3,5-epimerase	-
Orf28	30145	NDP-4-keto-6-deoxyhexose 4-ketoreductase	-
Orf29	30150	Glycosyltransferase	SAMR0481 (81/89)
Orf30	30155	N-methyltransferase	SAMR0472 (46/58)
Orf31	30160	NDP-hexose 2,3-dehydratase	-
Orf32	30165	NDP-deoxyhexose 3-aminotransferase	SAMR0480 (82/89)
Orf33	30170	Cytochrome P450	SAMR0479 (86/92)
Orf34	30175	Thioesterase	SAMR0485 (59/74)
Orf35	30180	Crotonyl-CoA carboxylase/reductase	SAMR0483 (17/40)
Orf36	30185-30270*	Type I polyketide synthase	SAMR0465 (68/73)
Orf37	30275	Transcriptional regulator (LuxR family)	SAMR0484 (60/70)

Supplementary Table 10. Warkmycin biosynthesis gene cluster in *Streptomyces* sp. CS057 genome sequence (accession number NEVF00000000.1). Comparison against proteins encoded in an unknown cluster from *Streptomyces* sp. MNU77 and landomycin (*Streptomyces cyanogenus*) biosynthesis gene cluster. The level of identity/similarity is shown in parenthesis (%).

Protein	Location B9W61_	Proposed function	*Streptomyces* sp. MNU77	Landomycin
Wmc1	07160	Carbamoyltransferase	WP_047179340 (99/99)	-
Wmc2	07165	Methyltransferase	WP_047179339 (99/99)	-
Wmc3	07170	dTDP-6-deoxy-L-hexose 3-O-methyltransferase	WP_047179338 (100/100)	-
Wmc4	07175	dTDP-4-keto-6-deoxyhexose reductase	WP_079190940 (99/99)	LanZ3 (37/45)
Wmc5	07180	Glucose-1-phosphate thymidylyltransferase	WP_063780464 (100/100)	LanG (35/54)
Wmc6	07185	dTDP-4-dehydrorhamnose 3,5-epimerase	WP_047179337 (99/100)	LanZ1 (52/56)
Wmc7	07190	C-glycosyltransferase	WP_047179336 (99/99)	LanGT2 (50/66)
Wmc8	07195	C-glycosyltransferase	WP_047179335 (99/99)	LanGT2 (45/60)
Wmc9	07200	Cytochrome P450	WP_047179334 (100/100)	-
Wmc10	07205	O-glycosyltransferase	WP_047179333 (99/99)	LanGT1 (49/65)
Wmc11	07210	Glycosyltransferase	WP_079191107 (99/99)	LanGT4 (31/42)
Wmc12	07215	Transcriptional regulator (TetR family)	WP_047179331 (99/100)	LanK (53/70)
Wmc13	07220	O-methyltransferase	WP_047179330 (99/99)	-
Wmc14	07225	dTDP-hexose-3-ketoreductase	WP_063780463 (99/98)	LanT (61/73)
Wmc15	07230	NDP-hexose 2,3-dehydratase	WP_047181060 (100/100)	LanS (70/78)
Wmc16	07235	NDP-hexose 4-ketoreductase	WP_047179329 (100/100)	LanR (67/77)
Wmc17	07240	NDP-hexose 3,4-dehydratase	WP_047179328 (99/100)	LanQ (85/92)
Wmc18	07245	dTDP-glucose 4,6-dehydratase	WP_047179327 (100/100)	LanH (72/82)
Wmc19	07250	Aldo/keto reductase	WP_047181059 (99/100)	-
Wmc20	07255	NADPH-dependent oxidoreductase	WP_047179326 (100/100)	LanO (64/74)
Wmc21	07260	Acyltransferase	WP_047179325 (99/99)	-
Wmc22	07265	MFS transporter	WP_052658878 (99/98)	-
Wmc23	07270	Oxidoreductase	OLO35362 (99/99)	LanM (58/67)
Wmc24	07275	Cyclase	WP_047179323 (100/100)	LanL (69/79)
Wmc25	07280	Ketoacyl reductase	-	LanD (79/84)
Wmc26	07285	Acyl carrier protein	WP_047179321 (100/100)	LanC (53/67)
Wmc27	07290	Beta-ketoacyl synthase	WP_047179320 (100/100)	LanB (67/81)
Wmc28	07295	Beta-ACP synthase	WP_047179319 (100/100)	LanA (75/83)
Wmc29	07300	Aromatase	WP_047179318 (100/100)	LanF (79/86)
Wmc30	07305	Monooxygenase	WP_047179317 (99/100)	LanE (66/75)
Wmc31	07310	Hypothetical protein	WP_052658877 (98/98)	-
Wmc32	07315	Transcriptional regulator (OmpR family)	-	LanI (57/71)
Wmc33	07320	NADPH-dependent oxidoreductase	WP_047179316 (100/100)	LanO (59/70)<br>LanZ4 (56/67)
Wmc34	07325	MFS transporter	WP_047179315 (99/99)	LanJ (50/66)

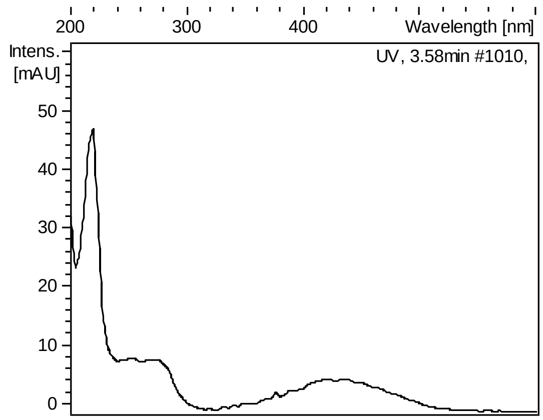

Supplementary Figure 25. UV-vis (DAD) spectrum of warkmycin CS1 (**22**).

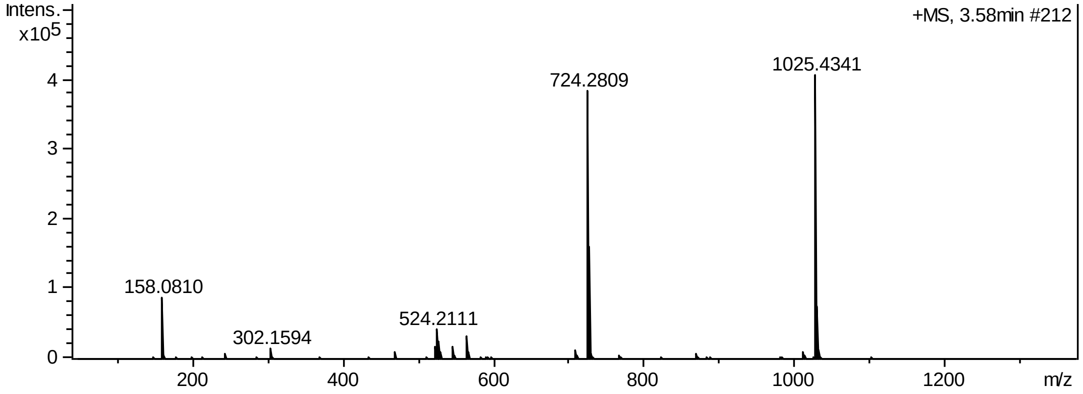

Supplementary Figure 26. HRMS spectrum of warkmycin CS1 (**22**).

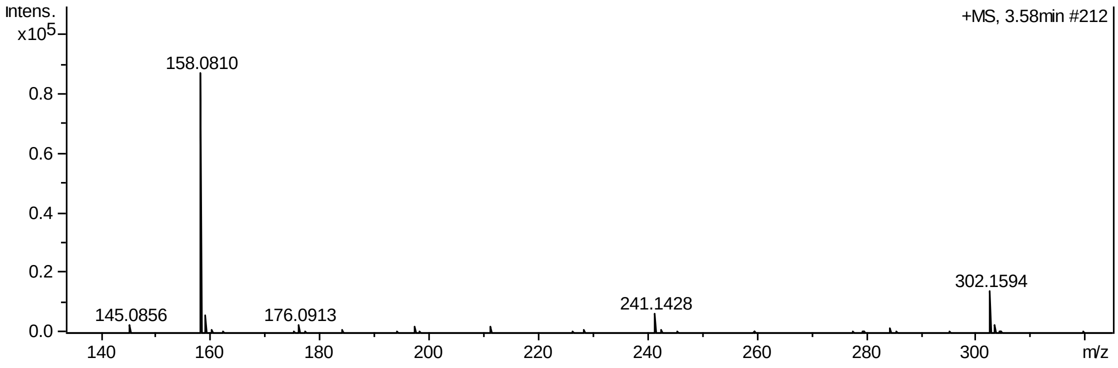

Supplementary Figure 27. Expansion of HRMS spectrum of warkmycin CS1 (**22**).

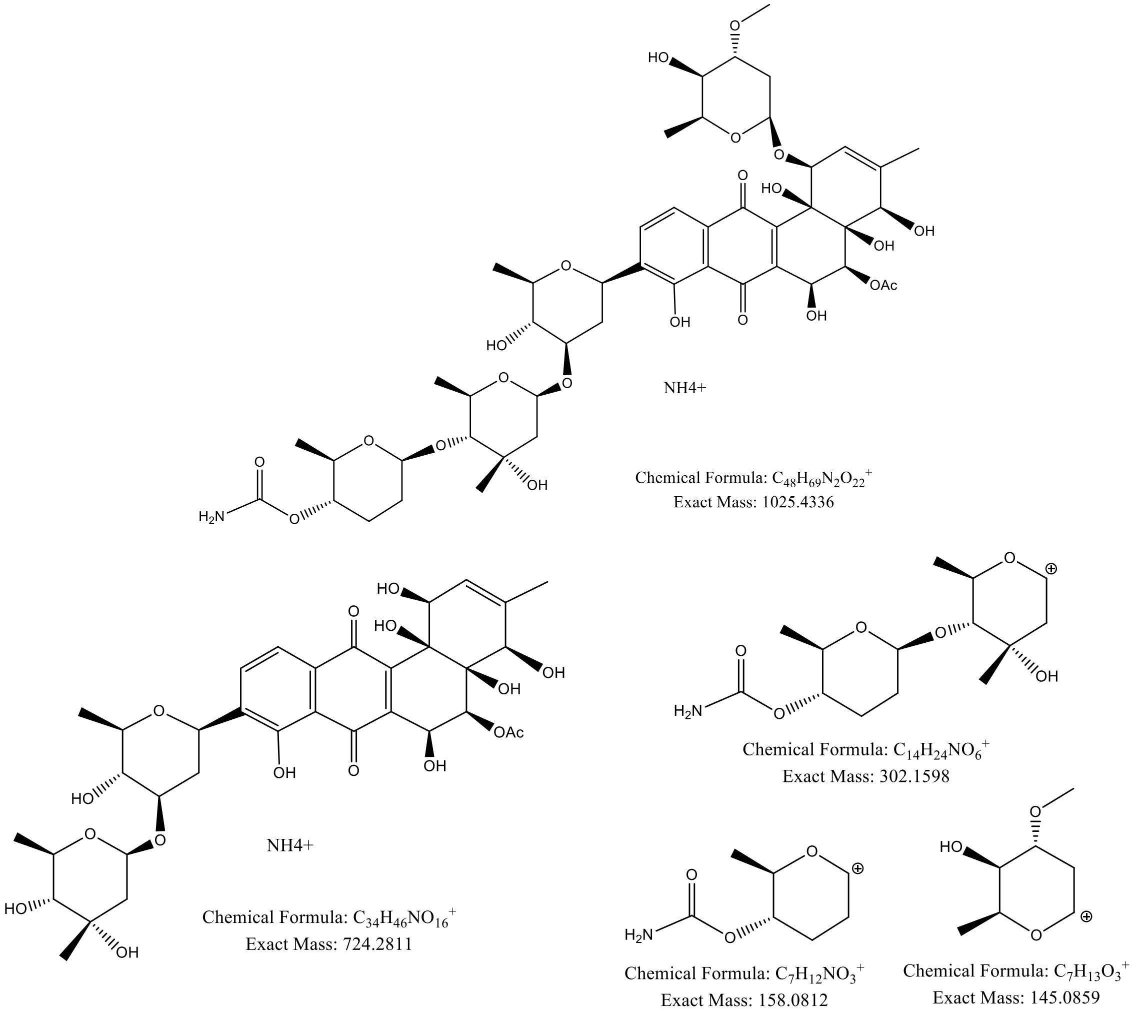

Supplementary Figure 28. In-source fragmentation of warkmycin CS1 (**22**).

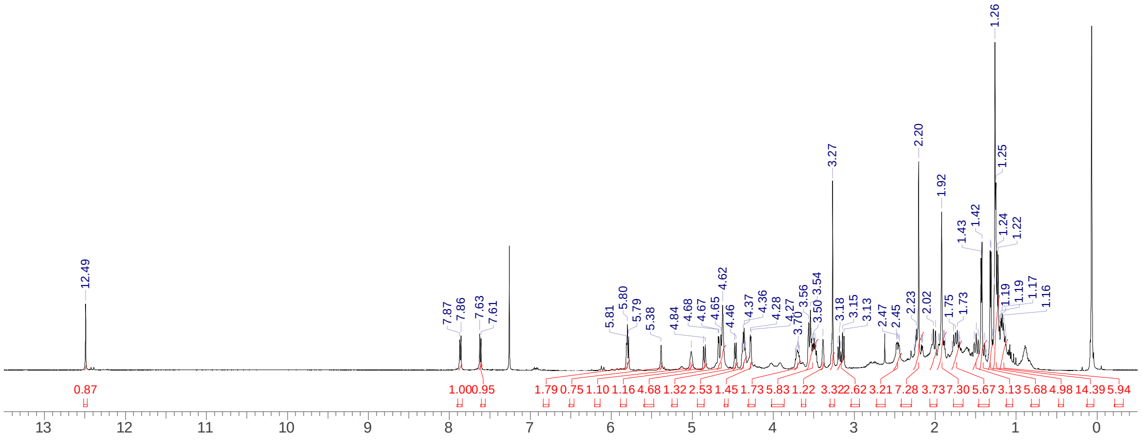

Supplementary Figure 29. ^{1}H NMR spectrum ($CDCl_3$, 500 MHz) of warkmycin CS1 (**22**).

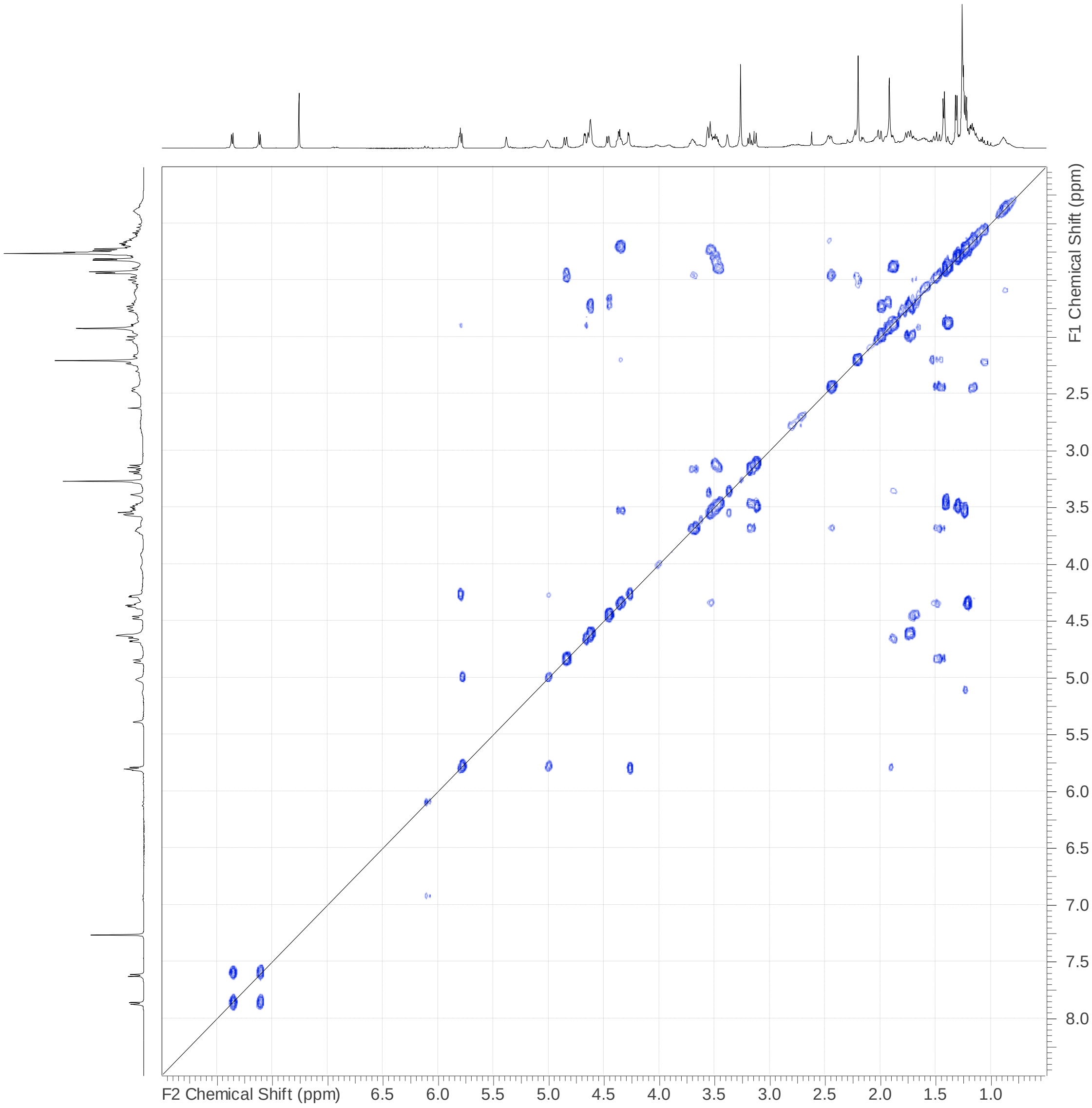

Supplementary Figure 30. COSY spectrum of warkmycin CS1 (**22**).

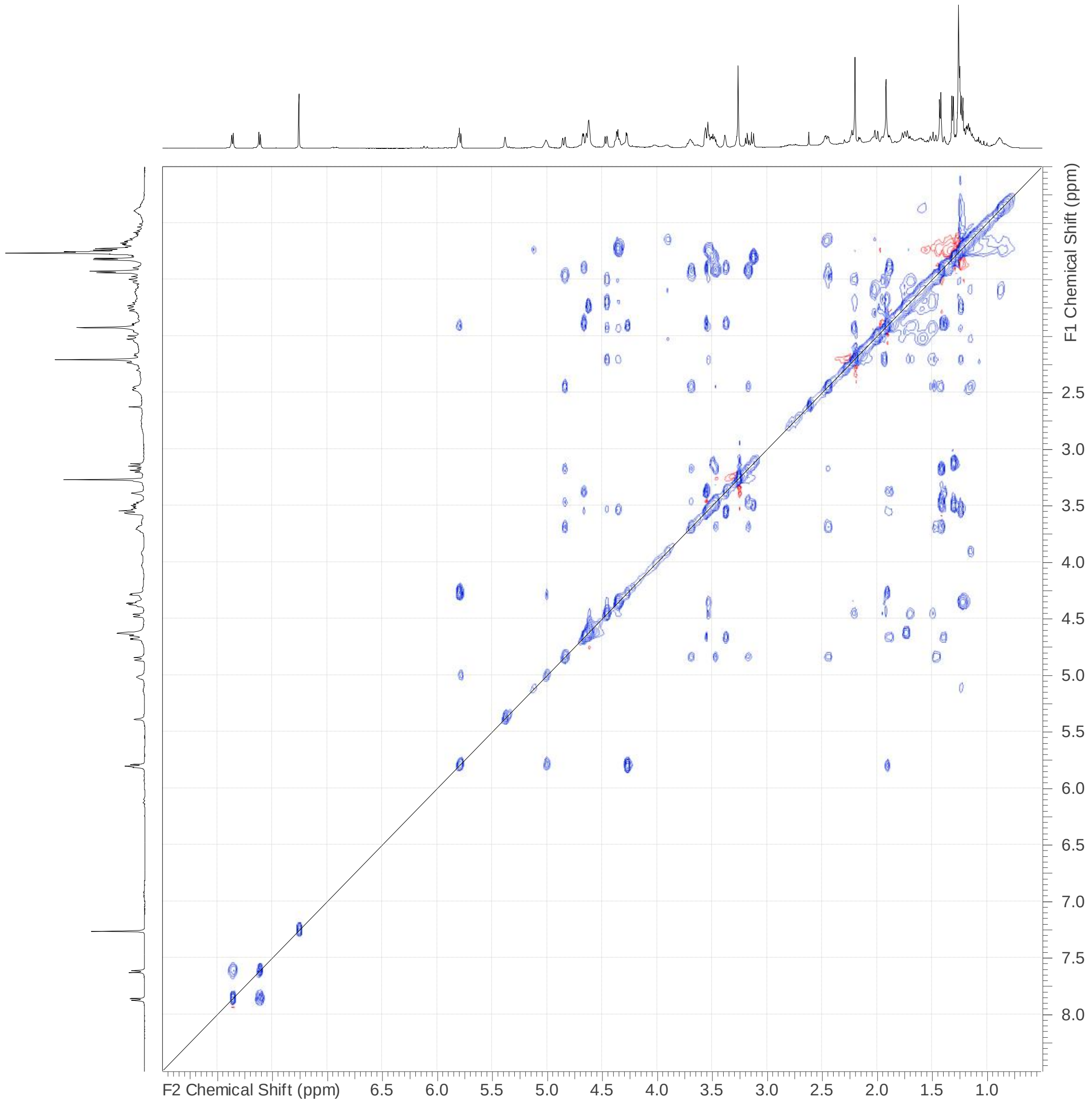

Supplementary Figure 31. TOCSY spectrum of warkmycin CS1 (**22**).

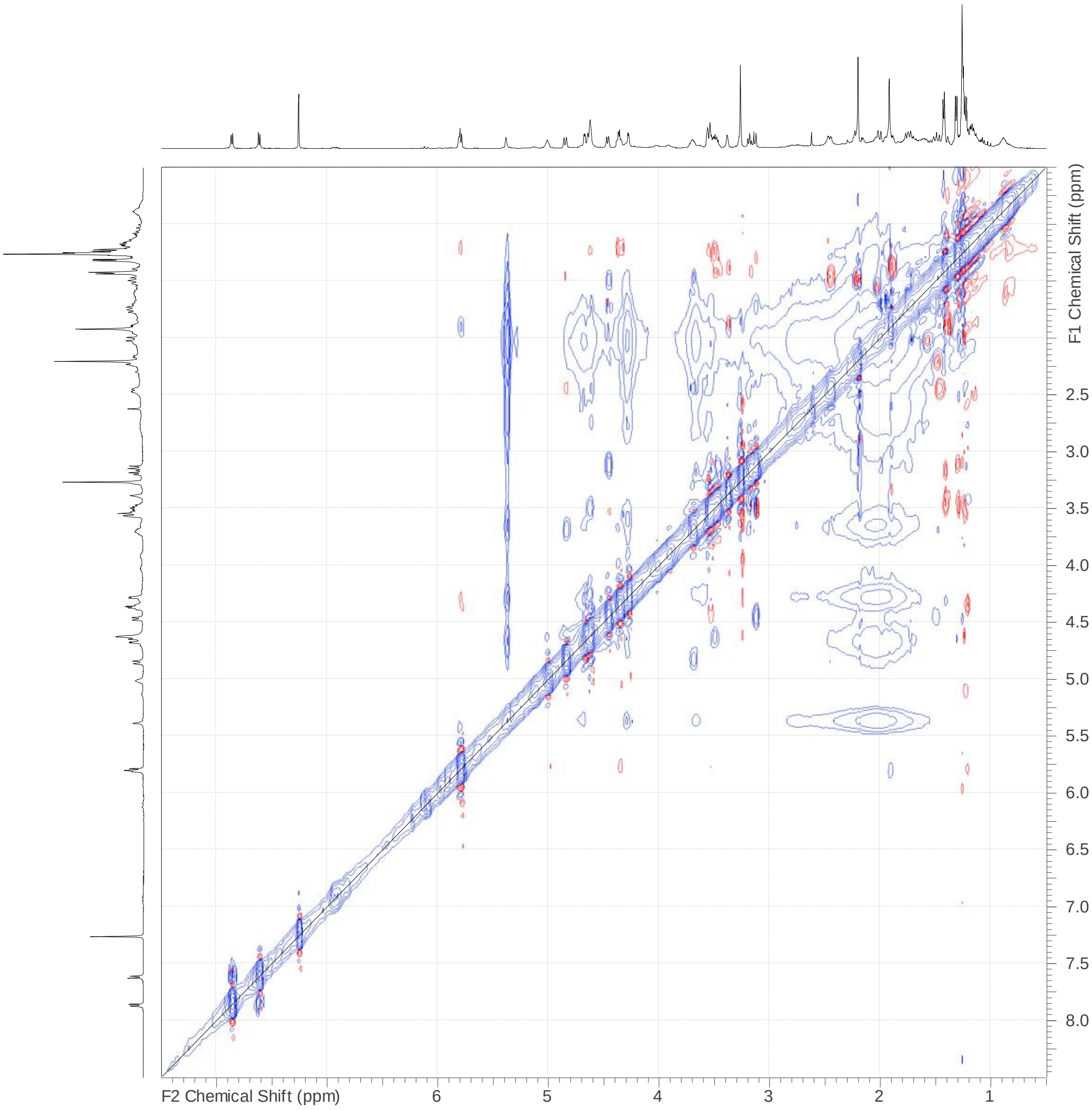

Supplementary Figure 32. NOESY spectrum of warkmycin CS1 (**22**).

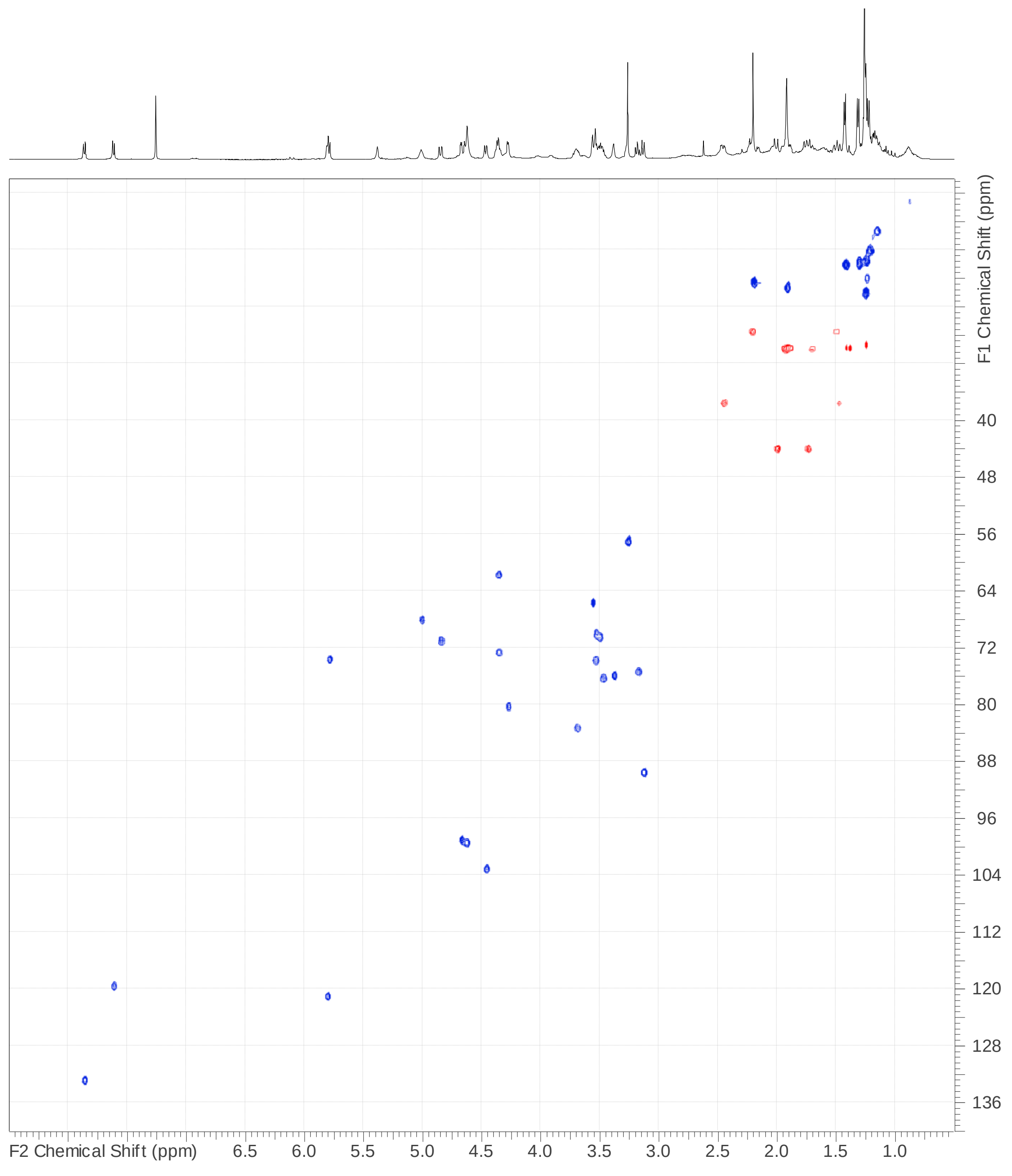

Supplementary Figure 33. HSQC spectrum of warkmycin CS1 (**22**).

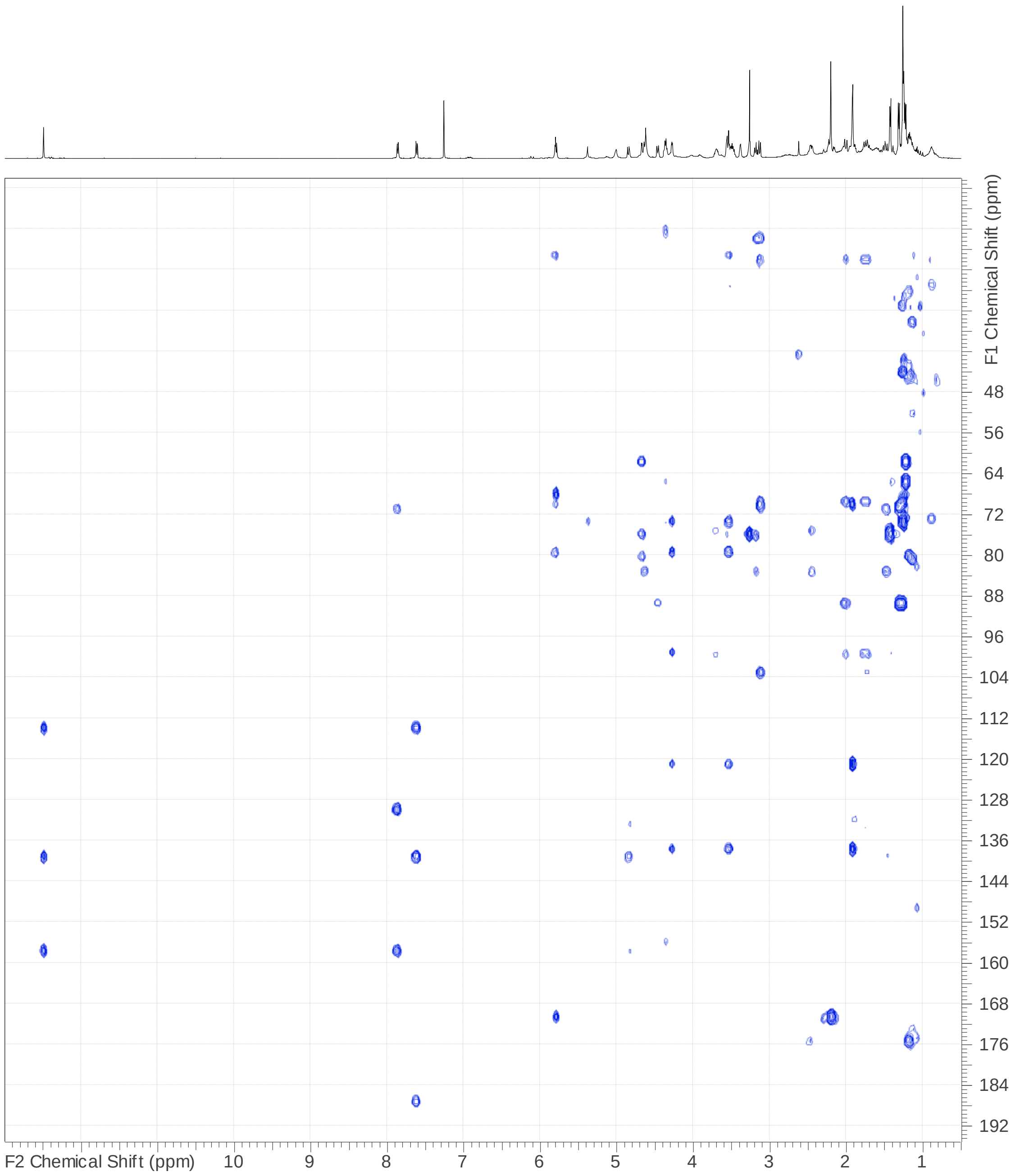

Supplementary Figure 34. HMBC spectrum of warkmycin CS1 (**22**).

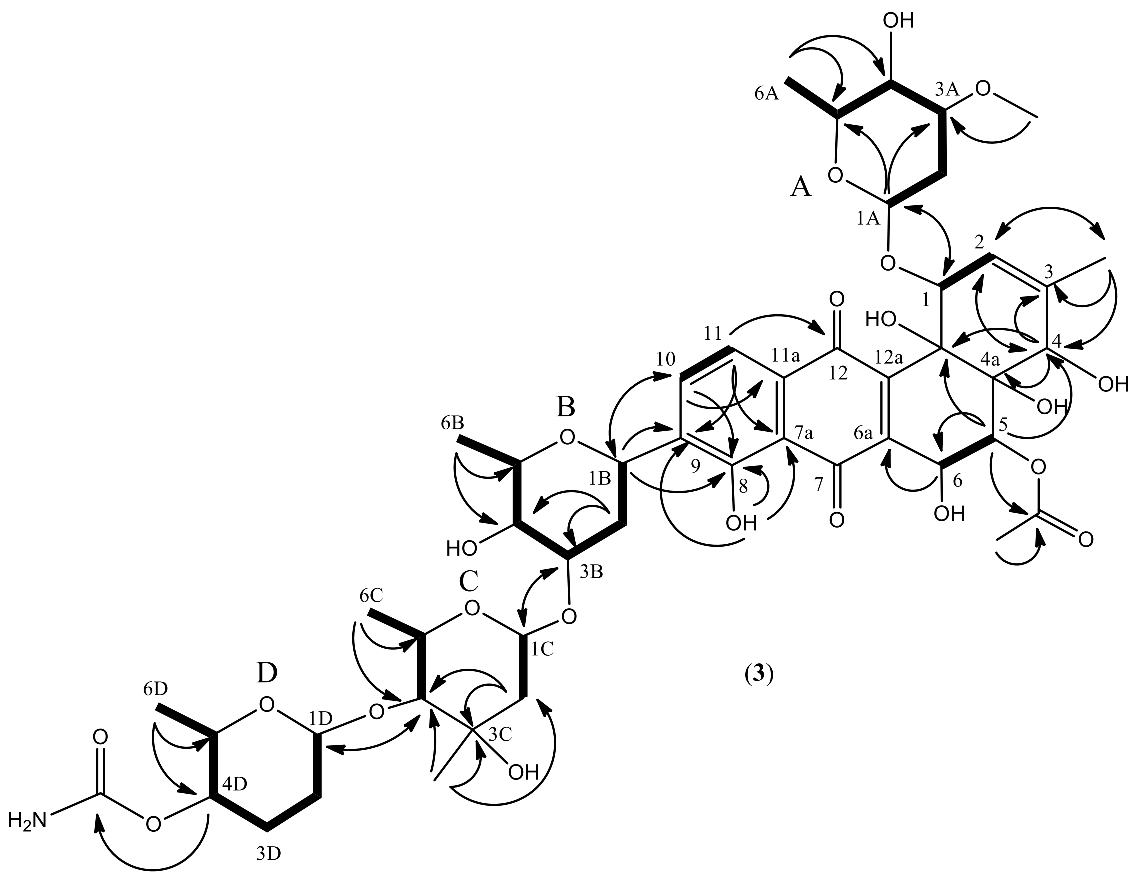

Supplementary Figure 35. Gross structure of warkmycin CS1 (**22**) determined by 2D-NMR. COSY correlations (further corroborated by the spin systems observed in the TOCSY spectrum) are indicated as bold bonds. Key HMBC correlations connecting independent spin systems are indicated by arrows.

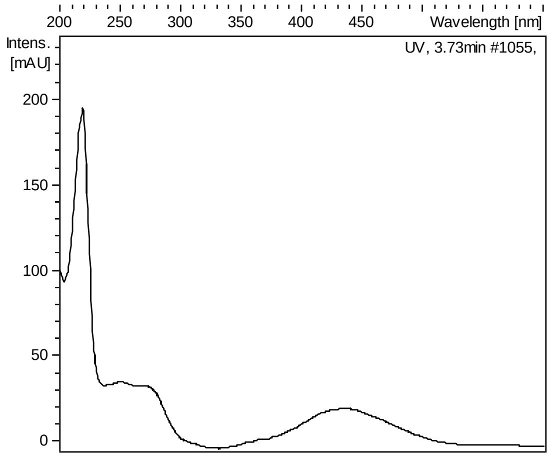

Supplementary Figure 36. UV-vis (DAD) spectrum of warkmycin CS2 (**24**).

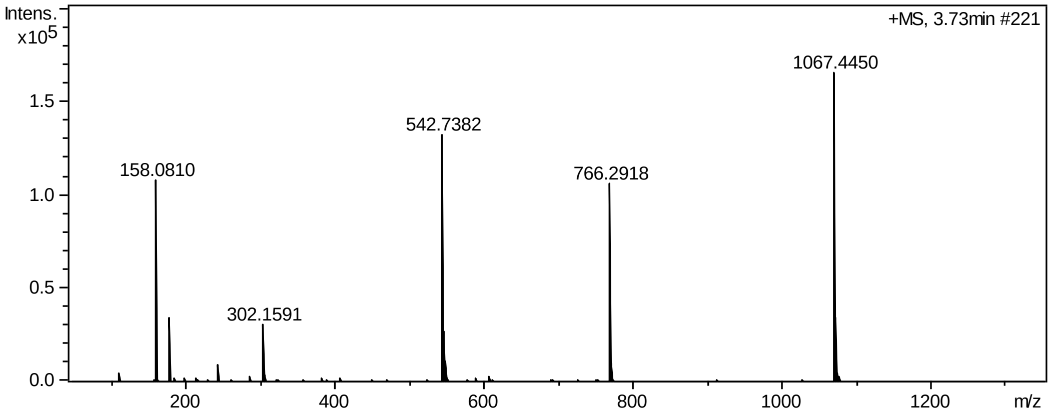

Supplementary Figure 37. HRMS spectrum of warkmycin CS2 (**24**).

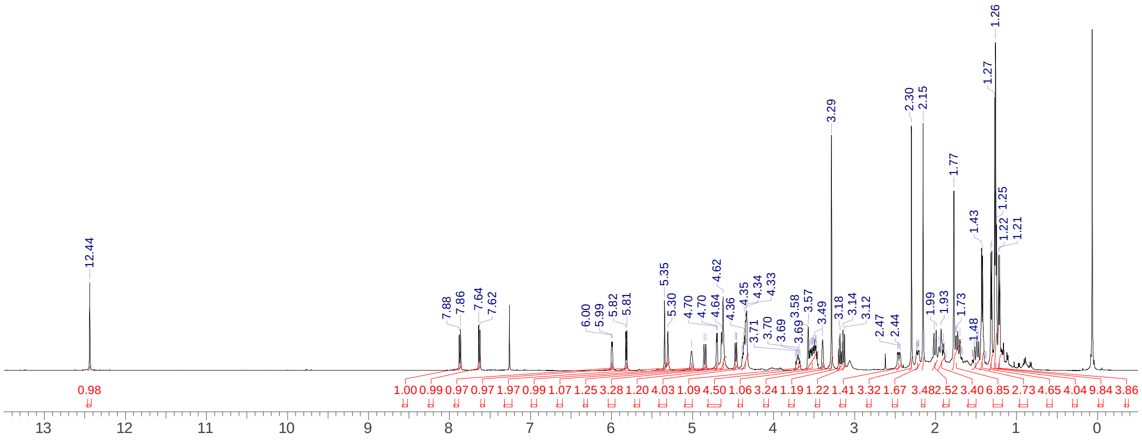

Supplementary Figure 38. ^{1}H NMR spectrum ($CDCl_3$, 500 MHz) of warkmycin CS2 (**24**).

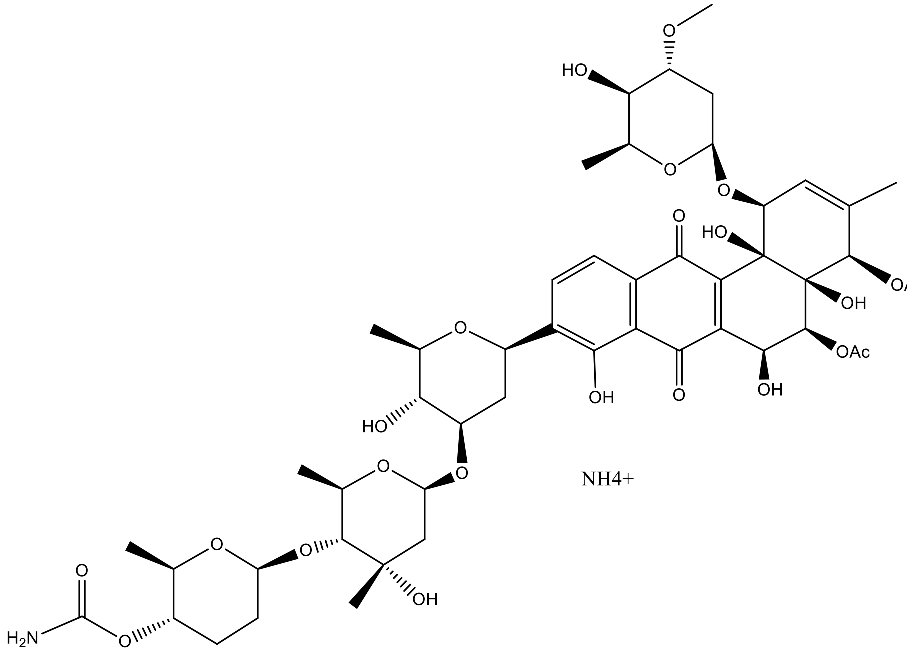

Chemical Formula: $C_{50}H_{71}N_2O_{23}^{+}$
Exact Mass: 1067.4442

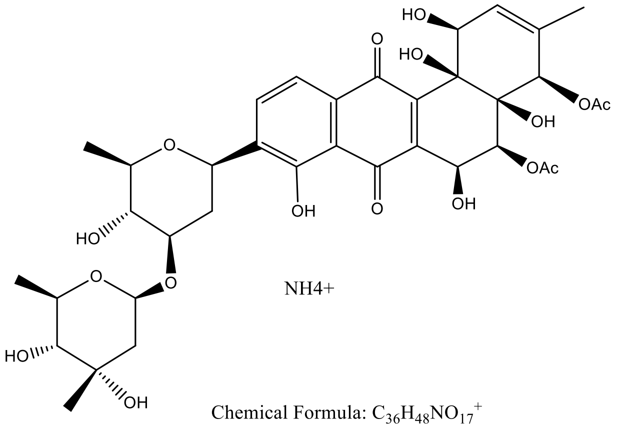

Chemical Formula: $C_{36}H_{48}NO_{17}^{+}$
Exact Mass: 766.2917

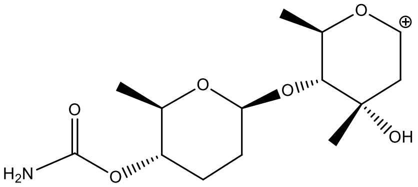

Chemical Formula: $C_{14}H_{24}NO_{6}^{+}$
Exact Mass: 302.1598

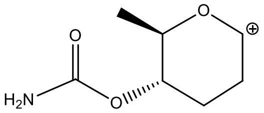

Chemical Formula: $C_{7}H_{12}NO_{3}^{+}$
Exact Mass: 158.0812

Supplementary Figure 39. In-source fragmentation of warkmycin CS2 (**24**)

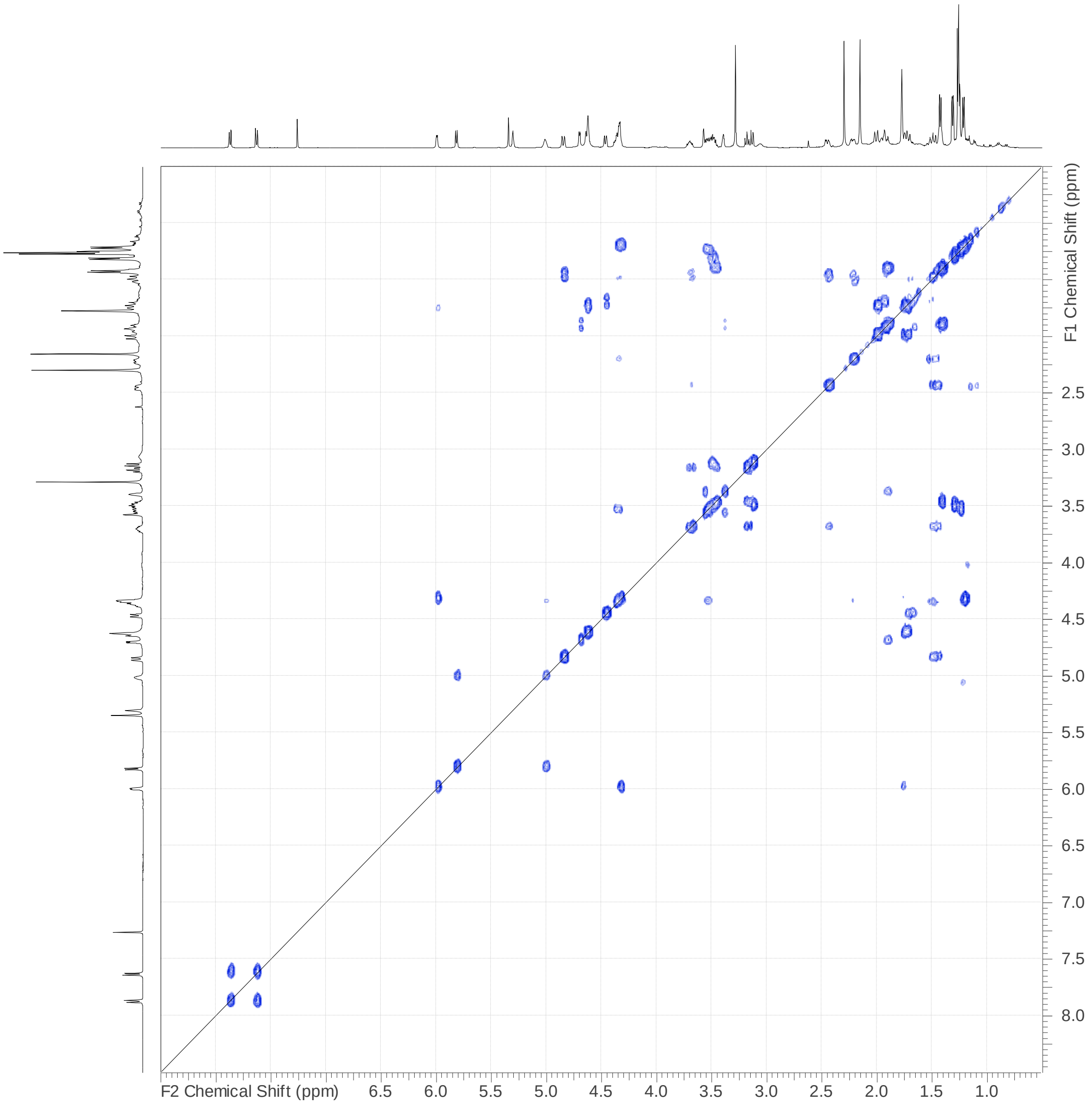

Supplementary Figure 40. COSY spectrum of warkmycin CS2 (**24**).

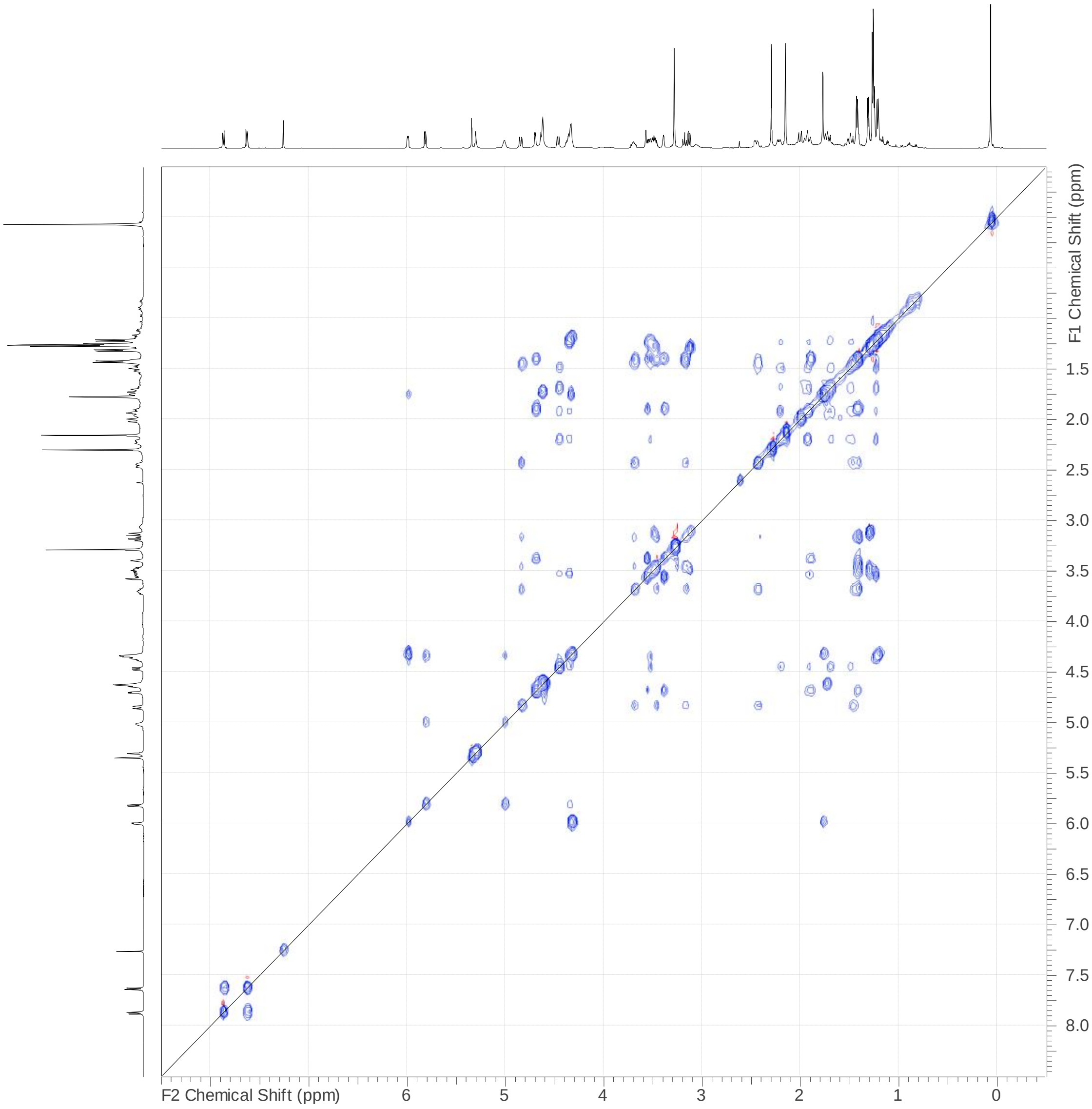

Supplementary Figure 41. TOCSY spectrum of warkmycin CS2 (**24**).

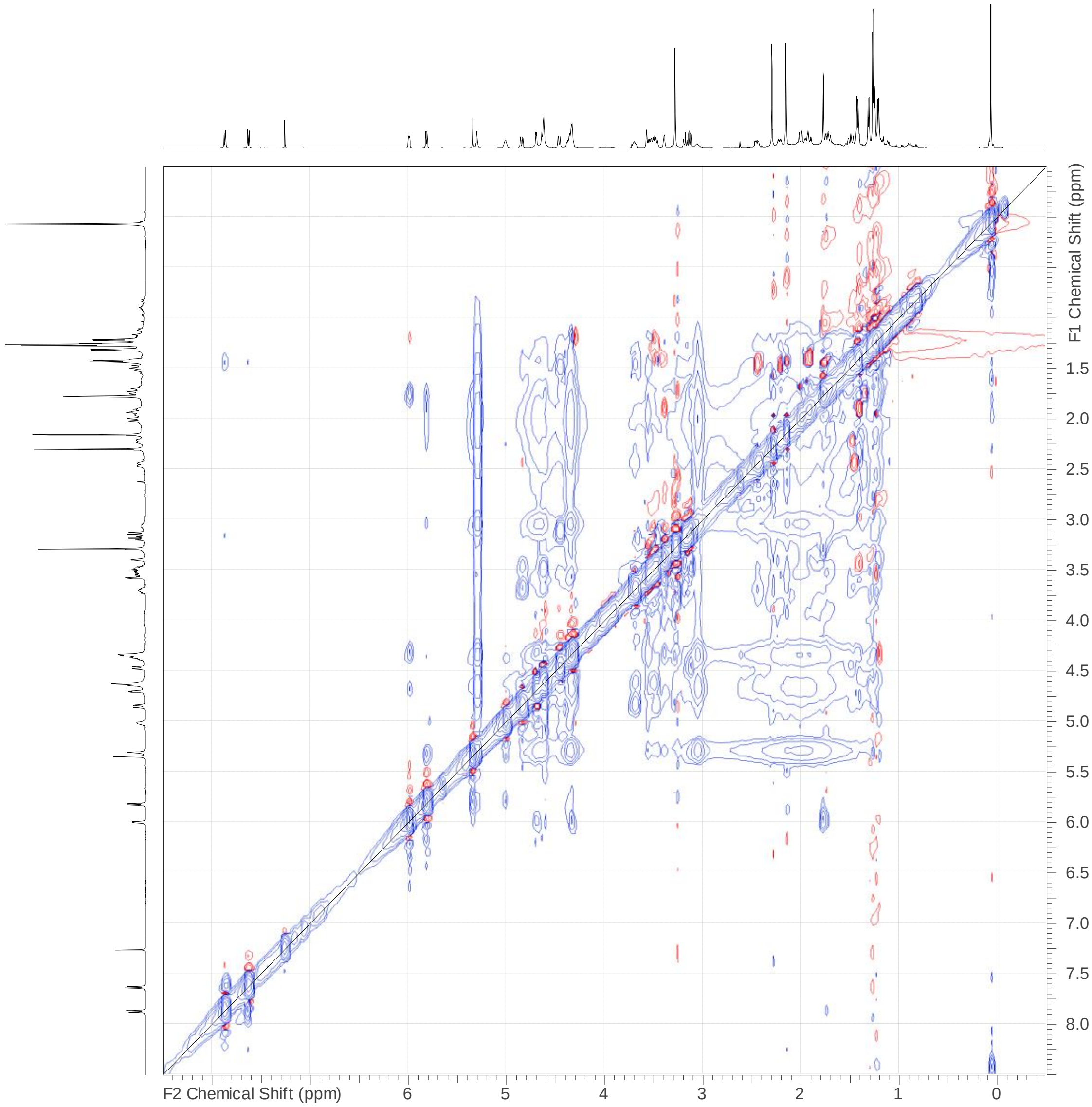

Supplementary Figure 42. NOESY spectrum of warkmycin CS2 (**24**).

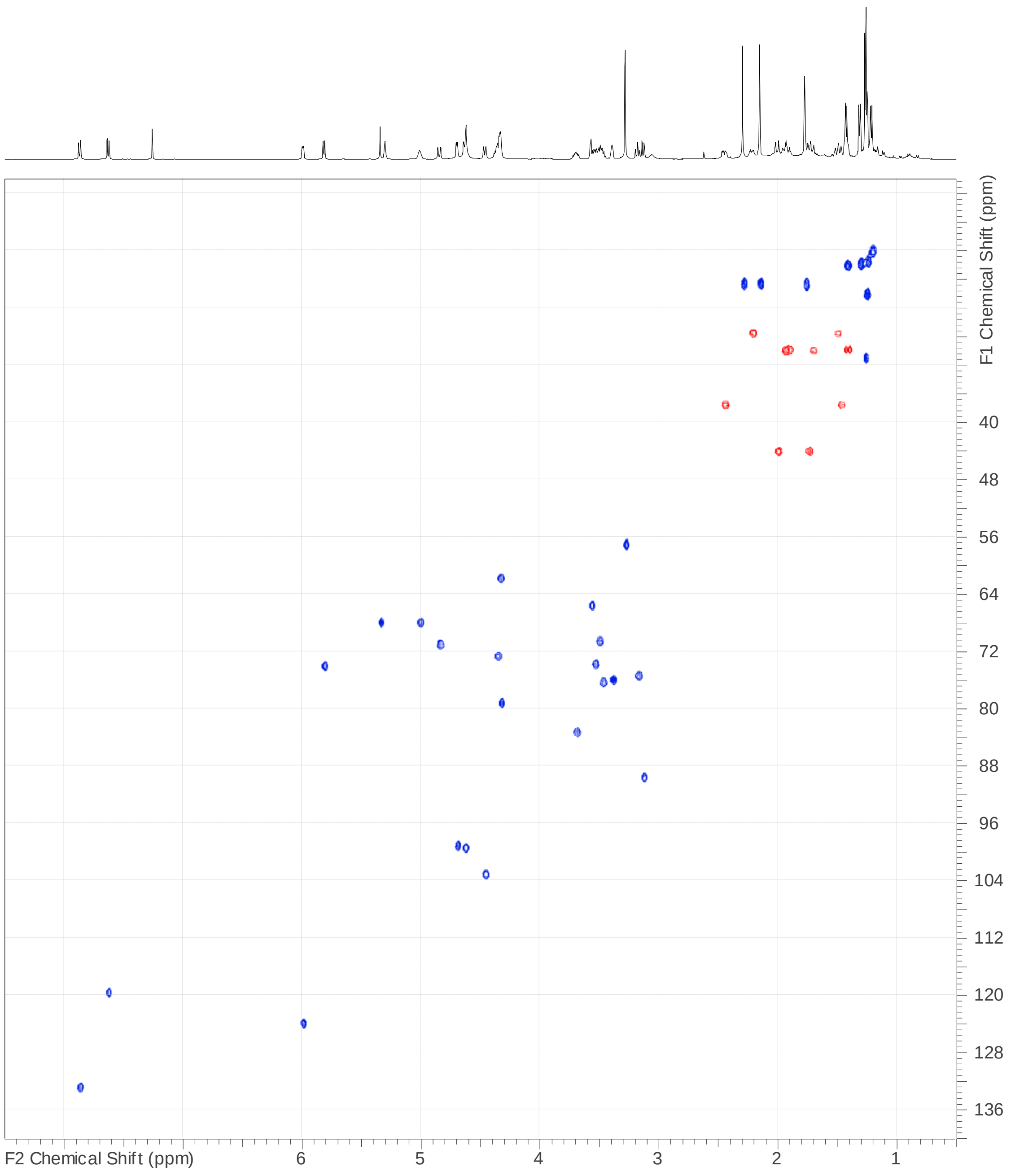

Supplementary Figure 43. HSQC spectrum of warkmycin CS2 (**24**).

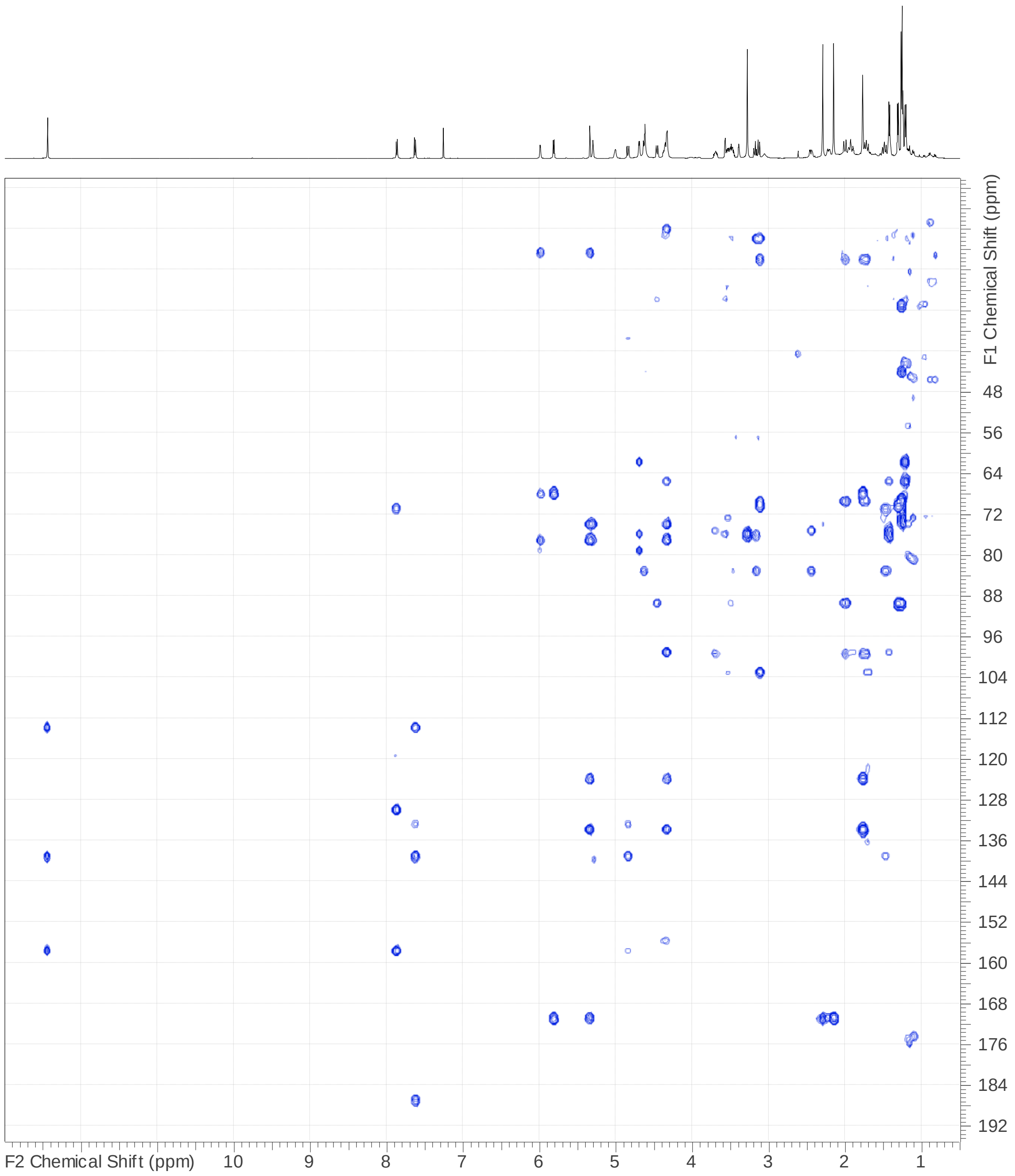

Supplementary Figure 44. HMBC spectrum of warkmycin CS2 (**24**).

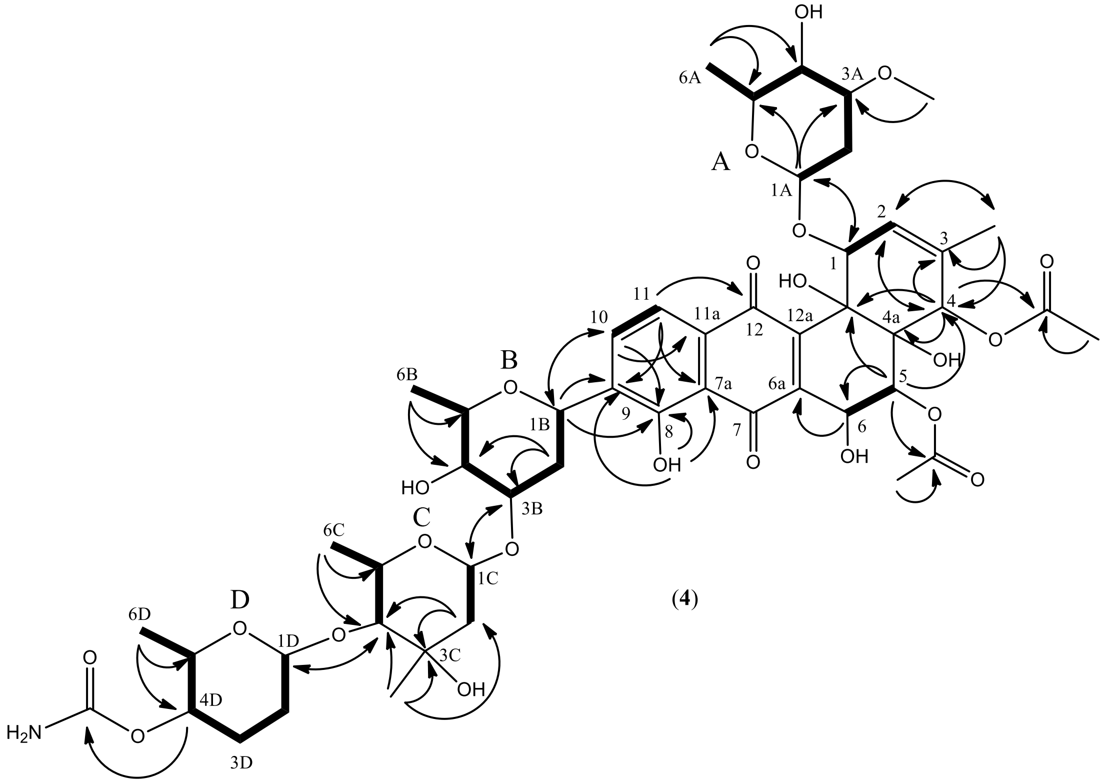

Supplementary Figure 45. Gross structure of warkmycin CS2 (**24**) determined by 2D-NMR. COSY correlations (further corroborated by the spin systems observed in the TOCSY spectrum) are indicated as bold bonds. Key HMBC correlations connecting independent spin systems are indicated by arrows.

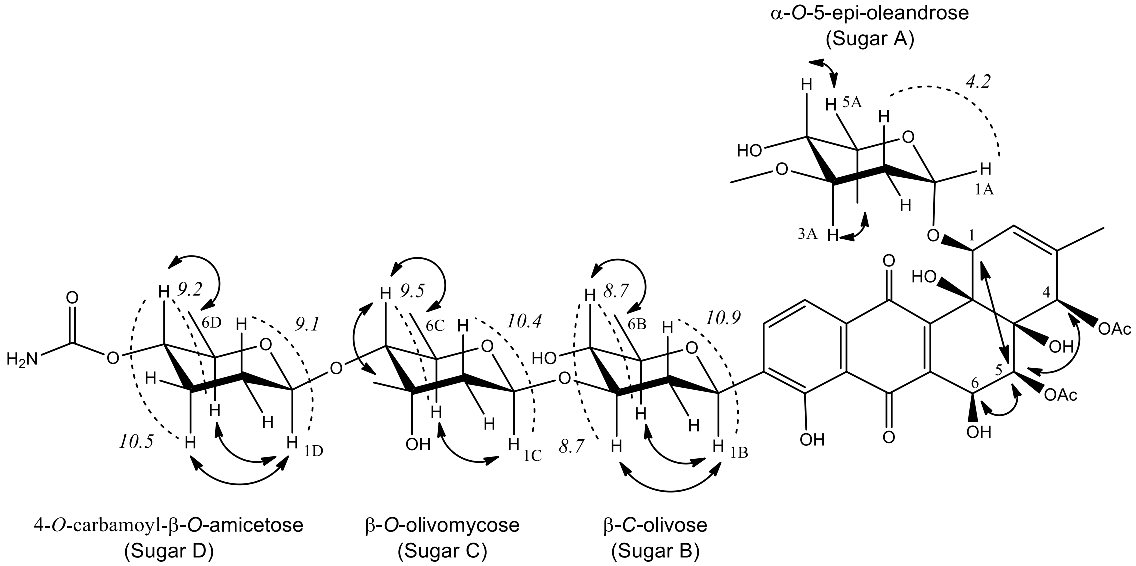

Supplementary Figure 46. Key NOESY correlations (solid arrows) of warkmycin CS2 (**24**) which, together with the observed coupling constants (dashed lines), allow stablishing the relative configuration of each monosaccharide and the aglycon. The same pattern is observed for warkmycin CS1 (**22**).

Supplementary Table 11. ^{1}H and ^{13}C NMR data for warkmycins CS1 (**22**) and CS2 (**24**) in $CDCl_3$ at 24 C°.

**Position**	**22**		**24**	
	δ_C**, type**	δ_H **(*J* in Hz)**	δ_C**, type**	δ_H **(*J* in Hz)**
1	80.5, CH	4.28, d (4.3)	79.4, CH	4.33, m
2	121.1, CH	5.81, br d (4.6)	124.1, CH	5.99, br d (4.5)
3	137.8, C	-	134.1, C	-
4	70.2, CH	3.54, s	68.2, CH	5.35, s
4a	73.5, C	-	74.2, C	-
5	73.9, CH	5.79, d (7.0)	74.2, C	5.82, d (6.5)
6	68.3, CH	5.01, br t	68.2, CH	5.01, br t
6a	n. d., C	-	n. d., C	-
7	n. d., C	-	n. d., C	-
7a	114.1, C	-	114.0, C	-
8	157.9, C	-	157.9, C	-
9	139.3, C	-	139.3, C	-
10	133.0, CH	7.86, d (7.8)	133.1, CH	7.87, d (7.8)
11	119.7, CH	7.62, d (7.8)	119.8, CH	7.63, d (7.8)
11a	130.1, C	-	130.1, C	-
12	187.3, C	-	187.2, C	-
12a	n. d., C	-	n. d., C	-
12b	79.6, C	-	77.3, C	-
Me-3	21.6, CH_3	1.92, s	21.0, CH_3	1.77, s
COMe-4	-	-	171.0, C<br>20.9, CH_3	 2.16, s
COMe-5	170.7, C<br>20.9, CH_3	 2.20, s	171.1, C<br>20.9, CH_3	 2.30, s
OH-8	-	12.49, s	-	12.44, s
Sugar A				
1A	99.3, CH	4.68, d (4.2)	99.3, CH	4.70, d (4.2)
2A	30.0, CH_2	1.41, 1.90, m	30.0, CH_2	1.43, 1.93, m
3A	76.1, CH	3.39, br s	76.1, CH	3.40, br s
4A	65.9, CH	3.56, m	65.9, CH	3.56, m
5A	61.8, CH	4.37, m	61.8, CH	4.34, m
6A	16.4, CH_3	1.23, d (6.7)	16.4, CH_3	1.22, d (6.7)
OMe-3	57.3, CH_3	3.27, s	57.3, CH_3	3.28, s
Sugar B				
1B	71.2, CH	4.85 (d, 10.9)	71.2, CH	4.85 (d, 10.9)
2B	37.8, CH_2	1.49, 2.46, m	37.8, CH_2	1.49, 2.46, m
3B	83.4, CH	3.70, m	83.4, CH	3.70, m
4B	75.5, CH	3.18, t (8.7)	75.5, CH	3.18, t (8.7)
5B	76.4, CH	3.48, m	76.4, CH	3.48, m
6B	18.3, CH_3	1.43, d (5.9)	18.3, CH_3	1.43, d (5.9)

Sugar C				
1C	99.6, CH	4.64, br d (10.4)	99.6, CH	4.64, br d (9.9)
2C	44.3, CH_2	1.75, 2.01, m	44.3, CH_2	1.75, 2.01, m
3C	69.7, C	-	69.7, C	-
4C	89.7, CH	3.14, d (9.5)	89.7, CH	3.14, d (9.5)
5C	70.7, CH	3.51, m	70.7, CH	3.51, m
6C	18.1, CH_3	1.32, d (5.9)	18.1, CH_3	1.32, d (5.9)
Me-3C	22.4, CH_3	1.26, s	22.4, CH_3	1.26, s
Sugar D				
1D	103.2, CH	4.47, br d (9.1)	103.2, CH	4.47, br d (9.1)
2D	30.3, CH_2	1.71, 1.94, m	30.3, CH_2	1.71, 1.94, m
3D	27.8, CH_2	1.51, 2.23, m	27.8, CH_2	1.51, 2.23, m
4D	72.8, CH	4.37, ddd (4.1, 9.2, 10.5)	72.8, CH	4.37, ddd (4.1, 9.2, 10.5)
5D	74.0, CH	3.54, m	74.0, CH	3.54, m
6D	17.7, CH_3	1.25, d (5.9)	17.7, CH_3	1.25, d (5.9)
$CONH_2$-4D	155.9, C	-	155.7, C	-

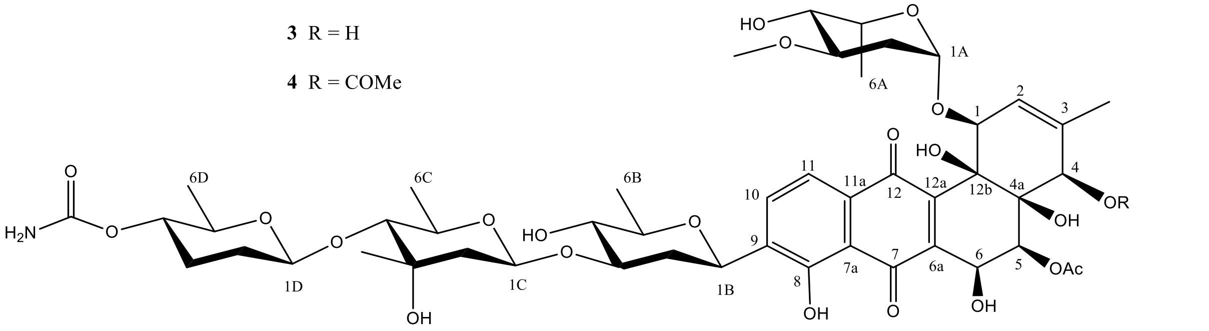

Supplementary Figure 47. Structure of warkmycin CS1 (**22**) and CS2 (**24**).

Supplementary Table 12. Oligonucleotides used in this work.

Name	Sequence (5´--- 3´)	Purpose	Reference
PCR-D1a	TAGAATTCCAGGCSACSWSSAACTACAC	Gene disruption	[64]
PCR-D1b	TATAAGCTTSWRGAASCGSCCSCCCTCCT	Gene disruption	[64]
AG5F	TAGAATTCGACTTCRTSATGTATCTSGGCGACAA	Gene disruption	[65]
4,6DH2	TATAAGCTTGRWRCTGRTRSGGCCGTAGTTGTT	Gene disruption	[66]
113-eSARP78.5b	TATGGATCCAGTTTCAAGTCGCACGGAAG	Cluster activation	This work
113-eSARP78.3	TATGAATTCCGCAGAGAAGCTGGGTAAAG	Cluster activation	This work
pErmE6RT15.5b	TAGGATCCACCACGGGGGAGGGCATGA	Cluster activation	This work
pErmE6RT15.3b	TAGAATTCTGGCCGACCGGGAGAGTTTG	Cluster activation	This work
227-eSARP2.5	TATGGATCCGGGTGGCTGAGAGAGACCA	Cluster activation	This work
227-eSARP2.3	TATGAATTCCCCTTCCTCTCCGTGCTC	Cluster activation	This work
227-eLuxR37.5	TATGGATCCTCGCCGAAATGTACTGTCCT	Cluster activation	This work
227-eLuxR37.3	TATGAATTCCCTCAGCTCACCTTGGACTG	Cluster activation	This work
57-eReg.5b	TATTCTAGAAAGACGGTAAGGCTGTTTGCT	Cluster activation	This work
57-eReg.3b	TATTCTAGAAGCACTCAGCCGCCGAAG	Cluster activation	This work
